# Large-Scale Recombinant Production of the SARS-CoV-2 Proteome for High-Throughput and Structural Biology Applications

**DOI:** 10.3389/fmolb.2021.653148

**Published:** 2021-05-10

**Authors:** Nadide Altincekic, Sophie Marianne Korn, Nusrat Shahin Qureshi, Marie Dujardin, Martí Ninot-Pedrosa, Rupert Abele, Marie Jose Abi Saad, Caterina Alfano, Fabio C. L. Almeida, Islam Alshamleh, Gisele Cardoso de Amorim, Thomas K. Anderson, Cristiane D. Anobom, Chelsea Anorma, Jasleen Kaur Bains, Adriaan Bax, Martin Blackledge, Julius Blechar, Anja Böckmann, Louis Brigandat, Anna Bula, Matthias Bütikofer, Aldo R. Camacho-Zarco, Teresa Carlomagno, Icaro Putinhon Caruso, Betül Ceylan, Apirat Chaikuad, Feixia Chu, Laura Cole, Marquise G. Crosby, Vanessa de Jesus, Karthikeyan Dhamotharan, Isabella C. Felli, Jan Ferner, Yanick Fleischmann, Marie-Laure Fogeron, Nikolaos K. Fourkiotis, Christin Fuks, Boris Fürtig, Angelo Gallo, Santosh L. Gande, Juan Atilio Gerez, Dhiman Ghosh, Francisco Gomes-Neto, Oksana Gorbatyuk, Serafima Guseva, Carolin Hacker, Sabine Häfner, Bing Hao, Bruno Hargittay, K. Henzler-Wildman, Jeffrey C. Hoch, Katharina F. Hohmann, Marie T. Hutchison, Kristaps Jaudzems, Katarina Jović, Janina Kaderli, Gints Kalniņš, Iveta Kaņepe, Robert N. Kirchdoerfer, John Kirkpatrick, Stefan Knapp, Robin Krishnathas, Felicitas Kutz, Susanne zur Lage, Roderick Lambertz, Andras Lang, Douglas Laurents, Lauriane Lecoq, Verena Linhard, Frank Löhr, Anas Malki, Luiza Mamigonian Bessa, Rachel W. Martin, Tobias Matzel, Damien Maurin, Seth W. McNutt, Nathane Cunha Mebus-Antunes, Beat H. Meier, Nathalie Meiser, Miguel Mompeán, Elisa Monaca, Roland Montserret, Laura Mariño Perez, Celine Moser, Claudia Muhle-Goll, Thais Cristtina Neves-Martins, Xiamonin Ni, Brenna Norton-Baker, Roberta Pierattelli, Letizia Pontoriero, Yulia Pustovalova, Oliver Ohlenschläger, Julien Orts, Andrea T. Da Poian, Dennis J. Pyper, Christian Richter, Roland Riek, Chad M. Rienstra, Angus Robertson, Anderson S. Pinheiro, Raffaele Sabbatella, Nicola Salvi, Krishna Saxena, Linda Schulte, Marco Schiavina, Harald Schwalbe, Mara Silber, Marcius da Silva Almeida, Marc A. Sprague-Piercy, Georgios A. Spyroulias, Sridhar Sreeramulu, Jan-Niklas Tants, Kaspars Tārs, Felix Torres, Sabrina Töws, Miguel Á. Treviño, Sven Trucks, Aikaterini C. Tsika, Krisztina Varga, Ying Wang, Marco E. Weber, Julia E. Weigand, Christoph Wiedemann, Julia Wirmer-Bartoschek, Maria Alexandra Wirtz Martin, Johannes Zehnder, Martin Hengesbach, Andreas Schlundt

**Affiliations:** ^1^ Institute for Organic Chemistry and Chemical Biology, Goethe University Frankfurt, Frankfurt am Main, Germany; ^2^ Center of Biomolecular Magnetic Resonance (BMRZ), Goethe University Frankfurt, Frankfurt am Main, Germany; ^3^ Institute for Molecular Biosciences, Goethe University Frankfurt, Frankfurt am Main, Germany; ^4^ Molecular Microbiology and Structural Biochemistry, UMR 5086, CNRS/Lyon University, Lyon, France; ^5^ Institute for Biochemistry, Goethe University Frankfurt, Frankfurt am Main, Germany; ^6^ Swiss Federal Institute of Technology, Laboratory of Physical Chemistry, ETH Zurich, Zurich, Switzerland; ^7^ Structural Biology and Biophysics Unit, Fondazione Ri.MED, Palermo, Italy; ^8^ National Center of Nuclear Magnetic Resonance (CNRMN, CENABIO), Federal University of Rio de Janeiro, Rio de Janeiro, Brazil; ^9^ Institute of Medical Biochemistry, Federal University of Rio de Janeiro, Rio de Janeiro, Brazil; ^10^ Multidisciplinary Center for Research in Biology (NUMPEX), Campus Duque de Caxias Federal University of Rio de Janeiro, Duque de Caxias, Brazil; ^11^ Institute for Molecular Virology, University of Wisconsin-Madison, Madison, WI, United States; ^12^ Institute of Chemistry, Federal University of Rio de Janeiro, Rio de Janeiro, Brazil; ^13^ Department of Chemistry, University of California, Irvine, CA, United States; ^14^ LCP, NIDDK, NIH, Bethesda, MD, United States; ^15^ Univ. Grenoble Alpes, CNRS, CEA, IBS, Grenoble, France; ^16^ Latvian Institute of Organic Synthesis, Riga, Latvia; ^17^ BMWZ and Institute of Organic Chemistry, Leibniz University Hannover, Hannover, Germany; ^18^ Group of NMR-Based Structural Chemistry, Helmholtz Centre for Infection Research, Braunschweig, Germany; ^19^ Multiuser Center for Biomolecular Innovation (CMIB), Department of Physics, São Paulo State University (UNESP), São José do Rio Preto, Brazil; ^20^ Institute of Pharmaceutical Chemistry, Goethe University Frankfurt, Frankfurt am Main, Germany; ^21^ Structural Genomics Consortium, Buchmann Institute for Molecular Life Sciences, Frankfurt am Main, Germany; ^22^ Department of Molecular, Cellular, and Biomedical Sciences, University of New Hampshire, Durham, NH, United States; ^23^ Department of Molecular Biology and Biochemistry, University of California, Irvine, CA, United States; ^24^ Magnetic Resonance Centre (CERM), University of Florence, Sesto Fiorentino, Italy; ^25^ Department of Chemistry “Ugo Schiff”, University of Florence, Sesto Fiorentino, Italy; ^26^ Department of Pharmacy, University of Patras, Patras, Greece; ^27^ Laboratory of Toxinology, Oswaldo Cruz Foundation (FIOCRUZ), Rio de Janeiro, Brazil; ^28^ Department of Molecular Biology and Biophysics, UConn Health, Farmington, CT, United States; ^29^ Signals GmbH & Co. KG, Frankfurt am Main, Germany; ^30^ Leibniz Institute on Aging—Fritz Lipmann Institute (FLI), Jena, Germany; ^31^ Latvian Biomedical Research and Study Centre, Riga, Latvia; ^32^ “Rocasolano” Institute for Physical Chemistry (IQFR), Spanish National Research Council (CSIC), Madrid, Spain; ^33^ Institute of Biophysical Chemistry, Goethe University Frankfurt, Frankfurt am Main, Germany; ^34^ IBG-4, Karlsruhe Institute of Technology, Karlsruhe, Germany; ^35^ Department of Biochemistry and National Magnetic Resonance Facility at Madison, University of Wisconsin-Madison, Madison, WI, United States; ^36^ Department of Biology, Technical University of Darmstadt, Darmstadt, Germany; ^37^ Institute of Biochemistry and Biotechnology, Charles Tanford Protein Centre, Martin Luther University Halle-Wittenberg, Halle/Saale, Germany

**Keywords:** COVID-19, SARS-CoV-2, nonstructural proteins, structural proteins, accessory proteins, intrinsically disordered region, cell-free protein synthesis, NMR spectroscopy

## Abstract

The highly infectious disease COVID-19 caused by the *Betacoronavirus* SARS-CoV-2 poses a severe threat to humanity and demands the redirection of scientific efforts and criteria to organized research projects. The international *COVID19-NMR* consortium seeks to provide such new approaches by gathering scientific expertise worldwide. In particular, making available viral proteins and RNAs will pave the way to understanding the SARS-CoV-2 molecular components in detail. The research in *COVID19-NMR* and the resources provided through the consortium are fully disclosed to accelerate access and exploitation. NMR investigations of the viral molecular components are designated to provide the essential basis for further work, including macromolecular interaction studies and high-throughput drug screening. Here, we present the extensive catalog of a holistic SARS-CoV-2 protein preparation approach based on the consortium’s collective efforts. We provide protocols for the large-scale production of more than 80% of all SARS-CoV-2 proteins or essential parts of them. Several of the proteins were produced in more than one laboratory, demonstrating the high interoperability between NMR groups worldwide. For the majority of proteins, we can produce isotope-labeled samples of HSQC-grade. Together with several NMR chemical shift assignments made publicly available on *covid19-nmr.com*, we here provide highly valuable resources for the production of SARS-CoV-2 proteins in isotope-labeled form.

## Introduction

Severe acute respiratory syndrome coronavirus 2 (SARS-CoV-2, SCoV2) is the cause of the early 2020 pandemic coronavirus lung disease 2019 (COVID-19) and belongs to *Betacoronaviruses*, a genus of the Coronaviridae family covering the α−δ genera (Leao et al., 2020). The large RNA genome of SCoV2 has an intricate, highly condensed arrangement of coding sequences (Wu et al., 2020). Sequences starting with the main start codon contain an open reading frame 1 (ORF1), which codes for two distinct, large polypeptides (pp), whose relative abundance is governed by the action of an RNA pseudoknot structure element. Upon RNA folding, this element causes a −1 frameshift to allow the continuation of translation, resulting in the generation of a 7,096-amino acid 794 kDa polypeptide. If the pseudoknot is not formed, expression of the first ORF generates a 4,405-amino acid 490 kDa polypeptide. Both the short and long polypeptides translated from this ORF (pp1a and pp1ab, respectively) are posttranslationally cleaved by virus-encoded proteases into functional, nonstructural proteins (nsps). ORF1a encodes eleven nsps, and ORF1ab additionally encodes the nsps 12–16. The downstream ORFs encode structural proteins (S, E, M, and N) that are essential components for the synthesis of new virus particles. In between those, additional proteins (accessory/auxiliary factors) are encoded, for which sequences partially overlap (Finkel et al., 2020) and whose identification and classification are a matter of ongoing research (Nelson et al., 2020; Pavesi, 2020). In total, the number of identified peptides or proteins generated from the viral genome is at least 28 on the evidence level, with an additional set of smaller proteins or peptides being predicted with high likelihood.

High-resolution studies of SCoV and SCoV2 proteins have been conducted using all canonical structural biology approaches, such as X-ray crystallography on proteases (Zhang et al., 2020) and methyltransferases (MTase) (Krafcikova et al., 2020), cryo-EM of the RNA polymerase (Gao et al., 2020; Yin et al., 2020), and liquid-state (Almeida et al., 2007; Serrano et al., 2009; Cantini et al., 2020; Gallo et al., 2020; Korn et al., 2020a; Korn et al., 2020b; Kubatova et al., 2020; Tonelli et al., 2020) and solid-state NMR spectroscopy of transmembrane (TM) proteins (Mandala et al., 2020). These studies have significantly improved our understanding on the functions of molecular components, and they all rely on the recombinant production of viral proteins in high amount and purity.

Apart from structures, purified SCoV2 proteins are required for experimental and preclinical approaches designed to understand the basic principles of the viral life cycle and processes underlying viral infection and transmission. Approaches range from studies on immune responses (Esposito et al., 2020), antibody identification (Jiang et al., 2020), and interactions with other proteins or components of the host cell (Bojkova et al., 2020; Gordon et al., 2020). These examples highlight the importance of broad approaches for the recombinant production of viral proteins.

The research consortium *COVID19-NMR* founded in 2020 seeks to support the search for antiviral drugs using an NMR-based screening approach. This requires the large-scale production of all druggable proteins and RNAs and their NMR resonance assignments. The latter will enable solution structure determination of viral proteins and RNAs for rational drug design and the fast mapping of compound binding sites. We have recently produced and determined secondary structures of SCoV2 RNA *cis*-regulatory elements in near completeness by NMR spectroscopy, validated by DMS-MaPseq (Wacker et al., 2020), to provide a basis for RNA-oriented fragment screens with NMR.

We here compile a compendium of more than 50 protocols (see Supplementary Tables SI1–SI23) for the production and purification of 23 of the 30 SCoV2 proteins or fragments thereof (summarized in Tables 1, 2). We defined those 30 proteins as existing or putative ones to our current knowledge (see later discussion). This compendium has been generated in a coordinated and concerted effort between >30 labs worldwide (Supplementary Table S1), with the aim of providing pure mg amounts of SCoV2 proteins. Our protocols include the rational strategy for construct design (if applicable, guided by available homolog structures), optimization of expression, solubility, yield, purity, and suitability for follow-up work, with a focus on uniform stable isotope-labeling.

**TABLE 1:** SCoV2 protein constructs expressed and purified, given with the genomic position and corresponding PDBs for construct design.

Protein genome position (nt)^a^	Trivial name construct expressed	Size (aa)	Boundaries	MW (kDa)	Homol. SCoV (%)^b^	Template PDB^c^	SCoV2 PDB^d^
**nsp1**	**Leader**	**180**		**19.8**	**84**		
*266–805*							
	Full-length	180	1–180	19.8	83		
	Globular domain (GD)	116	13–127	12.7	85	2GDT	7K7P
**nsp2**		**638**		**70.5**	**68**		
*806–2,719*							
	C-terminal IDR (CtDR)	45	557–601	4.9	55		
**nsp3**		**1,945**		**217.3**	**76**		
*2,720–8,554*							
a	Ub-like (Ubl) domain	111	1–111	12.4	79	2IDY	7KAG
a	Ub-like (Ubl) domain + IDR	206	1–206	23.2	58		
b	Macrodomain	170	207–376	18.3	74	6VXS	6VXS
c	SUD-N	140	409–548	15.5	69	2W2G	
c	SUD-NM	267	409–675	29.6	74	2W2G	
c	SUD-M	125	551–675	14.2	82	2W2G	
c	SUD-MC	195	551–743	21.9	79	2KQV	
c	SUD-C	64	680–743	7.4	73	2KAF	
d	Papain-like protease PL^pro^	318	743–1,060	36	83	6W9C	6W9C
e	NAB	116	1,088–1,203	13.4	87	2K87	
Y	CoV-Y	308	1,638–1,945	34	89		
**nsp5**	**Main protease (M^pro^)**	**306**		**33.7**	**96**		
*10,055–10,972*							
	Full-length^e^	306	1–306	33.7	96	6Y84	6Y84
**nsp7**		**83**		**9.2**	**99**		
*11,843–12,091*							
	Full-length	83	1–83	9.2	99	6WIQ	6WIQ
**nsp8**		**198**		**21.9**	**98**		
*12,092–12,685*							
	Full-length	198	1–198	21.9	97	6WIQ	6WIQ
**nsp9**		**113**		**12.4**	**97**		
*12,686–13,024*							
	Full-length	113	1–113	12.4	97	6W4B	6W4B
**nsp10**		**139**		**14.8**	**97**		
*13,025–13,441*							
	Full-length	139	1–139	14.8	97	6W4H	6W4H
**nsp13**	**Helicase**	**601**		**66.9**	**100**		
*16,237–18,039*							
	Full-length	601	1–601	66.9	100	6ZSL	6ZSL
**nsp14**	**Exonuclease/methyltransferase**	**527**		**59.8**	**95**		
*18,040–19,620*							
	Full-length	527	1–527	59.8	95	5NFY	
	MTase domain	240	288–527	27.5	95		
**nsp15**	**Endonuclease**	**346**		**38.8**	**89**		
*19,621–20,658*							
	Full-length	346	1–346	38.8	89	6W01	6W01
**nsp16**	**Methyltransferase**	**298**		**33.3**	**93**		
*20,659–21,552*							
	Full-length	298	1–298	33.3	93	6W4H	6W4H
**ORF3a**		**275**		**31.3**	**72**		
*25,393–26,220*							
	Full-length	275	1–275	31.3	72	6XDC	6XDC
**ORF4**	**Envelope (E) protein**	**75**		**8.4**	**95**		
*26,245–26,472*							
	Full-length	75	1–75	8.4	95	5X29	7K3G
**ORF5**	**Membrane glycoprotein (M)**	**222**		**25.1**	**91**		
*26,523–27,387*							
	Full-length	222	1–222	25.1	91		
**ORF6**		**61**		**7.3**	**69**		
*27,202–27,387*							
	Full-length	61	1–61	7.3	69		
**ORF7a**		**121**		**13.7**	**85**		
*27,394–27,759*							
	Ectodomain (ED)	66	16–81	7.4	85	1XAK	6W37
**ORF7b**		**43**		**5.2**	**85**		
*27,756–27,887*							
	Full-length	43	1–43	5.2	85		
**ORF8**		**121**		**13.8**	**32**		
*27,894–28,259*							
ORF8	Full-length	121	1–121	13.8	32		
ΔORF8	w/o signal peptide	106	16–121	12	41	7JTL	7JTL
**ORF9a**	**Nucleocapsid (N)**	**419**		**45.6**	**91**		
*28,274–29,533*							
	IDR1-NTD-IDR2	248	1–248	26.5	90		
	NTD-SR	169	44–212	18.1	92		
	NTD	136	44–180	14.9	93	6YI3	6YI3
	CTD	118	247–364	13.3	96	2JW8	7C22
**ORF9b**		**97**		**10.8**	**72**		
*28,284–28,574*							
	Full-length	97	1–97	10.8	72	6Z4U	6Z4U
**ORF14**		**73**		**8**	**n.a**		
*28,734–28,952*							
	Full-length	73	1–73	8	n.a		
**ORF10**		**38**		**4.4**	**29**		
*29,558–29,674*							
	Full-length	38	1–38	4.4	29		

^a^ Genome position in nt corresponding to SCoV2 NCBI reference genome entry NC_045512.2, identical to GenBank entry MN908947.3.

^b^ Sequence identities to SCoV are calculated from an alignment with corresponding protein sequences based on the genome sequence of NCBI Reference NC_004718.3.

^c^ Representative PDB that was available at the beginning of construct design, either SCoV or SCoV2.

^d^ Representative PDB available for SCoV2 (as of December 2020).

^e^ Additional point mutations in fl-construct have been expressed.

n.a.: not applicable.

**TABLE 2:** Summary of SCoV2 protein production results in *Covid19-NMR*.

Construct expressed	Yields (mg/L)^a^ or *(mg/ml)* ^b^	Results	Comments	BMRB	Supplementary Material
**nsp1**					SI1
fl	5	NMR assigned	Expression only at >20°C; after 7 days at 25°C partial proteolysis	50620^d^	
GD	>0.5	HSQC	High expression; mainly insoluble; higher salt increases stability (>250 mM)		
**nsp2**					SI2
CtDR	0.7–1.5	NMR assigned	Assignment with His-tag shown in (Mompean et al., 2020)	50687^c^	
**nsp3**					SI3
UBl	0.7	HSQC	Highly stable over weeks; spectrum overlays with Ubl + IDR		
UBl + IDR	2–3	NMR assigned	Highly stable for >2 weeks at 25°C	50446^d^	
Macrodomain	9	NMR assigned	Highly stable for >1 week at 25°C and > 2 weeks at 4°C	50387^d^	
				50388^d^	
SUD-N	14	NMR assigned	Highly stable for >10 days at 25°C	50448^d^	
SUD-NM	17	HSQC	Stable for >1 week at 25°C		
SUD-M	8.5	NMR assigned	Significant precipitation during measurement; tendency to dimerize	50516^d^	
SUD-MC	12	HSQC	Stable for >1 week at 25°C		
SUD-C	4.7	NMR assigned	Stable for >10 days at 25°C	50517^d^	
PL^pro^	12	HSQC	Solubility-tag essential for expression; tendency to aggregate		
NAB	3.5	NMR assigned	Highly stable for >1 week at 25°C; stable for >5 weeks at 4°C	50334^d^	
CoV-Y	12	HSQC	Low temperature (<25°C) and low concentrations (<0.2 mM) favor stability; gradual degradation at 25°C; lithium bromide in final buffer supports solubility		
**nsp5**					SI4
fl	55	HSQC	Impaired dimerization induced by artificial N-terminal residues		
**nsp7**					SI5
fl	17	NMR assigned	Stable for several days at 35°C; stable for >1 month at 4°C	50337^d^	
**nsp8**					SI6
fl	17	HSQC	Concentration dependent aggregation; low concentrations favor stability		
**nsp9**					SI7
fl	4.5	NMR assigned	Stable dimer for >4 months at 4°C and >2 weeks at 25°C	50621^d^	
				50622^d^	
				50513	
**nsp10**					SI8
fl	15	NMR assigned	Zn^2+^ addition during expression and purification increases protein stability; stable for >1 week at 25°C	50392	
**nsp13**					SI9
fl	0.5	HSQC	Low expression; protein unstable; concentration above 20 µM not possible		
**nsp14**					SI10
fl	6	Pure protein	Not above 50 µM; best storage: with 50% (v/v) glycerol; addition of reducing agents		
MTase	10	Pure protein	As fl nsp14; high salt (>0.4 M) for increased stability; addition of reducing agents		
**nsp15**					SI11
fl	5	HSQC	Tendency to aggregate at 25°C		
**nsp16**					SI12
fl	10	Pure protein	Addition of reducing agents; 5% (v/v) glycerol favorable; highly unstable		
**ORF3a**					SI13
fl	*0.6*	Pure protein	Addition of detergent during expression (0.05% Brij-58); stable protein		
**E protein**					SI14
fl	*0.45*	Pure protein	Addition of detergent during expression (0.05% Brij-58); stable protein		
**M Protein**					SI15
fl	*0.33*	Pure protein	Addition of detergent during expression (0.05% Brij-58); stable protein		
**ORF6**					SI16
fl	*0.27*	HSQC	Soluble expression without detergent; stable protein; no expression with STREP-tag at N-terminus		
**ORF7** **a**					SI17
ED	0.4	HSQC	Unpurified protein tends to precipitate during refolding, purified protein stable for 4 days at 25°C		
**ORF7** **b**					SI18
fl	0.6	HSQC	Tendency to oligomerize; solubilizing agents needed		
fl	*0.27*	HSQC	Addition of detergent during expression (0.1% MNG-3); stable protein		
**ORF8**					SI19
fl	*0.62*	HSQC	Tendency to oligomerize		
ΔORF8	0.5	Pure protein			
**N protein**					SI20
IDR1-NTD- IDR2	12	NMR assigned	High salt (>0.4 M) for increased stability	50618, 50619, 50558, 50557^d^	
NTD-SR	3	HSQC			
NTD	3	HSQC		34511	
CTD	2	NMR assigned	Stable dimer for >4 months at 4°C and >3 weeks at 30°C	50518^d^	
**ORF9** **b**					SI21
fl	*0.64*	HSQC	Expression without detergent, protein is stable		
**ORF14**					SI22
fl	*0.43*	HSQC	Addition of detergent during expression (0.05% Brij-58); stable in detergent but unstable on lipid reconstitution		
**ORF10**					SI23
fl	2	HSQC	Tendency to oligomerize; unstable upon tag cleavage		

^a^ Yields from bacterial expression represent the minimal protein amount in mg/L independent of the cultivation medium. Italic values indicate yields from CFPS.

^b^ Yields from CFPS represent the minimal protein amount in mg/ml of wheat-germ extract.

^c^ *COVID19-nmr* BMRB depositions yet to be released.

^d^ *COVID19-nmr* BMRB depositions.

We also present protocols for a number of accessory and structural E and M proteins that could only be produced using wheat-germ cell-free protein synthesis (WG-CFPS). In SCoV2, accessory proteins represent a class of mostly small and relatively poorly characterized proteins, mainly due to their difficult behavior in classical expression systems. They are often found in inclusion bodies and difficult to purify in quantities adequate for structural studies. We thus here exploit cell-free synthesis, mainly based on previous reports on production and purification of viral membrane proteins in general (Fogeron et al., 2015b; Fogeron et al., 2017; Jirasko et al., 2020b). Besides yields compatible with structural studies, ribosomes in WG extracts further possess an increased folding capacity (Netzer and Hartl, 1997), favorable for those more complicated proteins.

We exemplify in more detail the optimization of protein production, isotope-labeling, and purification for proteins with different individual challenges: the nucleic acid–binding (NAB) domain of nsp3e, the main protease nsp5, and several auxiliary proteins. For the majority of produced and purified proteins, we achieve >95% purity and provide ^15^N-HSQC spectra as the ultimate quality measure. We also provide additional suggestions for challenging proteins, where our protocols represent a unique resource and starting point exploitable by other labs.

## Materials and Methods

### Strains, Plasmids, and Cloning

The rationale of construct design for all proteins can be found within the respective protocols in Supplementary Tables SI1–SI23. For bacterial production, *E. coli* strains and expression plasmids are given; for WG-CFPS, template vectors are listed. Protein coding sequences of interest have been obtained as either commercial, codon-optimized genes or, for shorter ORFs and additional sequences, annealed from oligonucleotides prior to insertion into the relevant vector. Subcloning of inserts, adjustment of boundaries, and mutations of genes have been carried out by standard molecular biology techniques. All expression plasmids can be obtained upon request from the *COVID19-NMR* consortium (https://covid19-nmr.com/), including information about coding sequences, restriction sites, fusion tags, and vector backbones.

### Protein Production and Purification

For SCoV2 proteins, we primarily used heterologous production in *E*. *coli*. Detailed protocols of individual full-length (fl) proteins, separate domains, combinations, or particular expression constructs as listed in Table 1 can be found in the (Supplementary Tables SI1–SI23).

The ORF3a, ORF6, ORF7b, ORF8, ORF9b, and ORF14 accessory proteins and the structural proteins M and E were produced by WG-CFPS as described in the Supplementary Material. In brief, transcription and translation steps have been performed separately, and detergent has been added for the synthesis of membrane proteins as described previously (Takai et al., 2010; Fogeron et al., 2017).

### NMR Spectroscopy

All amide correlation spectra, either HSQC- or TROSY-based, are representative examples. Details on their acquisition parameters and the raw data are freely accessible through https://covid19-nmr.de or upon request.

## Results

In the following, we provide protocols for the purification of SCoV2 proteins sorted into 1) nonstructural proteins and 2) structural proteins together with accessory ORFs. Table 1 shows an overview of expression constructs. We use a consequent terminology of those constructs, which is guided by domains, intrinsically disordered regions (IDRs) or other particularly relevant sequence features within them. This study uses the SCoV2 NCBI reference genome entry NC_045512.2, identical to GenBank entry MN908947.3 (Wu et al., 2020), unless denoted differently in the respective protocols. Any relevant definition of boundaries can also be found in the SI protocols.

As applicable for a major part of our proteins, we further define a standard procedure for the purification of soluble His-tagged proteins that are obtained through the sequence of **I**MAC, TEV/Ulp1 **P**rotease cleavage, **R**everse IMAC, and **S**ize-exclusion chromatography, eventually with individual alterations, modifications, or additional steps. For convenient reading, we will thus use the abbreviation IPRS to avoid redundant protocol description. Details for every protein, including detailed expression conditions, buffers, incubation times, supplements, storage conditions, yields, and stability, can be found in the respective Supplementary Tables SI1–SI23 (see also Supplementary Tables S1, S2) and Tables 1, 2.

### Nonstructural Proteins

**FIGURE 1:**
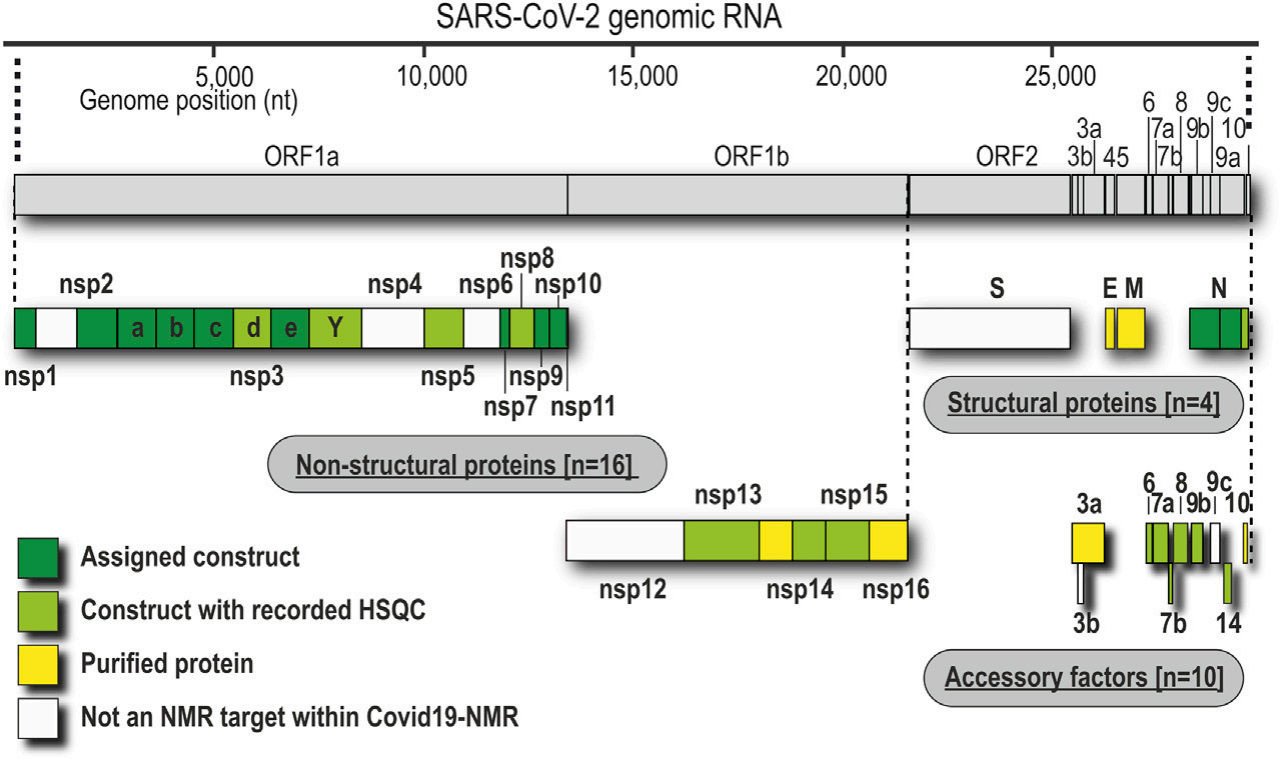
Genomic organization of proteins and current state of analysis or purification. Boxes represent the domain boundaries as outlined in the text and in Table 1. Their position corresponds with the genomic loci. Colors indicate whether the pure proteins were purified (yellow), analyzed by NMR using only HSQC (lime), or characterized in detail, including NMR resonance assignments (green).

We have approached and challenged the recombinant production of a large part of the SCoV2 nsps (Figure 1), with great success (Table 2). We excluded nsp4 and nsp6 (TM proteins), which are little characterized and do not reveal soluble, folded domains by prediction (Oostra et al., 2007; Oostra et al., 2008). The function of the very short (13 aa) nsp11 is unknown, and it seems to be a mere copy of the nsp12 amino-terminal residues, remaining as a protease cleavage product of ORF1a. Further, we left out the RNA-dependent RNA polymerase nsp12 in our initial approach because of its size (>100 kDa) and known unsuitability for heterologous recombinant production in bacteria. Work on NMR-suitable nsp12 bacterial production is ongoing, while other expert labs have succeeded in purifying nsp12 for cryo-EM applications in different systems (Gao et al., 2020; Hillen et al., 2020). For the remainder of nsps, we here provide protocols for fl-proteins or relevant fragments of them.

#### nsp1

nsp1 is the very N-terminus of the polyproteins pp1a and pp1ab and one of the most enigmatic viral proteins, expressed only in α- and β-CoVs (Narayanan et al., 2015). Interestingly, nsp1 displays the highest divergence in sequence and size among different CoVs, justifying it as a genus-specific marker (Snijder et al., 2003). It functions as a host shutoff factor by suppressing innate immune functions and host gene expression (Kamitani et al., 2006; Narayanan et al., 2008; Schubert et al., 2020). This suppression is achieved by an interaction of the nsp1 C-terminus with the mRNA entry tunnel within the 40 S subunit of the ribosome (Schubert et al., 2020; Thoms et al., 2020).

**FIGURE 2:**
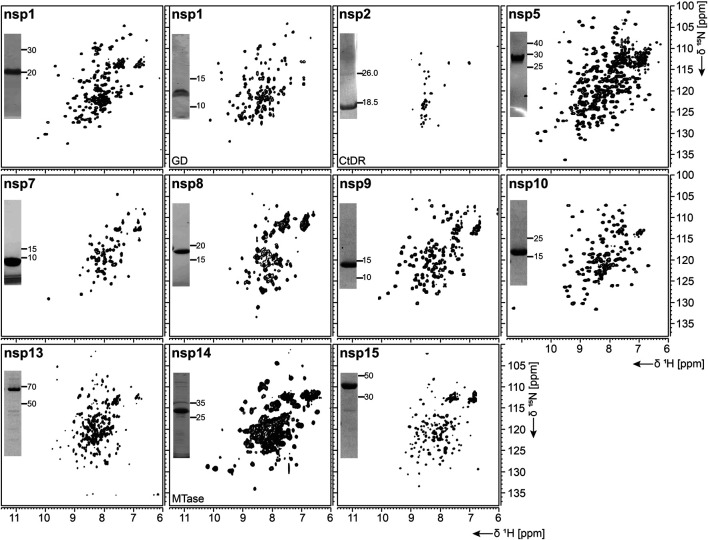
^1^H, ^15^N-correlation spectra of investigated nonstructural proteins. Construct names according to Table 1 are indicated unless fl-proteins are shown. A representative SDS-PAGE lane with final samples is included as inset. Spectra for nsp3 constructs are collectively shown in Figure 3.

As summarized in Table 1, fl-domain boundaries of nsp1 were chosen to contain the first 180 amino acids, in analogy to its closest homolog from SCoV (Snijder et al., 2003). In addition, a shorter construct was designed, encoding only the globular core domain (GD, aa 13–127) suggested by the published SCoV nsp1 NMR structure (Almeida et al., 2007). His-tagged fl nsp1 was purified using the IPRS approach. Protein quality was confirmed by the available HSQC spectrum (Figure 2). Despite the flexible C-terminus, we were able to accomplish a near-complete backbone assignment (Wang et al., 2021).

Interestingly, the nsp1 GD was found to be problematic in our hands despite good expression. We observed insolubility, although buffers were used according to the homolog SCoV nsp1 GD (Almeida et al., 2007). Nevertheless, using a protocol comparable to the one for fl nsp1, we were able to record an HSQC spectrum proving a folded protein (Figure 2).

#### nsp2

nsp2 has been suggested to interact with host factors involved in intracellular signaling (Cornillez-Ty et al., 2009; Davies et al., 2020). The precise function, however, is insufficiently understood. Despite its potential dispensability for viral replication in general, it might be a valuable model to gain insights into virulence due to its possible involvement in the regulation of global RNA synthesis (Graham et al., 2005). We provide here a protocol for the purification of the C-terminal IDR (CtDR) of nsp2 from residues 557 to 601, based on disorder predictions [PrDOS (Ishida and Kinoshita, 2007)]. The His-Trx-tagged peptide was purified by IPRS. Upon dialysis, two IEC steps were performed: first anionic and then cationic, with good final yields (Table 1). Stability and purity were confirmed by an HSQC spectrum (Figure 2) and a complete backbone assignment (Mompean et al., 2020; Table 2).

#### nsp3

nsp3, the largest nsp (Snijder et al., 2003), is composed of a plethora of functionally related, yet independent, subunits. After cleavage of nsp3 from the fl ORF1-encoded polypeptide chain, it displays a 1945-residue multidomain protein, with individual functional entities that are subclassified from nsp3a to nsp3e followed by the ectodomain embedded in two TM regions and the very C-terminal CoV-Y domain. The soluble nsp3a-3e domains are linked by various types of linkers with crucial roles in the viral life cycle and are located in the so-called viral cytoplasm, which is separated from the host cell after budding off the endoplasmic reticulum and contains the viral RNA (Wolff et al., 2020). Remarkably, the nsp3c substructure comprises three subdomains, making nsp3 the most complex SCoV2 protein. The precise function and eventual RNA-binding specificities of nsp3 domains are not yet understood. We here focus on the nsp3 domains a–e and provide elaborated protocols for additional constructs carrying relevant linkers or combinations of domains (Table 1). Moreover, we additionally present a convenient protocol for the purification of the C-terminal CoV-Y domain.

##### nsp3a

**FIGURE 3:**
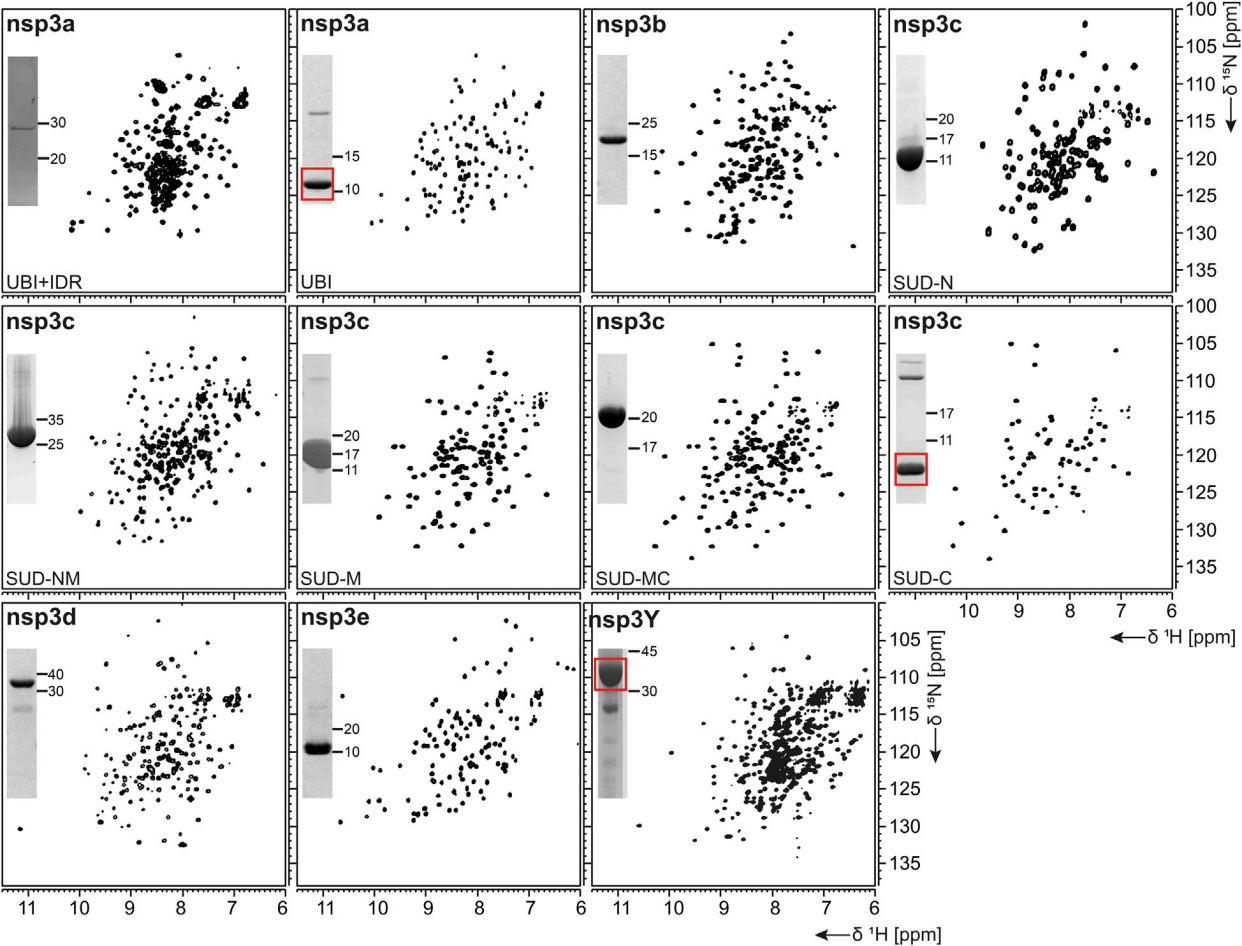
^1^H, ^15^N-correlation spectra of investigated constructs from nonstructural protein 3. Construct names of subdomains according to Table 1 are indicated unless fl-domains are shown. A representative SDS-PAGE lane with final samples is included as inset. Red boxes indicate protein bands of interest.

The N-terminal portion of nsp3 is comprised of a ubiquitin-like (Ubl) structured domain and a subsequent acidic IDR. Besides its ability to bind ssRNA (Serrano et al., 2007), nsp3a has been reported to interact with the nucleocapsid (Hurst et al., 2013; Khan et al., 2020), playing a potential role in virus replication. We here provide protocols for the purification of both the Ubl (aa 1–111) and fl nsp3a (aa 1–206), including the acidic IDR (Ubl + IDR Table 1). Domain boundaries were defined similar to the published NMR structure of SCoV nsp3a (Serrano et al., 2007). His-tagged nsp3a Ubl + IDR and GST-tagged nsp3a Ubl were each purified via the IPRS approach. nsp3a Ubl yielded mM sample concentrations and displayed a well-dispersed HSQC spectrum (Figure 3). Notably, the herein described protocol also enables purification of fl nsp3a (Ubl + IDR) (Tables 1, 2). Despite the unstructured IDR overhang, the excellent protein quality and stability allowed for near-complete backbone assignment [Figure 3, (Salvi et al., 2021)].

##### nsp3b

nsp3b is an ADP-ribose phosphatase macrodomain and potentially plays a key role in viral replication. Moreover, the de-ADP ribosylation function of nsp3b protects SCoV2 from antiviral host immune response, making nsp3b a promising drug target (Frick et al., 2020). As summarized in Table 1, the domain boundaries of the herein investigated nsp3b are residues 207–376 of the nsp3 primary sequence and were identical to available crystal structures with PDB entries 6YWM and 6YWL (unpublished). For purification, we used the IPRS approach, which yielded pure fl nsp3b (Table 2). Fl nsp3b displays well-dispersed HSQC spectra, making this protein an amenable target for NMR structural studies. In fact, we recently reported near-to-complete backbone assignments for nsp3b in its apo and ADP-ribose–bound form (Cantini et al., 2020).

##### nsp3c

The SARS unique domain (SUD) of nsp3c has been described as a distinguishing feature of SCoVs (Snijder et al., 2003). However, similar domains in more distant CoVs, such as MHV or MERS, have been reported recently (Chen et al., 2015; Kusov et al., 2015). nsp3c comprises three distinct globular domains, termed SUD-N, SUD-M, and SUD-C, according to their sequential arrangement: N-terminal (N), middle (M), and C-terminal (C). SUD-N and SUD-M develop a macrodomain fold similar to nsp3b and are described to bind G-quadruplexes (Tan et al., 2009), while SUD-C preferentially binds to purine-containing RNA (Johnson et al., 2010). Domain boundaries for SUD-N and SUD-M and for the tandem-domain SUD-NM were defined in analogy to the SCoV homolog crystal structure (Tan et al., 2009). Those for SUD-C and the tandem SUD-MC were based on NMR solution structures of corresponding SCoV homologs (Table 1) (Johnson et al., 2010). SUD-N, SUD-C, and SUD-NM were purified using GST affinity chromatography, whereas SUD-M and SUD-MC were purified using His affinity chromatography. Removal of the tag was achieved by thrombin cleavage and final samples of all domains were prepared subsequent to size-exclusion chromatography (SEC). Except for SUD-M, all constructs were highly stable (Table 2). Overall protein quality allowed for the assignment of backbone chemical shifts for the three single domains (Gallo et al., 2020) amd good resolved HSQC spectra also for the tandem domains (Figure 3).

##### nsp3d

nsp3d comprises the papain-like protease (PL^pro^) domain of nsp3 and, hence, is one of the two SCoV2 proteases that are responsible for processing the viral polypeptide chain and generating functional proteins (Shin et al., 2020). The domain boundaries of PL^pro^ within nsp3 are set by residues 743 and 1,060 (Table 1). The protein is particularly challenging, as it is prone to misfolding and rapid precipitation. We prepared His-tagged and His-SUMO-tagged PL^pro^. The His-tagged version mainly remained in the insoluble fraction. Still, mg quantities could be purified from the soluble fraction, however, greatly misfolded. Fusion to SUMO significantly enhanced protein yield of soluble PL^pro^. The His-SUMO-tag allowed simple IMAC purification, followed by cleavage with Ulp1 and isolation of cleaved PL^pro^ via a second IMAC. A final purification step using gel filtration led to pure PL^pro^ of both unlabeled and 15N-labeled species (Table 2). The latter has allowed for the acquisition of a promising amide correlation spectrum (Figure 3).

##### nsp3e

**FIGURE 4:**
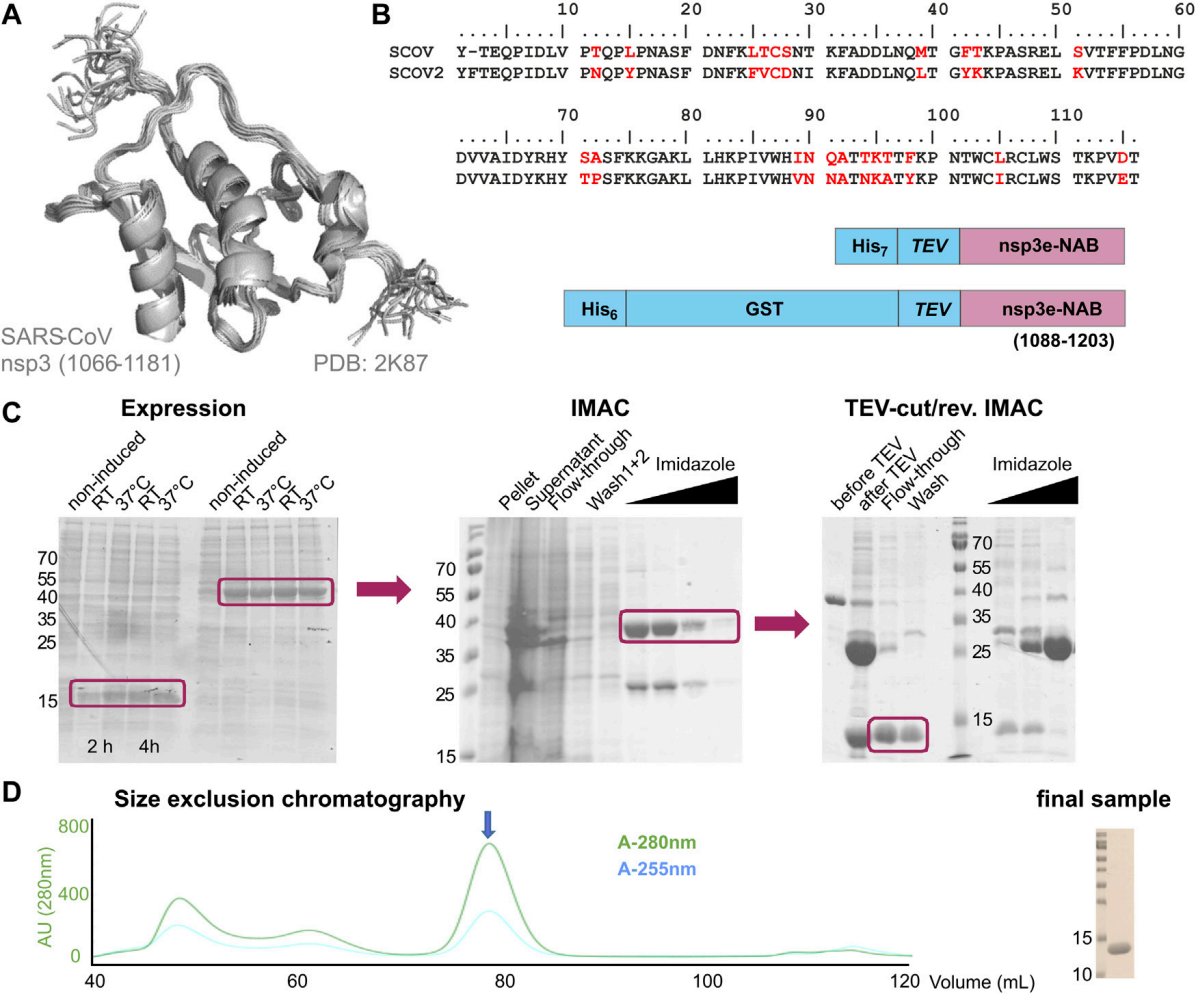
Rationale of construct design, expression, and IPRS purification of the nsp3e nucleic acid–binding domain (NAB). **(A)** NMR structural ensemble of the homologous SCoV nsp3e (Serrano et al., 2009). The domain boundaries as displayed are given. **(B)** Sequence alignment of SCoV and SCoV2 regions representing the nsp3e locus. Arrows indicate the sequence stretch as used for the structure in panel **(A)**. The analogous region was used for the design of the two protein expression constructs shown **(C)**. Left, SDS-PAGE showing the expression of nsp3e constructs from panel **(B)** over 4 h at two different temperatures. Middle, SDS-PAGE showing the subsequent steps of IMAC. Right, SDS-PAGE showing steps and fractions obtained before and after TEV/dialysis and reverse IMAC. Boxes highlight the respective sample species of interest for further usage **(D)** SEC profile of nsp3e following steps in panel **(C)** performed with a Superdex 75 16/600 (GE Healthcare) column in the buffer as denoted in Supplementary Table SI3. The arrow indicates the protein peak of interest containing monomeric and homogenous nsp3e NAB devoid of significant contaminations of nucleic acids as revealed by the excellent 280/260 ratio. Right, SDS-PAGE shows 0.5 µL of the final NMR sample used for the spectrum in Figure 3 after concentrating relevant SEC fractions.

nsp3e is unique to *Betacoronaviruses* and consists of a nucleic acid–binding domain (NAB) and the so-called group 2-specific marker (G2M) (Neuman et al., 2008). Structural information is rare; while the G2M is predicted to be intrinsically disordered (Lei et al., 2018); the only available experimental structure of the nsp3e NAB was solved from SCoV by the Wüthrich lab using solution NMR (Serrano et al., 2009). We here used this structure for a sequence-based alignment to derive reasonable domain boundaries for the SCoV2 nsp3e NAB (Figures 4A,B). The high sequence similarity suggested using nsp3 residues 1,088–1,203 (Table 1). This polypeptide chain was encoded in expression vectors comprising His- and His-GST tags, both cleavable by TEV protease. Both constructs showed excellent expression, suitable for the IPRS protocol (Figure 4C). Finally, a homogenous NAB species, as supported by the final gel of pooled samples (Figure 4D), was obtained. The excellent protein quality and stability are supported by the available HSQC (Figure 3) and a published backbone assignment (Korn et al., 2020a).

##### nsp3Y

nsp3Y is the most C-terminal domain of nsp3 and exists in all coronaviruses (Neuman et al., 2008; Neuman, 2016). Together, though, with its preceding regions G2M, TM 1, the ectodomain, TM2, and the Y1-domain, it has evaded structural investigations so far. The precise function of the CoV-Y domain remains unclear, but, together with the Y1-domain, it might affect binding to nsp4 (Hagemeijer et al., 2014). We were able to produce and purify nsp3Y (CoV-Y) comprising amino acids 1,638–1,945 (Table 1), yielding 12 mg/L with an optimized protocol that keeps the protein in a final NMR buffer containing HEPES and lithium bromide. Although the protein still shows some tendency to aggregate and degrade (Table 2), and despite its relatively large size, the spectral quality is excellent (Figure 3). nsp3 CoV-Y appears suitable for an NMR backbone assignment carried out at lower concentrations in a deuterated background (ongoing).

#### nsp5

**FIGURE 5:**
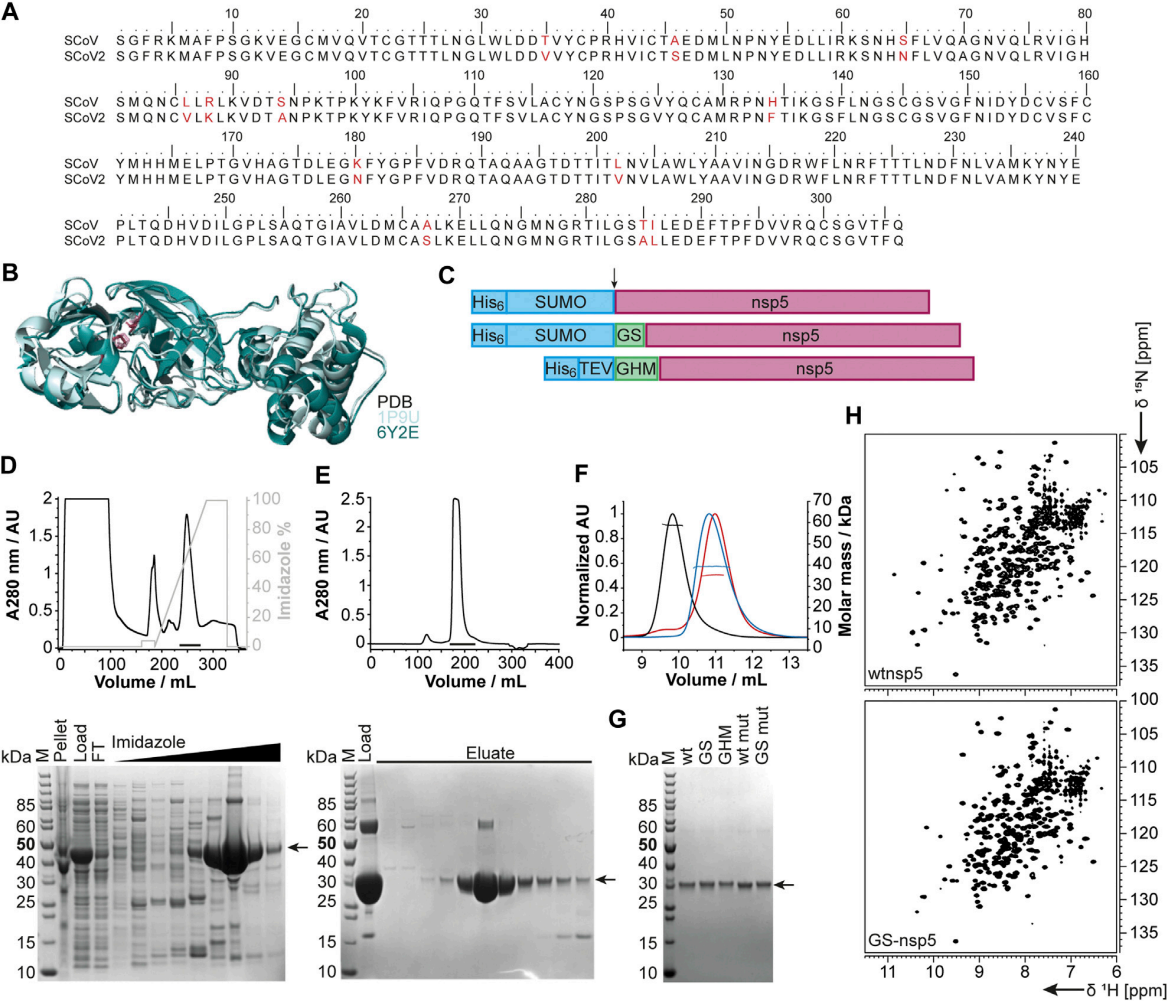
Rationale of construct design, expression, and purification of different nsp5 constructs. **(A)** Sequence alignment of SCoV and SCoV2 fl nsp5. **(B)** X-ray structural overlay of the homologous SCoV (PDB 1P9U, light blue) and SCoV2 nsp5 (PDB 6Y2E, green) in cartoon representation. The catalytic dyad (H41 and C145) is shown in stick representation (magenta). **(C)** Schematics of nsp5 expression constructs involving purification and solubilization tags (blue), different N-termini and additional aa after cleavage (green), and nsp5 (magenta). Cleavage sites are indicated by an arrow. **(D, E)** An exemplary purification is shown for wtnsp5. IMAC **(D)** and SEC **(E)** chromatograms (upper panels) and the corresponding SDS PAGE (lower panels). Black bars in the chromatograms indicate pooled fractions. Gel samples are as follows: M: MW standard; pellet/load: pellet/supernatant after cell lysis; FT: IMAC flow-through; imidazole: eluted fractions with linear imidazole gradient; eluate: eluted SEC fractions from input (load). **(F)** SEC-MALS analysis with ∼0.5 µg of wtnsp5 without additional aa (wtnsp5, black) with GS (GS-nsp5, blue) and with GHM (GHM-nsp5, red)) in NMR buffer on a Superdex 75, 10/300 GL (GE Healthcare) column. Horizontal lines indicate fractions of monodisperse nsp5 used for MW determination. **(G)** A SDS-PAGE showing all purified nsp5 constructs. The arrow indicates nsp5. **(H)** Exemplary [^15^N, ^1^H]-BEST-TROSY spectra measured at 298 K for the dimeric wtnsp5 (upper spectrum) and monomeric GS-nsp5 (lower spectrum). See Supplementary Table SI4 for technical details regarding this figure.

The functional main protease nsp5 (M^pro^) is a dimeric cysteine protease (Ullrich and Nitsche, 2020). Amino acid sequence and 3D structure of SCoV [PDB 1P9U (Anand et al., 2003)] and SCoV2 (PDB 6Y2E [Zhang et al., 2020)] homologs are highly conserved (Figures 5A,B). The dimer interface involves the N-termini of both monomers, which puts considerable constraints on the choice of protein sequence for construct design regarding the N-terminus.

We thus designed different constructs differing in the N-terminus: the native N-terminus (wt), a GS mutant with the additional N-terminal residues glycine and serine as His-SUMO fusion, and a GHM mutant with the amino acids glycine, histidine, and methionine located at the N-terminus with His-tag and TEV cleavage site (Figure 5C). Purification of all proteins via the IPRS approach (Figures 5D,E) yielded homogenous and highly pure protein, analyzed by PAGE (Figure 5G), mass spectrometry, and 2D [^15^N, ^1^H]-BEST TROSY spectra (Figure 5H). Final yields are summarized in Table 2.

#### nsp7 and nsp8

Both nsp7 and nsp8 are auxiliary factors of the polymerase complex together with the RNA-dependent RNA polymerase nsp12 and have high sequence homology with SCoV (100% and 99%, respectively) (Gordon et al., 2020). For nsp7 in complex with nsp8 or for nsp8 alone, additional functions in RNA synthesis priming have been proposed (Tvarogova et al., 2019; Konkolova et al., 2020). In a recent study including an RNA-substrate-bound structure (Hillen et al., 2020), both proteins (with two molecules of nsp8 and one molecule of nsp7 for each nsp12 RNA polymerase) were found to be essential for polymerase activity in SCoV2. For both fl-proteins, a previously established expression and IPRS purification strategy for the SCoV proteins (Kirchdoerfer and Ward, 2019) was successfully transferred, which resulted in decent yields of reasonably stable proteins (Table 2). Driven by its intrinsically oligomeric state, nsp8 showed some tendency toward aggregation, limiting the available sample concentration. The higher apparent molecular weight and limited solubility are also reflected in the success of NMR experiments. While we succeeded in a complete NMR backbone assignment of nsp7 (Tonelli et al., 2020), the quality of the spectra obtained for nsp8 is currently limited to the HSQC presented in Figure 2.

#### nsp9

The 12.4 kDa ssRNA-binding nsp9 is highly conserved among *Betacoronaviruses*. It is a crucial part of the viral replication machinery (Miknis et al., 2009), possibly targeting the 3’-end stem-loop II (s2m) of the genome (Robertson et al., 2005). nsp9 adopts a fold similar to oligonucleotide/oligosaccharide-binding proteins (Egloff et al., 2004), and structural data consistently uncovered nsp9 to be dimeric in solution (Egloff et al., 2004; Sutton et al., 2004; Miknis et al., 2009; Littler et al., 2020). Dimer formation seems to be a prerequisite for viral replication (Miknis et al., 2009) and influences RNA-binding (Sutton et al., 2004), despite a moderate affinity for RNA *in vitro* (Littler et al., 2020).

Based on the early available crystal structure of SCoV2 nsp9 (PDB 6W4B, unpublished), we used the 113 aa fl sequence of nsp9 for our expression construct (Table 1). Production of either His- or His-GST-tagged fl nsp9 yielded high amounts of soluble protein in both natural abundance and ^13^C- and ^15^N-labeled form. Purification *via* the IPRS approach enabled us to separate fl nsp9 in different oligomer states. The earliest eluted fraction represented higher oligomers, was contaminated with nucleic acids and was not possible to concentrate above 2 mg/ml. This was different for the subsequently eluting dimeric fl nsp9 fraction, which had a A260/280 ratio of below 0.7 and could be concentrated to >5 mg/ml (Table 2). The excellent protein quality and stability are supported by the available HSQC (Figure 2), and a near-complete backbone assignment (Dudas et al., 2021).

#### nsp10

The last functional protein encoded by ORF1a, nsp10, is an auxiliary factor for both the methyltransferase/exonuclease nsp14 and the 2′-O-methyltransferase (MTase) nsp16. However, it is required for the MTase activity of nsp16 (Krafcikova et al., 2020), it confers exonuclease activity to nsp14 in the RNA polymerase complex in SCoV (Ma et al., 2015). It contains two unusual zinc finger motifs (Joseph et al., 2006) and was initially proposed to comprise RNA-binding properties. We generated a construct (Table 1) containing an expression and affinity purification tag on the N-terminus as reported for the SCoV variant (Joseph et al., 2006). Importantly, additional Zn^2+^ ions present during expression and purification stabilize the protein significantly (Kubatova et al., 2020). The yield during isotope-labeling was high (Table 2), and tests in unlabeled rich medium showed the potential for yields exceeding 100 mg/L. These characteristics facilitated in-depth NMR analysis and a backbone assignment (Kubatova et al., 2020).

#### nsp13

nsp13 is a conserved ATP-dependent helicase that has been characterized as part of the RNA synthesis machinery by binding to nsp12 (Chen et al., 2020b). It represents an interesting drug target, for which the available structure (PDB 6ZSL) serves as an excellent basis (Table 1). The precise molecular function, however, has remained enigmatic since it is not clear whether the RNA unwinding function is required for making ssRNA accessible for RNA synthesis (Jia et al., 2019) or whether it is required for proofreading and backtracking (Chen et al., 2020b). We obtained pure protein using a standard expression vector, generating a His-SUMO-tagged protein. Following Ulp1 cleavage, the protein showed limited protein stability in the solution (Table 2).

#### nsp14

nsp14 contains two domains: an N-terminal exonuclease domain and a C-terminal MTase domain (Ma et al., 2015). The exonuclease domain interacts with nsp10 and provides part of the proofreading function that supports the high fidelity of the RNA polymerase complex (Robson et al., 2020). Several unusual features, such as the unusual zinc finger motifs, set it apart from other DEDD-type exonucleases (Chen et al., 2007), which are related to both nsp10 binding and catalytic activity. The MTase domain modifies the N7 of the guanosine cap of genomic and subgenomic viral RNAs, which is essential for the translation of viral proteins (Thoms et al., 2020). The location of this enzymatic activity within the RNA synthesis machinery ensures that newly synthesized RNA is rapidly capped and thus stabilized. As a strategy, we used constructs, which allow coexpression of both nsp14 and nsp10 (pRSFDuet and pETDuet, respectively). Production of isolated fl nsp14 was successful, however, with limited yield and stability (Table 2). Expression of the isolated MTase domain resulted in soluble protein with 27.5 kDa mass that was amenable to NMR characterization (Figure 2), although only under reducing conditions and in the presence of high (0.4 M) salt concentration.

#### nsp15

The poly-U-specific endoribonuclease nsp15 was one of the very first SCoV2 structures deposited in the PDB [6VWW, (Kim et al., 2020)]. Its function has been suggested to be related to the removal of U-rich RNA elements, preventing recognition by the innate immune system (Deng et al., 2017), even though the precise mechanism remains to be established. The exact role of the three domains (N-terminal, middle, and C-terminal catalytic domain) also remains to be characterized in more detail (Kim et al., 2020). Here, the sufficient yield of fl nsp15 during expression supported purification of pure protein, which, however, showed limited stability in solution (Table 2).

#### nsp16

The MTase reaction catalyzed by nsp16 is dependent on nsp10 as a cofactor (Krafcikova et al., 2020). In this reaction, the 2’-OH group of nucleotide +1 in genomic and subgenomic viral RNA is methylated, preventing recognition by the innate immune system. Since both nsp14 and nsp16 are in principle susceptible to inhibition by MTase inhibitors, a drug targeting both enzymes would be highly desirable (Bouvet et al., 2010). nsp16 is the last protein being encoded by ORF1ab, and only its N-terminus is formed by cleavage by the M^pro^ nsp5. Employing a similar strategy to that for nsp14, nsp16 constructs were designed with the possibility of nsp10 coexpression. Expression of fl nsp16 resulted in good yields, when expressed both isolated and together with nsp10. The protein, however, is in either case unstable in solution and highly dependent on reducing buffer conditions (Table 2). The purification procedures of nsp16 were adapted with minor modifications from a previous X-ray crystallography study (Rosas-Lemus et al., 2020).

### Structural Proteins and Accessory ORFs

Besides establishing expression and purification protocols for the nsps, we also developed protocols and obtained pure mg quantities of the SCoV2 structural proteins E, M, and N, as well as literally all accessory proteins. With the exception of the relatively well-behaved nucleocapsid (N) protein, SCoV2 E, M, and the remaining accessory proteins represent a class of mostly small and relatively poorly characterized proteins, mainly due to their difficult behavior in classical expression systems.

**FIGURE 6:**
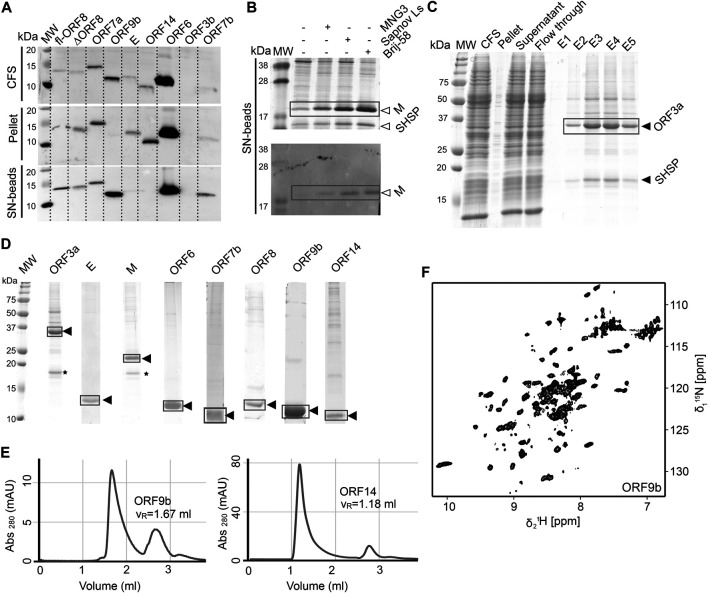
Cell-free protein synthesis of accessory ORFs and structural proteins E and M. **(A)** Screening for expression and solubility of different ORFs using small-scale reactions. The total cell-free reaction (CFS), the pellet after centrifugation, and the supernatant (SN) captured on magnetic beads coated with Strep-Tactin were analyzed. All tested proteins were synthesized, with the exception of ORF3b. MW, MW standard. **(B)** Detergent solubilization tests using three different detergents, here at the example of the M protein, shown by SDS-PAGE and Western Blot. **(C)** Proteins are purified in a single step using a Strep-Tactin column. For ORF3a (and also for M), a small heat-shock protein of the HSP20 family is copurified, as identified by mass spectrometry (see also * in Panel **D**). **(D)** SDS-PAGE of the ^2^H, ^13^C, ^15^N-labeled proteins used as NMR samples. Yields were between 0.2 and 1 mg protein per mL wheat-germ extract used. **(E)** SEC profiles for two ORFs. Left, ORF9b migrates as expected for a dimer. Right, OFR14 shows large assemblies corresponding to approximately 9 protein units and the DDM detergent micelle. **(F)** 2D [^15^N, ^1^H]-BEST-TROSY spectrum of ORF9b, recorded at 900 MHz in 1 h at 298 K, on less than 1 mg of protein. See Supplementary Tables SI13–SI19 and Supplementary Tables SI19, SI20 for technical and experimental details regarding this figure.

We used wheat-germ cell-free protein synthesis (WG-CFPS) for the successful production, solubilization, purification, and, in part, initial NMR spectroscopic investigation of ORF3a, ORF6, ORF7b, ORF8, ORF9b, and ORF14 accessory proteins, as well as E and M in mg quantities using the highly efficient translation machinery extracted from wheat-germs (Figures 6A–D).

#### ORF3a

The protein from ORF3a in SCoV2 corresponds to the accessory protein 3a in SCoV, with homology of more than 70% (Table 1). It has 275 amino acids, and its structure has recently been determined (Kern et al., 2020). The structure of SCoV2 3a displays a dimer, but it can also form higher oligomers. Each monomer has three TM helices and a cytosolic β-strand rich domain. SCoV2 ORF3a is a cation channel, and its structure has been solved by electron microscopy in nanodiscs. In SCoV, 3a is a structural component and was found in recombinant virus-like particles (Liu et al., 2014), but is not explicitly needed for their formation. The major challenge for NMR studies of this largest accessory protein is its size, independent of its employment in solid state or solution NMR spectroscopy.

As most other accessory proteins described in the following, ORF3a has been produced using WG-CFPS and was expressed in soluble form in the presence of Brij-58 (Figure 6C). It is copurified with a small heat-shock protein of the HSP20 family from the wheat-germ extract. The protocol described here is highly similar to that of the other cell-free synthesized accessory proteins. Where NMR spectra have been reported, the protein has been produced in a ^2^H, ^13^C, ^15^N uniformly labeled form; otherwise, natural abundance amino acids were added to the reaction. The proteins were further affinity-purified in one step using Strep-Tactin resin, through the Strep-tag II fused to their N- or C-terminus. For membrane proteins, protein synthesis and also purification were done in the presence of detergent.

About half a milligram of pure protein was generally obtained per mL of extract, and up to 3 ml wheat-germ extract have been used to prepare NMR samples.

#### ORF3b

The ORF3b protein is a putative protein stemming from a short ORF (57 aa) with no homology to existing SCoV proteins (Chan et al., 2020). Indeed, ORF3b gene products of SCoV2 and SCoV are considerably different, with one of the distinguishing features being the presence of premature stop codons, resulting in the expression of a drastically shortened ORF3b protein (Konno et al., 2020). However, the SCoV2 nucleotide sequence after the stop codon shows a high similarity to the SCoV ORF3b. Different C-terminal truncations seem to play a role in the interferon-antagonistic activity of ORF3b (Konno et al., 2020). ORF3b is the only protein that, using WG-CFPS, was not synthesized at all; i.e., it was neither observed in the total cell-free reaction nor in supernatant or pellet. This might be due to the premature stop codon, which was not considered. Constructs of ORF3b thus need to be redesigned.

#### ORF4 (Envelope Protein, E)

The SCoV2 envelope (E) protein is a small (75 amino acids), integral membrane protein involved in several aspects of the virus’ life cycle, such as assembly, budding, envelope formation, and pathogenicity, as recently reviewed in (Schoeman and Fielding, 2020). Structural models for SCoV (Surya et al., 2018) and the TM helix of SCoV2 (Mandala et al., 2020) E have been established. The structural models show a pentamer with a TM helix. The C-terminal part is polar, with charged residues interleaved, and is positioned on the membrane surface in SCoV. E was produced in a similar manner to ORF3a, using the addition of detergent to the cell-free reaction.

#### ORF5 (Membrane Glycoprotein, M)

The M protein is the most abundant protein in the viral envelope and is believed to be responsible for maintaining the virion in its characteristic shape (Huang et al., 2004). M is a glycoprotein and sequence analyses predict three domains: A C-terminal endodomain, a TM domain with three predicted helices, and a short N-terminal ectodomain. M is essential for viral particle assembly. Intermolecular interactions with the other structural proteins, N and S to a lesser extent, but most importantly E (Vennema et al., 1996), seem to be central for virion envelope formation in coronaviruses, as M alone is not sufficient. Evidence has been presented that M could adopt two conformations, elongated and compact, and that the two forms fulfill different functions (Neuman et al., 2011). The lack of more detailed structural information is in part due to its small size, close association with the viral envelope, and a tendency to form insoluble aggregates when perturbed (Neuman et al., 2011). The M protein is readily produced using cell-free synthesis in the presence of detergent; as ORF3a, it is copurified with a small heat-shock protein of the HSP20 family (Figure 6B). Membrane-reconstitution will likely be necessary to study this protein.

#### ORF6

The ORF6 protein is incorporated into viral particles and is also released from cells (Huang et al., 2004). It is a small protein (61 aa), which has been found to concentrate at the endoplasmic reticulum and Golgi apparatus. In a murine coronavirus model, it was shown that expressing ORF6 increased virulence in mice (Zhao et al., 2009), and results indicate that ORF6 may serve an important role in the pathogenesis during SCoV infection (Liu et al., 2014). Also, it showed to inhibit the expression of certain STAT1-genes critical for the host immune response and could contribute to the immune evasion. ORF6 is expressed very well in WG-CFPS; the protein was fully soluble with detergents and partially soluble without them and was easily purified in the presence of detergent, but less efficiently in the absence thereof. Solution NMR spectra in the presence of detergent display narrow but few resonances, which correspond, in addition to the C-terminal STREP-tag, to the very C-terminal ORF6 protein residues.

#### ORF7a

SCoV2 protein 7a (121 aa) shows over 85% homology with the SCoV protein 7a. While the SCoV2 7a protein is produced and retained intracellularly, SCoV protein 7a has also been shown to be a structural protein incorporated into mature virions (Liu et al., 2014). 7a is one of the accessory proteins, of which a (partial) structure has been determined at high resolution for SCoV2 (PDB 6W37). However, the very N-terminal signal peptide and the C-terminal membrane anchor, both highly hydrophobic, have not been determined experimentally yet.

**FIGURE 7:**
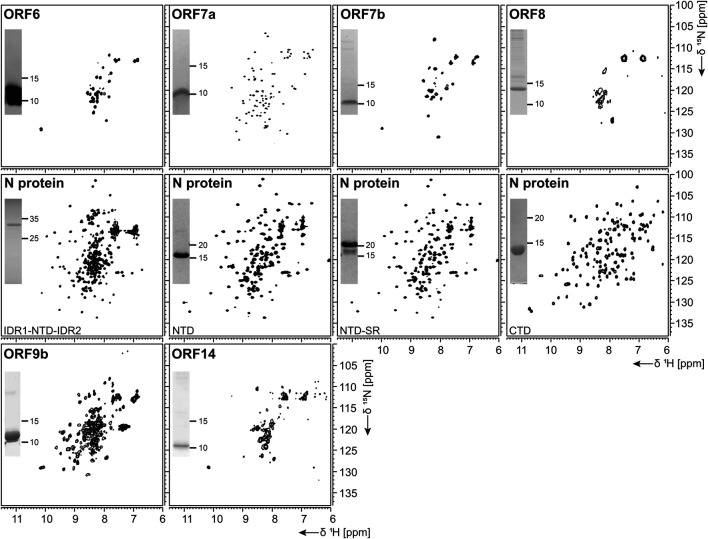
^1^H, ^15^N-correlation spectra of investigated structural and accessory proteins. Construct names according to Table 1 are indicated unless fl-proteins are shown. A representative SDS-PAGE lane with final samples is included as inset.

Expression of the ORF7a ectodomain (ED) with a GB1 tag (Bogomolovas et al., 2009) was expected to produce reasonable yields. The IPRS purification resulted in a highly stable protein, as evidenced by the NMR data obtained (Figure 7).

#### ORF7b

Protein ORF7b is associated with viral particles in a SARS context (Liu et al., 2014). Protein 7b is one of the shortest ORFs with 43 residues. It shows a long hydrophobic stretch, which might correspond to a TM segment. It shows over 93% sequence homology with a bat coronavirus 7b protein (Liu et al., 2014). There, the cysteine residue in the C-terminal part is not conserved, which might facilitate structural studies. ORF7b has been synthesized successfully both from bacteria and by WG-CFPS in the presence of detergent and could be purified using a STREP-tag (Table 2). Due to the necessity of solubilizing agent and its obvious tendency to oligomerize, structure determination, fragment screening, and interaction studies are challenging. However, we were able to record the first promising HSQC, as shown in Figure 7.

#### ORF8

ORF 8 is believed to be responsible for the evolution of *Betacoronaviruses* and their species jumps (Wu et al., 2016) and to have a role in repressing the host response (Tan et al., 2020). ORF 8 (121 aa) from SCoV2 does not apparently exist in SCoV on the protein level, despite the existence of a putative ORF. The sequences of the two homologs only show limited identity, with the exception of a small 7 aa segment, where, in SCoV, the glutamate is replaced with an aspartate. It, however, aligns very well with several coronaviruses endemic to animals, including Paguma and Bat (Chan et al., 2020). The protein comprises a hydrophobic peptide at its very N-terminus, likely corresponding to a signal peptide; the remaining part does not show any specific sequence features. Its structure has been determined (PDB 7JTL) and shows a similar fold to ORF7a (Flower et al., 2020). In this study, ORF8 has been used both with (fl) and without signal peptide (ΔORF8). We first tested the production of ORF8 in *E. coli*, but yields were low because of insolubility. Both ORF8 versions have then been synthesized in the cell-free system and were soluble in the presence of detergent. Solution NMR spectra, however, indicate that the protein is forming either oligomers or aggregates.

#### ORF9a (Nucleocapsid Protein, N)

The nucleocapsid protein (N) is important for viral genome packaging (Luo et al., 2006). The multifunctional RNA-binding protein plays a crucial role in the viral life cycle (Chang et al., 2014) and its domain architecture is highly conserved among coronaviruses. It comprises the N-terminal intrinsically disordered region (IDR1), the N-terminal RNA-binding globular domain (NTD), a central serine/arginine- (SR-) rich intrinsically disordered linker region (IDR2), the C-terminal dimerization domain (CTD), and a C-terminal intrinsically disordered region (IDR3) (Kang et al., 2020).

N represents a highly promising drug target. We thus focused our efforts not exclusively on the NTD and CTD alone, but, in addition, also provide protocols for IDR-containing constructs within the N-terminal part.

##### N-Terminal Domain

The NTD is the RNA-binding domain of the nucleocapsid (Kang et al., 2020). It is embedded within IDRs, functions of which have not yet been deciphered. Recent experimental and bioinformatic data indicate involvement in liquid-liquid phase separation (Chen et al., 2020a).

For the NTD, several constructs were designed, also considering the flanking IDRs (Table 1). In analogy to the available NMR [PDB 6YI3, (Dinesh et al., 2020)] and crystal [PDB 6M3M, (Kang et al., 2020)] structures of the SCoV2 NTD, boundaries for the NTD and the NTD-SR domains were designed to span residues 44–180 and 44–212, respectively. In addition, an extended IDR1-NTD-IDR2 (residues 1–248) construct was designed, including the N-terminal disordered region (IDR1), the NTD domain, and the central disordered linker (IDR2) that comprises the SR region. His-tagged NTD and NTD-SR were purified using IPRS and yielded approx. 3 mg/L in ^15^N-labeled minimal medium. High protein quality and stability are supported by the available HSQC spectra (Figure 7).

The untagged IDR1-NTD-IDR2 was purified by IEC and yielded high amounts of ^13^C, ^15^N-labeled samples of 12 mg/L for further NMR investigations. The quality of our purification is confirmed by the available HSQC (Figure 7), and a near-complete backbone assignment of the two IDRs was achieved (Guseva et al., 2021; Schiavina et al., 2021). Notably, despite the structurally and dynamically heterogeneous nature of the N protein, the mentioned N constructs revealed a very good long-term stability, as shown in Table 2.

##### C-Terminal Domain

Multiple studies on the SCoV2 CTD, including recent crystal structures (Ye et al., 2020; Zhou et al., 2020), confirm the domain as dimeric. Its ability to self-associate seems to be necessary for viral replication and transcription (Luo et al., 2006). In addition, the CTD was shown to, presumably nonspecifically, bind ssRNA (Zhou et al., 2020).

Domain boundaries for the CTD were defined to comprise amino acids 247–364 (Table 1), in analogy to the NMR structure of the CTD from SCoV (PDB 2JW8, [Takeda et al., 2008)]. Gene expression of His- or His-GST-tagged CTD yielded high amounts of soluble protein. Purification was achieved via IPRS. The CTD eluted as a dimer judged by its retention volume on the size-exclusion column and yielded good amounts (Table 2). The excellent protein quality and stability are supported by the available HSQC spectrum (Figure 7) and a near-complete backbone assignment (Korn et al., 2020b).

#### ORF9b

Protein 9b (97 aa) shows 73% sequence homology to the SCoV and also to bat virus (bat-SL-CoVZXC21) 9b protein (Chan et al., 2020). The structure of SCoV2 ORF9b has been determined at high resolution (PDB 6Z4U). Still, a significant portion of the structure was not found to be well ordered. The protein shows a β-sheet-rich structure and a hydrophobic tunnel, in which bound lipid was identified. How this might relate to membrane binding is not fully understood at this point. The differences in sequence between SCoV and SCoV2 are mainly located in the very N-terminus, which was not resolved in the structure (PDB 6Z4U). Another spot of deviating sequence not resolved in the structure is a solvent-exposed loop, which presents a potential interacting segment. ORF9b has been synthesized as a dimer (Figure 6E) using WG-CFPS in its soluble form. Spectra show a well-folded protein, and assignments are underway (Figure 6F).

#### ORF14 (ORF9c)

ORF14 (73 aa) remains, at this point in time, hypothetical. It shows 89% homology with a bat virus protein (bat-SL-CoVZXC21). It shows a highly hydrophobic part in its C-terminal region, comprising two negatively charged residues and a charged/polar N-terminus. The C-terminus is likely mediating membrane interaction. While ORF14 has been synthesized in the wheat-germ cell-free system in the presence of detergent and solution NMR spectra have been recorded, they hint at an aggregated protein (Figure 6E). Membrane-reconstitution of ORF14 revealed an unstable protein, which had been degraded during detergent removal.

#### ORF10

The ORF10 protein is comprised of 38 aa and is a hypothetical protein with unknown function (Yoshimoto, 2020). SCoV2 ORF10 displays 52.4% homology to SCoV ORF9b. The protein sequence is rich in hydrophobic residues, rendering expression and purification challenging. Expression of ORF10 as His-Trx-tagged or His-SUMO tagged fusion protein was possible; however, the ORF10 protein is poorly soluble and shows partial unfolding, even as an uncleaved fusion protein. Analytical SEC hints at oligomerization under the current conditions.

## Discussion

The ongoing SCoV2 pandemic and its manifestation as the COVID-19 disease call for an urgent provision of therapeutics that will specifically target viral proteins and their interactions with each other and RNAs, which are crucial for viral propagation. Two “classical” viral targets have been addressed in comprehensive approaches soon after the outbreak in December 2019: the viral protease nsp5 and the RNA-dependent RNA polymerase (RdRp) nsp12. While the latter turned out to be a suitable target using the repurposed compound Remdesivir (Hillen et al., 2020), nsp5 is undergoing a broad structure-based screen against a battery of inhibitors in multiple places (Jin et al., 2020; Zhang et al., 2020), but with, as of yet, the limited outcome for effective medication. Hence, a comprehensive, reliable treatment of COVID-19 at any stage after the infection has remained unsuccessful.

Further viral protein targets will have to be taken into account in order to provide inhibitors with increased specificity and efficacy and preparative starting points for following potential generations of (SARS-)CoVs. Availability of those proteins in a recombinant, pure, homogenous, and stable form in milligrams is, therefore, a prerequisite for follow-up applications like vaccination, high-throughput screening campaigns, structure determination, and mapping of viral protein interaction networks. We here present, for the first time, a near-complete compendium of SCoV2 protein purification protocols that enable the production of large amounts of pure proteins.

The *COVID19-NMR* consortium was launched with the motivation of providing NMR assignments of all SCoV2 proteins and RNA elements, and enormous progress has been made since the outbreak of COVID-19 for both components [see Table 2 and (Wacker et al., 2020)]. Consequently, we have put our focus on producing proteins in stable isotope-labeled forms for NMR-based applications, e.g., the site-resolved mapping of interactions with compounds (Li and Kang, 2020). Relevant to a broad scientific community, we here report our protocols to suite perfectly any downstream biochemical or biomedical application.

### Overall Success and Protein Coverage

As summarized in Table 2, we have successfully purified 80% of the SCoV2 proteins either in fl or providing relevant fragments of the parent protein. Those include most of the nsps, where all of the known/predicted soluble domains have been addressed (Figure 1). For a very large part, we were able to obtain protein samples of high purity, homogeneity, and fold for NMR-based applications. We would like to point out a number of CoV proteins that, evidenced by their HSQCs, for the first time, provide access to structural information, e.g., the PL^pro^ nsp3d and nsp3Y. Particularly for the nsp3 multidomain protein, we here present soluble samples of almost the complete cytosolic region with more than 120 kDa in the form of excellent 2D NMR spectra (Figure 3), a major part of which fully backbone-assigned. We thus enable the exploitation of the largest and most enigmatic multifunctional SCoV2 protein through individual domains in solution, allowing us to study their concerted behavior with single residue resolution. Similarly, for nsp2, we provide a promising starting point for studying the so far neglected, often uncharacterized, and apparently unstructured proteins.

Driven by the fast-spreading COVID-19, we initially left out proteins that require advanced purification procedures (e.g., nsp12 and S) or where *a priori* information was limited (nsp4 and nsp6). This procedure seems justified with the time-saving approach of our effort in favor of the less attended proteins. However, we are in the process of collecting protocols for the missing proteins.

### Different Complexities and Challenges

The compilation of protein production protocols, initially guided by information from CoV homologs (Table 1), has confronted us with very different levels of complexity. With some prior expectation toward this, we have shared forces to quickly “work off” the highly conserved soluble and small proteins and soon put focus into the processing of the challenging ones. The difficulties in studying this second class of proteins are due to their limited sequence conservation, no prior information, large molecular weights, insolubility, and so forth.

The nsp3e NAB represents one example where the available NMR structure of the SCoV homolog provided a *bona fide* template for selecting initial domain boundaries (Figure 4). The transfer of information derived from SCoV was straightforward; the transferability included the available protocol for the production of comparable protein amounts and quality, given the high sequence identity. In such cases, we found ourselves merely to adapt protocols and optimize yields based on slightly different expression vectors and *E. coli* strains.

However, in some cases, such transfer was unexpectedly not successful, e.g., for the short nsp1 GD. Despite intuitive domain boundaries with complete local sequence identity seen from the SCoV nsp1 NMR structure, it took considerable efforts to purify an analogous nsp1 construct, which is likely related to the impaired stability and solubility caused by a number of impacting amino acid exchanges within the domain’s flexible loops. In line with that, currently available structures of SCoV2 nsp1 have been obtained by crystallography or cryo-EM and include different buffers. As such, our initial design was insufficient in terms of taking into account the parameters mentioned above. However, one needs to consider those particular differences between the nsp1 homologs as one of the most promising target sites for potential drugs as they appear to be hotspots in the CoV evolution and will have essential effects for the molecular networks, both in the virus and with the host (Zust et al., 2007; Narayanan et al., 2015; Shen et al., 2019; Thoms et al., 2020).

A special focus was put on the production of the SCoV2 main protease nsp5, for which NMR-based screenings are ongoing. The main protease is critical in terms of inhibitor design as it appears under constant selection, and novel mutants remarkably influence the structure and biochemistry of the protein (Cross et al., 2020). In the present study, the expression of the different constructs allowed us to characterize the protein in both its monomeric and dimeric forms. Comparison of NMR spectra reveals that the constructs with additional amino acids (GS and GHM mutant) display marked structural differences to the wild-type protein while being structurally similar among themselves (Figure 5H). The addition of two residues (GS) interferes with the dimerization interface, despite being similar to its native N-terminal amino acids (SGFR). We also introduced an active site mutation that replaces cysteine 145 with alanine (Hsu et al., 2005). Intriguingly, this active site mutation C145A, known to stabilize the dimerization of the main protease (Chang et al., 2007), supports dimer formation of the GS added construct (GS-nsp5 C145A) shown by its 2D NMR spectrum overlaying with the one of wild-type nsp5 (Supplementary Table SI4). The NMR results are in line with SEC-MALS analyses (Figure 5F). Indeed, the additional amino acids at the N-terminus shift the dimerization equilibrium toward the monomer, whereas the mutation shifts it toward the dimer despite the N-terminal aa additions. This example underlines the need for a thorough and precise construct design and the detailed biochemical and NMR-based characterization of the final sample state. The presence of monomers vs. dimers will play an essential role in the inhibitor search against SCoV2 proteins, as exemplified by the particularly attractive nsp5 main protease target.

### Exploiting Nonbacterial Expression

As a particular effort within this consortium, we included the so far neglected accessory proteins using a structural genomics procedure supported by wheat-germ cell-free protein synthesis. This approach allowed us previously to express a variety of difficult viral proteins in our hands (Fogeron et al., 2015a; Fogeron et al., 2015b; Fogeron et al., 2016; Fogeron et al., 2017; Wang et al., 2019; Jirasko et al., 2020a). Within the workflow, we especially highlight the straightforward solubilization of the membrane proteins through the addition of detergent to the cell-free reaction, which allowed the production of soluble protein in milligram amounts compatible with NMR studies. While home-made extracts were used here, very similar extracts are available commercially (Cell-Free Sciences, Japan) and can thus be implemented by any lab without prior experience. Also, a major benefit of the WG-CFPS system for NMR studies lies in the high efficiency and selectivity of isotopic labeling. In contrast to cell-based expression systems, only the protein of interest is produced (Morita et al., 2003), which allows bypassing extensive purification steps. In fact, one-step affinity purification is in most cases sufficient, as shown for the different ORFs in this study. Samples could be produced for virtually all proteins, with the exception of the ORF3b construct used. With new recent insight into the stop codons present in this ORF, constructs will be adapted, which shall overcome the problems of ORF3b production (Konno et al., 2020).

For two ORFs, 7b and 8, we exploited a paralleled production strategy, i.e., both in bacteria and via cell-free synthesis. For those challenging proteins, we were, in principle, able to obtain pure samples from either expression system. However, for ORF7b, we found a strict dependency on detergents for follow-up work from both approaches. ORF8 showed significantly better solubility when produced in WG extracts compared to bacteria. This shows the necessity of parallel routes to take, in particular, for the understudied, biochemically nontrivial ORFs that might represent yet unexplored but highly specific targets to consider in the treatment of COVID-19.

Downstream structural analysis of ORFs produced with CFPS remains challenging but promising progress is being made in the light of SCoV2. Some solution NMR spectra show the expected number of signals with good resolution (e.g., ORF9b). As expected, however, most proteins cannot be straightforwardly analyzed by solution NMR in their current form, as they exhibit too large objects after insertion into micelles and/or by inherent oligomerization. Cell-free synthesized proteins can be inserted into membranes through reconstitution (Fogeron et al., 2015a; Fogeron et al., 2015b; Fogeron et al., 2016; Jirasko et al., 2020a; Jirasko et al., 2020b). Reconstitution will thus be the next step for many accessory proteins, but also for M and E, which were well produced by WG-CFPS. We will also exploit the straightforward deuteration in WG-CFPS (David et al., 2018; Wang et al., 2019; Jirasko et al., 2020a) that circumvents proton back-exchange, rendering denaturation and refolding steps obsolete (Tonelli et al., 2011). Nevertheless, the herein presented protocols for the production of non-nsps by WG-CFPS instantly enable their employment in binding studies and screening campaigns and thus provide a significant contribution to soon-to-come studies on SCoV2 proteins beyond the classical and convenient drug targets.

Altogether and judged by the ultimate need of exploiting recombinant SCoV2 proteins in vaccination and highly paralleled screening campaigns, we optimized sample amount, homogeneity, and long-term stability of samples. Our freely accessible protocols and accompanying NMR spectra now offer a great resource to be exploited for the unambiguous and reproducible production of SCoV2 proteins for the intended applications.

## Data Availability

Assignments of backbone chemical shifts have been deposited at BMRB for proteins, as shown in Table 2, indicated by their respective BMRB IDs. All expression constructs are available as plasmids from https://covid19-nmr.de/.

## Glossary

aa: Amino acid
BEST: Band-selective excitation short-transient
BMRB: Biomagnetic resonance databank
CFPS: Cell-free protein synthesis
CoV: Coronavirus
CTD: C-terminal domain
DEDD: Asp-Glu-Glu-Asp
DMS: Dimethylsulfate
E: Envelope protein
ED: Ectodomain
fl: Full-length
GB1: Protein G B1 domain
GD: Globular domain
GF: Gel filtration
GST: Glutathione-S-transferase
His: Hisx-tag
HSP: Heat-shock protein
HSQC: Heteronuclear single quantum coherence
IDP: Intrinsically disordered protein
IDR: Intrinsically disordered region
IEC: Ion exchange chromatography
IMAC: Immobilized metal ion affinity chromatography
IPRS: IMAC-protease cleavage-reverse IMAC-SEC;
M: Membrane protein
MERS: Middle East Respiratory Syndrome
MHV: Murine hepatitis virus
M^pro^: Main protease
MTase: Methyltransferase
N: Nucleocapsid protein
NAB: Nucleic acid–binding domain
nsp: Nonstructural protein
NTD: N-terminal domain
PL^pro^: Papain-like protease
RdRP: RNA-dependent RNA polymerase
S: Spike protein
SARS: Severe Acute Respiratory Syndrome
SEC: Size-exclusion chromatography
SUD: SARS unique domain
SUMO: Small ubiquitin-related modifier
TEV: Tobacco etch virus
TM: Transmembrane
TROSY: Transverse relaxation-optimized spectroscopy
Trx: Thioredoxin
Ubl: Ubiquitin-like domain
Ulp1: Ubiquitin-like specific protease 1
WG: Wheat-germ.
