# Supplementary material for: Large-Scale Recombinant Production of the SARS-CoV-2 Proteome for High-Throughput and Structural Biology Applications
Source: Front Mol Biosci. 2021 May 10;8:653148. doi: 10.3389/fmolb.2021.653148 (PMC8141814; doi:10.3389/fmolb.2021.653148)
Supplement: Supplementary file 1 [file DataSheet1.pdf]

Table S1: Overview of labs as assigned to protocols

Protein	System	Main protocol from group(s)	Protocol as „additional information" from group(s)
**nsp1**	Bacterial	Carlomagno (fl), Schlundt (GD)	Schlundt (fl)
**nsp2**	Bacterial	Laurents (CtDR)	-
**nsp3a**	Bacterial	Blackledge (UBl+IDR), Schlundt (UBl)	-
**nsp3b**	Bacterial	Schwalbe (Macrodomain)	Alfano (Macrodomain)
**nsp3c**	Bacterial	Spyroulias (SUD-N, SUD-NM, SUD-M, SUD-MC, SUD-C)	-
**nsp3d**	Bacterial	Schwalbe (PL^{pro})	Schwalbe (PL^{pro})
**nsp3e**	Bacterial	Schlundt (NAB)	Schlundt (NAB)
**nsp3Y**	Bacterial	Hoch (CoV-Y)	-
**nsp5**	Bacterial	Schwalbe (fl)	Schwalbe (fl, **A**-**D**), Orts (fl, **E**), Varga (fl, **F**), Bax ((fl, **G**-**H**), Martin ((fl, **I**)
**nsp7**	Bacterial	Henzler-Wildman/Kirchdoerfer (fl)	-
**nsp8**	Bacterial	Henzler-Wildman/Kirchdoerfer (fl)	-
**nsp9**	Bacterial	Schlundt (fl)	Schlundt (fl, **A**), Alfano (fl, **B**)
**nsp10**	Bacterial	Schwalbe (fl)	Jaudzems (fl)
**nsp13**	Bacterial	Schwalbe (fl)	Schwalbe (fl)
**nsp14**	Bacterial	Jaudzems (fl, MTase)	Schwalbe (fl)
**nsp15**	Bacterial	Schwalbe (fl)	-
**nsp16**	Bacterial	Jaudzems (fl)	Jaudzems (fl)
**ORF3a**	Cell-free	Böckmann (fl)	-
**Envelope (ORF4)**	Cell-free	Böckmann/Meier (fl)	-
**Membrane (ORF5)**	Cell-free	Böckmann/Meier (fl)	Böckmann/Meier (fl)
**ORF6**	Cell-free	Böckmann (fl)	Böckmann (fl)
**ORF7a**	Bacterial	Muhle-Goll (ED)	-
**ORF7b**	Bacterial	Schwalbe (fl)	Schwalbe (fl, **A**-**E**)
	Cell-free	Böckmann (fl)	-

**ORF8**	Bacterial	Wiedemann/Ohlenschläger (fl-L84S) Alfano (w/o signal peptide (Δ))	Wiedemann/Ohlenschläger (fl)
	Cell-free	Böckmann (fl, Δ)	-
**Nucleo-capsid (ORF9a)**	Bacterial	Pierattelli/Felli (IDR1-NTD-IDR2), Almeida (NTD-SR, NTD), Schlundt (CTD)	-
**ORF9b**	Cell-free	Böckmann (fl)	Böckmann (fl, **A**-**B**)
**ORF14**	Cell-free	Böckmann/Meier (fl)	-
**ORF10**	Bacterial	Schwalbe (fl)	Schwalbe (fl, **A**-**D**)

Table S2: Abbreviations used throughout the SI

**Abbreviation**	**Full name**
aa	Amino acid
AC	Affinity chromatography
BEST	Band-selective Excitation Short-Transient
BisTris	2,2-Bis(hydroxymethyl)-2,2′,2″-nitrilotriethanol
bME	2-mercaptoethanol
BMRB	Biomagnetic Resonance Databank
Brij 58	Polyethylene glycol hexadecyl ether, Polyoxyethylene (20) cetyl ether
CFPS	Cell-free protein synthesis
CFS	Cell-free sample
CoV	Coronavirus
CTD	C-terminal domain
DDM	n-dodecyl β-D-maltoside
E. coli	*Escherichia coli* cells
ED	Ectodomain
fl	Full-length
GB1	Protein G B1 domain
GD	Globular domain
GST	Glutathione-S-transferase
His_6 (analog His_7)	Hexahistidine tag
HSQC	Heteronuclear single quantum coherence
IDR	Intrinsically disordered region
IEC	Ion exchange chromatography
IMAC	Immobilized metal ion affinity chromatography
Inv.	Inverse
IPTG	Isopropyl-β-d-thiogalactopyranoside
LB medium	Lysogeny broth medium
M9 medium	M9 minimal medium
MOPS	3-(N-morpholino)propanesulfonic acid, 4-morpholinepropanesulfonic acid
M^{pro}	Main protease

MTase	Methyltransferase
MWCO	Molecular weight cut-off
NAB	Nucleic acid-binding domain
NaPi/KPi	Sodium/potassium phosphate
NA	Not available
n.d.	Not defined/no information available
nsp	Non-structural protein
NTA	Nitrilotriacetic acid
NTD	N-terminal domain
o.n.	Overnight
OD_{600}	Optical density at 600 nm
ORF	Open reading frame
PDB	Protein Data Bank
PL^{pro}	Papain-like protease
rt	Room temperature
S, SARS	Severe acute respiratory syndrome
SD	Superdex
SEC	Size exclusion chromatography
SN	Soluble fraction, supernatant
SUD	SARS unique domain
SUMO	Small ubiquitin-like modifier
TCEP	Tris-(2-carboxyethyl)-phosphin
TEV	Tobacco etch virus
Triton X-100	4-(1,1,3,3-Tetramethylbutyl)-phenyl-polyethylenglykol
TROSY	Transverse relaxation-optimized spectroscopy
Trx	Thioredoxin
Ubl	ubiquitin-like domain
Ulp1	ubiquitin-like specific protease 1
WB	Western blot
WG(E)	Wheat germ (extract)
YT medium	Yeast extract-tryptone medium

SI1: nsp1

Table 1: General Information

**1**	**Protein Name (according to NCBI Reference Sequence NC_045512.2)**
	ORF1a and ORF1ab; nsp1
**2**	**Region/Name/Further Specification**
	nsp1 / Leader protein
**3**	**Sequence of fl protein (according to NCBI Reference Sequence NC_045512.2)**
	MESLVPGFNEKTHVQLSLPVLQVRDVLVRGFGDSVEEVLSEARQHLKDGTCGLVEVEKGVLPQ<br>LEQPYVFIKRSDARTAPHGHVMVELVAELEGIQYGRSGETLGVLVPHVGEIPVAYRKVLLRKNG<br>NKGAGGHSYGADLKSFDLGDELGTDPYEDFQENWNTKHSSGVTRELMRELNGG
**4**	**Protein boundaries of expressed construct (according to NCBI Reference Sequence NC_045512.2)**
fl	aa 1-180 (fl nsp1)
GD	aa 13-127 of fl nsp1
**5**	**Ratio for construct design**
fl	fl sequence according to NCBI Reference Sequence YP_009725297.1
GD	In analogy to the available NMR structure (PDB 2GDT) of nsp1 SCoV 13-127
**6**	**Sequence homology (to SCoV)**
fl	Identity: 83%; similarity: 89%
GD	Identity: 85%; similarity: 90%
**7**	**Published structures (SCoV2 or homologue variants)**
	SCoV: PBD 2GDT, 2HSX<br>SCoV2: PBD 7K3N, 7K7P, 6ZN5, 7JQC, 7K5I
**8**	**(Published) assignment (SCoV2 or homologue variants)**
	SCoV: BMRB 7014<br>SCoV2: BMRB 50620

Table 2: Protein Expression

**1**	**Expression vector**
fl	pETM11 (Gunter Stier, EMBL Heidelberg)
GD	pKM263 (GenScript)
**2**	**Purification-/Solubility-Tag**
fl	N-terminal His_6
GD	N-terminal His_6
**3**	**Cleavage Site**
	TEV
**4**	**Molecular weight / Extinction coefficient / pI - of cleaved protein**

fl	19.90 kDa / 12,950 M^{-1} cm^{-1} / 5.37
GD	12.93 kDa / 4,470 M^{-1} cm^{-1} / 6.22
**5**	**Comments on sequence of expressed construct**
fl	N-terminal „GA" two artificial residues due to TEV-cleavage and construct design
GD	N-terminal „GAMA" four artificial residues due to TEV-cleavage and construct design
**6**	**Used expression strain**
	E. coli BL21 (DE3)
**7**	**Cultivation medium**
	LB / M9 (uniformly ^{15}N or ^{13}C,^{15}N-labelled)
**8**	**Induction system**
	IPTG inducible T7 promoter
**9**	**Induction of protein expression**
fl	0.6 mM IPTG at OD_{600} 0.7
GD	1 mM IPTG
**10**	**Cultivation temperature and time**
fl	16°C for 18-20 h
GD	16°C for 18-20 h

Table 3a: Protein Purification (fl nsp1)

**1**	**Buffer List**
A	50 mM Tris-HCl (pH 7.5), 500 mM NaCl, 100 mM Na_2SO_4, 5% (v/v) glycerol, 5 mM imidazole, 1 mM TCEP-HCl (cell disruption / immobilized metal affinity chromatography (IMAC) / TEV-cleavage).
B	50 mM Tris-HCl (pH 7.5), 500 mM NaCl, 100 mM Na_2SO_4, 1 mM EDTA, 1 mM TCEP-HCl (SEC).
C	50 mM NaPi (pH 6.5), 200 mM NaCl, 2 mM DTT, 2 mM EDTA (final NMR buffer).
**2**	**Purification steps (with corresponding buffer(s) and incubation times)**
A	Cell disruption in buffer **1A** (plus one tablet of EDTA free protease inhibitor cocktail (Roche), 100 µg of lysozyme (Carl Roth), and 50 µg of deoxyribonuclease (DNAse) (New England Biolabs)) by sonication.
B	IMAC (gravity flow Ni^{2+}-NTA (Cytiva)), washed first with buffer **1A** and then with buffer **1A** containing additional 2 M LiCl, before eluting with 300 mM imidazole in buffer **1A**.
C	Desalting and TEV-cleavage (0.5 mg TEV protease per 1 L culture) o.n. in buffer **1A**.
D	SEC on HiLoad 16/600 SD 75 (GE Healthcare) in buffer **1B**.
E	NMR sample preparation in buffer **1C**.

Table 3b: Protein Purification (GD nsp1)

**1**	**Buffer List**
A	50 mM Tris-HCl (pH 8.0), 300 mM NaCl, 10 mM imidazole, 4 mM DTT (cell disruption / IMAC/ dialysis after IMAC / TEV-cleavage).
B	25 mM NaPi (pH 7.0), 250 mM NaCl, 2 mM TCEP-HCl, 0.02% (w/v) NaN_3 (SEC / final NMR buffer).
**2**	**Purification steps (with corresponding buffer(s) and incubation times)**
A	Cell disruption in buffer **1A** (plus 100 µL protease inhibitor (Serva)) by sonication.
B	IMAC (gravity flow Ni^{2+}-NTA), Elution with 150-500 mM imidazole in buffer **1A**.
C	Dialysis o.n. in in buffer **1A**.
D	TEV-cleavage (0.5 mg TEV protease per 1 L culture) in buffer **1A**.
E	SEC on HiLoad SD 75 16/600 (GE Healthcare) in buffer **1B**.
F	NMR sample preparation in buffer **1B**.

Table 4: Final samples

**1**	**Yield**
fl	5 mg/L ^{13}C,^{15}N-M9 medium
GD	< 0.5 mg/L ^{15}N-M9 medium
**2**	**Stability**
fl	No significant precipitation or degradation observed after storage at 4°C for 3 weeks. Relatively stable during NMR measurements at 25°C for ~7 days, despite some proteolysis of disordered C-terminal tail.
GD	Stable during several weeks storage at 4°C.
**3**	**Comment on applicability**
fl	Suitable for NMR structure determination, fragment screening, interaction studies.
GD	purification needs optimization to obtain more soluble protein

Additional information

**Constructs**	**Conditions**	**Comments**
aa 1-180 (fl nsp1); His_7 (pET-TEV-Nco (GenScript)), TEV-cleavage site, N-terminal 2 artificial residues "GA".	As above for GD nsp1.	Yields 2.4 mg/L ^{15}N,^{13}C-M9 medium.<br>Obvious degradation during measurement. Storage at 4°C not advisable. Higher salt concentration seems to slightly improve stability.

SI2: nsp2

Table 1: General Information

1	**Protein Name (according to NCBI Reference Sequence NC_045512.2)**
	ORF1a and ORF1ab; nsp2
2	**Region/Name/Further Specification**
	C-terminal IDR (CtDR)
3	**Sequence of fl protein (according to NCBI Reference Sequence NC_045512.2)**
	AYTRYVDNNFCGPDGYPLECIKDLLARAGKASCTLSEQLDFIDTKRGVYCCREHEHEIAWYTE<br>RSEKSYELQTPFEIKLAKKFDTFNGECPNFVFPLNSIIKTIQPRVEKKKLDGFMGRIRSVYPVASP<br>NECNQMCLSTLMKCDHCGETSWQTGDFVKATCEFCGTENLTKEGATTCGYLPQNAVVKIYCP<br>ACHNSEVGPEHSLAEYHNESGLKTILRKGGRTIAFGGCVFSYVGCHNKCAYWVPRASANIGCN<br>HTGVVGEGSEGLNDNLLEILQKEKVNINIVGDFKLNEEIAIILASFSASTSAFVETVKGLDYKAFK<br>QIVESCGNFKVTKGKAKKGAWNIGEQKSILSPLYAFASEAARVVRSIFSRTLETAQNSVRVLQK<br>AAITILDGISQYSLRLIDAMMFTSDLATNNLVVMAYITGGVVQLTSQWLTNIFGTVYEKLKPVL<br>DWLEEKFKEGVEFLRDGWEIVKFISTCACEIVGGQIVTCAKEIKESVQTFFKLVNKFLALCADSII<br>IGGAKLKALNLGETFVTHSKGLYRKCVKSREETGLLMPLKAPKEIIFLEGETLPTEVLTEEVVLK<br>TGDLQPLEQPTSEAVEAPLVGTPVCINGLMLLEIKDTEKYCALAPNMMVTNNTFTLKGG
4	**Protein boundaries of expressed construct (according to NCBI Reference Sequence NC_045512.2)**
	aa 557-601 of complete nsp2 (Ct-DR)
5	**Ratio for construct design**
	Based on disorder predictions (PrDOS (Ishida and Kinoshita, 2007))
6	**Sequence homology (to SCoV)**
	Identity: 55%; similarity: 68%
7	**Published structures (SCoV2 or homologue variants)**
	-
8	**(Published) assignment (SCoV2 or homologue variants)**
	SCoV: 50687

Table 2: Protein Expression

1	**Expression vector**
	Home made plasmid derived from pET28b(+) (EMD Biosciences) containing the codifying sequence for thioredoxin A from *E. coli* and TEV protease cleavage site instead of thrombin.
2	**Purification-/Solubility-Tag**
	N-terminal His_6-Trx
3	**Cleavage Site**
	TEV
4	**Molecular weight / Extinction coefficient / pI - of cleaved protein**

	4.92 kDa / - / 3.9
**5**	**Comments on sequence of expressed construct**
	N-terminal „G“, one artificial residue due to TEV-cleavage.
**6**	**Used expression strain**
	E. coli BL21 star (DE3)
**7**	**Cultivation medium**
	LB / M9 (uniformly ^{15}N or ^{13}C,^{15}N-labelled)
**8**	**Induction system**
	IPTG inducible T7 promoter
**9**	**Induction of protein expression**
	0.5 mM IPTG at OD_{600} 0.6
**10**	**Cultivation temperature and time**
	37ºC until induction. Following induction, incubation at 25ºC for 17 h

Table 3: Protein Purification

**1**	**Buffer List**
A	50 mM Tris-HCl (pH 8.0), 300 mM NaCl, 10 mM imidazole (cell lysis, IMAC1 and 2).
B	5 mM Tris-HCl (pH 8.0), 20 mM NaCl (dialysis after IMAC1/TEV cleavage).
C	5 mM histidine (pH 5.4), 5 mM NaCl (dialysis after IMAC2 and anionic IEC).
D	10 mM acetic acid (pH 4.3), 5 mM NaCl (dialysis after cationic IEC).
**2**	**Purification steps (with corresponding buffer(s) and incubation times)**
A	Cell lysis in **1A** (plus 5 µL Halt protease inhibitor (Thermo) and lysozyme 20 µg/mL).
B	IMAC1 (HisTrap crude 5 mL, Cytiva). Elution 10-500 mM imidazole in buffer **1A**.
C	Dialysis in buffer **1B** and TEV cleavage (4ºC, 17 h).
D	IMAC2 (after TEV cleavage) (HisTrap crude 5 mL, Cytiva). Elution 10-500 mM imidazole in buffer **1A** (protein expected in flow-through).
E	Dialysis in buffer **1C** (4ºC, 17 h).
F	Anionic IEC, elution 10-1,000 mM NaCl in buffer **1C**.
G	Dialysis in buffer **1C** (4ºC, 48 h).
H	Cationic IEC. Elution 10-1,000 mM NaCl in buffer **1D** (protein expected in flow-through).

Table 4: Final sample

**1**	**Yield**
	1.5 mg/L LB medium, 0.7-1.5 mg/L ^{13}C, ^{15}N-M9 medium
**2**	**Stability**
	No visible precipitation after two weeks at 4ºC.
**3**	**Comment on applicability**
	Suitable for NMR structure determination, fragment screening, interaction studies.

SI3: nsp3a

Table 1: General Information

**1**	**Protein Name (according to NCBI Reference Sequence NC_045512.2)**
	ORF1a and ORF1ab; nsp3
**2**	**Region/Name/Further Specification**
	nsp3a Ubiquitin-like domain (Ubl) + IDR
**3**	**Sequence of “fl” protein (aa 1-206 of complete nsp3, according to NCBI Reference Sequence NC_045512.2)**
	APTKVTFGDDTVIEVQGYKSVNITFELDERIDKVLNEKCSAYTVELGTEVNEFACVVADAVIKT<br>LQPVSELLTPLGIDLDEWSMATYYLFDESGEFKLASHMYCSFYPPDEDEEEGDCEEEEFEPSTQY<br>EYGTEDDYQGKPLEFGATSAALQPEEEQEEDWLDDDSQQTVGQQDGSEDNQTTTIQTIVEVQP<br>QLEMELTPVVQTIE
**4**	**Protein boundaries of expressed construct (according to NCBI Reference Sequence NC_045512.2)**
Ubl+ IDR	aa 1-206 of complete nsp3
Ubl	aa 1-111 of complete nsp3
**5**	**Ratio for construct design**
Ubl+ IDR	Based on homologous structure from SCoV.
Ubl	Based on disorder prediction, folded domain and SCoV Ubl1.
**6**	**Sequence homology (to SCoV)**
Ubl+ IDR	Identity: 58%; Similarity: 75%
Ubl	Identity: 79%; Similarity: 89%
**7**	**Published structures (SCoV2 or homologue variants)**
	SCoV: PDB 2GRI; 2IDY
**8**	**(Published) assignment (SCoV2 or homologue variants)**
	SCoV: BMRB 7019<br>SCoV2: BMRB 50446

Table 2: Protein Expression

**1**	**Expression vector**
Ubl+ IDR	pET-TEV-Nco (GenScript)
Ubl	pKM263 (GenScript)
**2**	**Purification-/Solubility-Tag**
Ubl+ IDR	N-terminal His_6

Ubl	N-terminal His_6-GST
**3**	**Cleavage Site**
	TEV
**4**	**Molecular weight / Extinction coefficient / pI - of cleaved protein**
Ubl+ IDR	23.50 kDa / 24,410 M^{-1} cm^{-1} / 3.62
Ubl	12.72 kDa / 14,440 M^{-1} cm^{-1} / 4.08
**5**	**Comments on sequence of expressed construct**
Ubl+ IDR	N-terminal “GAM” three artificial residues due to TEV-cleavage and construct design.
Ubl	N-terminal “GAMG” four artificial residues due to TEV-cleavage and construct design.
**6**	**Used expression strain**
	E. coli BL21 (DE3)
**7**	**Cultivation medium**
	LB / M9 (uniformly ^{15}N or ^{13}C,^{15}N-labelled)
**8**	**Induction system**
	IPTG inducible T7 promoter
**9**	**Induction of protein expression**
	1 mM IPTG at OD_{600} 0.6-0.8
**10**	**Cultivation temperature and time**
Ubl+ IDR	37ºC for 5 h
Ubl	18°C for 18 h

Table 3a: Protein Purification (Ubl + IDR)

**1**	**Buffer List**
A	50 mM Tris-HCl (pH 8.0), 150 mM NaCl and complete EDTA-free tablet (cell disruption).
B	50 mM Tris-HCl (pH 8.0) and 150 mM NaCl (wash buffer).
C	50 mM Tris-HCl (pH 8.0), 150 mM NaCl and 500 mM imidazole (elution buffer).
D	50 mM Tris-HCl (pH 8.0), 150 mM NaCl and 5 mM bME (TEV cleavage).
E	50 mM NaPi (pH 6.5), 250 mM NaCl (final NMR buffer).
**2**	**Purification steps (with corresponding buffer(s) and incubation times)**
A	Resuspension of cell pellet in 50 mL per liter of culture of **1A** at 4°C.
B	Cell disruption by sonication on ice.

C	Clarification of lysate by centrifugation at 16,000 g for 30 min at 4°C.
D	Loading of lysate on Ni^{2+}-loaded IMAC resin (ThermoFisher scientific) pre-equilibrated with **1B** at 22°C.
E	Wash IMAC resin with 50 bed volumes of **1B**.
F	Elute protein from IMAC resin with 5 bed volumes of **1C**.
G	TEV cleavage with 1 mg TEV per 50 mg protein by dialysis against **1D** for 18 h at 4°C.
H	Removal of uncleaved protein and tag by elution through Ni^{2+}-loaded IMAC resin pre-equilibrated with **1B** at 22°C.
I	Wash with 5 bed volumes of **1B**.
J	SEC with HiLoad SD 75 pg column (GE Healthcare) pre-equilibrated with **1E** at 4°C.

Table 3b: Protein Purification (Ubl)

**1**	**Buffer List**
A	50 mM NaPi (pH 6.5), 300 mM NaCl, 10 mM imidazole, 2 mM TCEP-HCl (Cell disruption / IMAC)
B	25 mM NaPi (pH 7.0), 150 mM NaCl, 2 mM DTT, 0.02% NaN3 (dialysis after IMAC / TEV-cleavage)
C	25 mM NaPi (pH 7.0), 150 mM NaCl, 2 mM TCEP-HCl, 0.02% NaN_3, pH7 (SEC / final NMR buffer)
**2**	**Purification steps (with corresponding buffer(s) and incubation times)**
A	Cell disruption in buffer **1A** (plus 100 µL protease inhibitor (Serva)) by sonication.
B	IMAC (gravity flow Ni^{2+}-NTA), Elution with 150-500 mM imidazole in buffer **1A**
C	Dialysis o.n. in in buffer **1B**
D	TEV-cleavage (0.5 mg TEV protease per 1 L culture) in buffer **1B**
E	SEC on HiLoad 16/600 SD 75 (GE Healthcare) in buffer **1C**
F	NMR sample preparation in buffer **1C**

Table 4: Final sample

**1**	**Yield**
Ubl+ IDR	0.7 mg/L ^{15}N-M9 medium
Ubl	2-3 mg/L ^{15}N-M9 medium
**1b**	**A260/280 ratio**
Ubl+ IDR	0.57
Ubl	0.6
**2**	**Stability**
Ubl+	2 weeks at 25°C.

IDR	
Ubl	Very stable over weeks.
**3**	**Comment on applicability**
Ubl+ IDR	Stable for NMR assignments and screening
Ubl	Stable for NMR assignments and screening (spectra overlay with folded part of nsp3a Ubl + IDR above.)

SI3: nsp3b

Table 1: General Information

1	**Protein Name (according to NCBI Reference Sequence NC_045512.2)**
	ORF1a and ORF1ab; nsp3
2	**Region/Name/Further Specification**
	nsp3b / Macrodomain
3	**Sequence of "fl" protein (aa 207-376 of complete nsp3, according to NCBI Reference Sequence NC_045512.2)**
	VNSFSGYLKLTDNVYIKNADIVEEAKKVKPTVVVNAANVYLKHGGGVAGALNKATNNAMQV ESDDYIATNGPLKVGGSCVLSGHNLAKHCLHVVGPNVNKGEDIQLLKSAYENFNQHEVLLAPL LSAGIFGADPIHSLRVCVDTVRTNVYLAVFDKNLYDKLVSSFLEMK
4	**Protein boundaries of expressed construct (according to NCBI Reference Sequence NC_045512.2)**
	aa 207-376 of complete nsp3
5	**Ratio for construct design**
	Based on homologous structure from SCoV (PDB 6VXS).
6	**Sequence homology (to SCoV)**
	Identity: 74%; similarity: 84%
7	**Published structures (SCoV2 or homologue variants)**
	SCoV2: PDB 6W6Y, 6YWM, 6YWL, 6YWK, 6WEY, 7KG3, 6W02, 6WOJ, 6WEN, 6WCF, 6VXS, 7JME
8	**(Published) assignment (SCoV2 or homologue variants)**
	SCoV2: BMRB 50387 (apo), 50388 (holo)

Table 2: Protein Expression

1	**Expression vector**
	pET28a(+) (GenScript)
2	**Purification-/Solubility-Tag**
	N-terminal His_6
3	**Cleavage Site**
	TEV
4	**Molecular weight / Extinction coefficient / pI - of cleaved protein**
	18.65 kDa / 10,430 M^{-1} cm^{-1} / 7.20
5	**Comments on sequence of expressed construct**
	N-terminal "GHM" three artificial residues due to TEV-cleavage and construct design.

**6**	**Used expression strain**
	E. coli T7 Express
**7**	**Cultivation medium**
	LB / M9 (uniformly ^{15}N or ^{13}C,^{15}N-labelled)
**8**	**Induction system**
	IPTG inducible T7 promoter
**9**	**Induction of protein expression**
	0.2 mM IPTG at OD_{600} 0.6-0.7
**10**	**Cultivation temperature and time**
	18-20°C for 16-18 h

Table 3: Protein Purification

**1**	**Buffer List**
A	25 mM Tris-HCl (pH 8.0), 300 mM NaCl, 5 mM imidazole, 10 mM bME (cell disruption / IMAC).
B	25 mM Tris-HCl (pH 8.0), 300 mM NaCl, 10 mM bME (dialysis after IMAC / TEV-cleavage).
C	25 mM BisTris (pH 6.5), 150 mM NaCl, 3 mM TCEP-HCl (SEC / final NMR buffer).
**2**	**Purification steps (with corresponding buffer(s) and incubation times)**
A	Cell disruption in buffer **1A** (plus one tablet of EDTA free protease inhibitor cocktail (Merck)) by microfluidization.
B	IMAC (HisTrap HP (GE Healthcare), ÄKTA start (GE Healthcare)), elution with imidazole gradient up to 500 mM in buffer **1A.**
C	TEV-cleavage (1 mg TEV protease per 50 mL protein solution) o.n. in buffer **1B.**
D	Inv. IMAC (HisTrap HP (GE Healthcare), ÄKTA start (GE Healthcare)), elution with 500 mM imidazole in buffer **1A**.
E	SEC (HiLoad 26/600 SD 200 pg (GE Healthcare), ÄKTApurifier (GE Healthcare)) in buffer **1C** (elution volume 245-290 mL).
F	NMR sample preparation in buffer **1C.**

Table 4: Final sample

**1**	**Yield**
	94 mg/L ^{15}N-M9 medium, 9 mg/L ^{13}C,^{15}N-M9 medium
**2**	**Stability**
	Stable throughout measurement (7 days, 298 K). No significant precipitation or degradation observed after storage at 4°C for 2 weeks.
**3**	**Comment on applicability**

	Suitable for NMR structure determination, fragment screening, interaction studies.

Additional information

Constructs	Conditions	Comments
aa 206-374 of complete nsp3; His_6-GST (mod pET9d), TEV-cleavage site, N-terminal "GAM" three artificial residues. Based on boundaries from crystal structure (PDB 6W6Y).	**IMAC buffer:** 50 mM Tris-HCl (pH 8.0), 500 mM NaCl, 5% (v/v) glycerol, 50 mM imidazole, 1 mM DTT.<br>**Cleavage buffer:** 50 mM Tris-HCl (pH 8.0), 500 mM NaCl, 1 mM DTT.<br>**SEC/final buffer:** 20 mM NaPi (pH 7.4), 150 mM NaCl, 3 mM TCEP-HCl.	Yields 30 mg/L LB medium. No significant precipitation or degradation observed after storage at 4°C for 10 days. Suitable for NMR studies, fragment-based screening, interaction studies.

SI3: nsp3c

Table 1: General Information

**1**	**Protein Name (according to NCBI Reference Sequence NC_045512.2)**
	ORF1a and ORF1ab; nsp3
**2**	**Region/Name/Further Specification**
**SUD-N**	nsp3c / SARS Unique Domain (SUD) -N
**SUD-NM**	nsp3c / SUD-NM
**SUD-M**	nsp3c / SUD-M
**SUD-MC**	nsp3c / SUD-MC
**SUD-C**	nsp3c / SUD-C
**3**	**Sequence of "fl" protein (aa 409-743 of complete nsp3, according to NCBI Reference Sequence NC_045512.2)**
	QDDKKIKACVEEVTTTLEETKFLTENLLLYIDINGNLHPDSATLVSDIDITFLKKDAPYIVGDVV<br>QEGVLTAVVIPTKKAGGTTEMLAKALRKVPTDNYITTYPGQGLNGYTVEEAKTVLKKCKSAFY<br>ILPSIISNEKQEILGTVSWNLREMLAHAEETRKLMPVCVETKAIVSTIQRKYKGIKIQEGVVDYG<br>ARFYFYTSKTTVASLINTLNDLNETLVTMPLGYVTHGLNLEEAARYMRSLKVPATVSVSSPDA<br>VTAYNGYLTSSSKTPEEHFIETISLAGSYKDWSYSGQSTQLGIEFLKRGDKSVYYTSNPTTFHLD<br>GEVITFDNLKTLLS
**4**	**Protein boundaries of expressed construct (according to NCBI Reference Sequence NC_045512.2)**
**SUD-N**	aa 409-548 of complete nsp3
**SUD-NM**	aa 409-675 of complete nsp3
**SUD-M**	aa 551-675 of complete nsp3
**SUD-MC**	aa 551-743 of complete nsp3
**SUD-C**	aa 680-743 of complete nsp3
**5**	**Ratio for construct design**
**SUD-N**	Based on X-ray structure of homologue nsp3c from SCoV (PDB 2W2G).
**SUD-NM**	Based on X-ray structure of homologue nsp3c from SCoV (PDB 2W2G).
**SUD-M**	Based on X-ray structure of homologue nsp3c from SCoV (PDB 2W2G).
**SUD-MC**	Based on NMR structure of homologue nsp3c from SCoV (PDB 2KQV, 2KQW).
**SUD-C**	Based on NMR structure of homologue nsp3c from SCoV (PDB 2KAF).
**6**	**Sequence homology (to SCoV)**

**SUD-N**	Identity: 69%, similarity: 81.6%
**SUD-NM**	Identity: 74%, similarity: 85.4%
**SUD-M**	Identity: 82%, similarity: 89.6%
**SUD-MC**	Identity: 79%, similarity: 88.7%
**SUD-C**	Identity: 73%, similarity: 87.7%
**7**	**Published structures (SCoV2 or homologue variants)**
	-
**8**	**(Published) assignment (SCoV2 or homologue variants)**
**SUD-N**	SCoV2: BMRB 50448
**SUD-NM**	Ongoing
**SUD-M**	SCoV2: BMRB 50516 SUD-M
**SUD-MC**	Ongoing
**SUD-C**	SCoV2: BMRB 50517 SUD-C

Table 2: Protein Expression

**1**	**Expression vector**
**SUD-N**	pGEX4T1 (Addgene)
**SUD-NM**	pGEX4T1 (Addgene)
**SUD-M**	pET28a(+) (Addgene)
**SUD-MC**	pET28a(+) (Addgene)
**SUD-C**	pGEX4T1 (Addgene)
**2**	**Purification-/Solubility-Tag**
**SUD-N**	N-terminal GST
**SUD-NM**	N-terminal GST
**SUD-M**	N-terminal His_6
**SUD-MC**	N-terminal His_6
**SUD-C**	N-terminal GST

**3**	**Cleavage Site**
	Thrombin
**4**	**Molecular weight / Extinction coefficient / pI - of cleaved protein**
**SUD-N**	15.54 kDa / 8,940 M^{-1} cm^{-1} / 5.04
**SUD-NM**	29.60 kDa / 26,360 M^{-1} cm^{-1} / 6.03
**SUD-M**	14.27 kDa / 17,420 M^{-1} cm^{-1} / 8.71
**SUD-MC**	21.94 kDa / 28,880 M^{-1} cm^{-1} / 6.58
**SUD-C**	7.42 kDa / 11,460 M^{-1} cm^{-1} / 4.82
**5**	**Comments on sequence of expressed construct**
**SUD-N**	N-terminal „GS" two artificial residues due to thrombin-cleavage
**SUD-NM**	N-terminal „GS" two artificial residues due to thrombin-cleavage
**SUD-M**	N-terminal „GSHM" four artificial residues due to thrombin-cleavage and cloning
**SUD-MC**	N-terminal „GSHM" four artificial residues due to thrombin-cleavage and cloning
**SUD-C**	N-terminal „GS" two artificial residues due to thrombin-cleavage
**6**	**Used expression strain**
	E. coli BL21 (DE3)
**7**	**Cultivation medium**
	M9 (uniformly ^{15}N or $^{13}C,^{15}N$-labelled)
**8**	**Induction system**
	IPTG inducible T7 promoter
**9**	**Induction of protein expression**
	1 mM IPTG at OD_{600} 0.6-0.8
**10**	**Cultivation temperature and time**
	18˚C for 18-20 h

Table 3a: Protein Purification (SUD-N and SUD-NM)

**1**	**Buffer List**
A	50 mM Tris-HCl (pH 8.0), 300 mM NaCl (cell disruption / affinity chromatography (AC)).
B	50 mM NaPi (pH 7.2), 50 mM NaCl, 2 mM EDTA, 2 mM DTT (SEC / NMR buffer).

C	50 mM Tris-HCl (pH 8.0), 10 mM reduced glutathione (elution buffer).
**2**	**Purification steps (with corresponding buffer(s) and incubation times)**
A	Cell disruption in buffer **1A** (plus 25 µL protease inhibitor cocktail (Sigma Aldrich P8849) and 2 mM DTT) by sonication, after sonication incubation with 25 µL DNase (1 mg/mL) for 10 min on ice.
B	AC - GSTrap (GE Healthcare) (wash buffer **1A**).
C	Cleavage on column (100 µL thrombin (10 mg/mL) per 0.5 L culture) at 4˚C for 16 h.
D	Elution of SUD-N, SUD-NM after cleavage with buffer **1A**, elution of GST with buffer **1C** and buffer exchange with Amicon Ultra 15 mL centrifugal filter membrane (10,000 MWCO) (Merck Millipore) to buffer **1B**.
E	SEC - SD Increase 75 10/300 GL (GE Healthcare) in buffer **1B**.
F	NMR sample preparation in buffer **1B**.

Table 3b: Protein Purification (SUD-M and SUD-MC)

**1**	**Buffer List**
A	50 mM Tris-HCl (pH 8.0), 500 mM NaCl (Cell disruption / IMAC).
B **SUD-M**	50 mM NaPi (pH 7.2), 50 mM NaCl, 2 mM EDTA, 2 mM DTT (SEC / NMR buffer).
B **SUD-MC**	50 mM NaPi (pH 7.6), 50 mM NaCl, 2 mM EDTA, 2 mM DTT (SEC / NMR buffer).
**2**	**Purification steps (with corresponding buffer(s) and incubation times)**
A	Cell disruption in buffer **1A** (plus 10 mM imidazole and 25 µL protease inhibitor cocktail (Sigma Aldrich P8849) and 2 mM DTT) by sonication, before and after sonication incubation with 50 µL DNase (1 mg/mL) for 15 min on ice.
B	IMAC - HisTrap (Ni^{2+}) (GE Healthcare), a step gradient elution of imidazole in buffer **1A** (10, 20, 40, 100, 200, 400 mM). **SUD-M** eluted mostly in 100 mM imidazole in buffer **1A** and a small amount in fraction 200 mM imidazole in buffer **1A**. **SUD-MC** eluted mostly in 100 mM imidazole in buffer **1A** and a small amount in 40 mM imidazole in buffer **1A**.
C	Buffer exchange with Amicon Ultra 15 mL centrifugal filter membrane (10,000 MWCO) (Merck Millipore) in buffer **1B SUD-M** and **SUD-MC** respectively.
D	Cleavage in solution (100 µL thrombin (10 mg/mL) per 0.5 L culture) for **SUD-M**: 1 h at 4˚C and then 1 h at rt; **SUD-MC**: 16 h at 4˚C.
E	SEC - Superdex Increase 75 10/300 GL (GE Healthcare) in buffer **1C-SUD-M**, **1C-SUD-MC**.
F	NMR sample preparation in buffer **1C-SUD-M, 1C-SUD-MC**.

Table 3c: Protein Purification (SUD-C)

**1**	**Buffer List**
A	50 mM Tris-HCl (pH 8.0), 300 mM NaCl, 10% (v/v) glycerol (cell disruption / AC).
B	50 mM Tris-HCl (pH 8.0), 300 mM NaCl (AC).

C	50 mM Tris-HCl (pH 8.0), 10 mM reduced glutathione (elution buffer).
D	50 mM NaPi (pH 7.2), 50 mM NaCl, 2 mM EDTA, 2 mM DTT (SEC / NMR buffer).
**2**	**Purification steps (with corresponding buffer(s) and incubation times)**
A	Cell disruption in buffer **1A** (plus 25 µL protease inhibitor cocktail (Sigma Aldrich P8849) and 2 mM DTT) by sonication, after sonication incubation with 25 µL DNase (1 mg/mL) for 10 min on ice.
B	AC with GSTrap (GE Healthcare) (wash buffer **1A** and then wash with buffer **1B**).
C	Elution with buffer **1C**, buffer exchange with Amicon Ultra 15 mL centrifugal filter membrane (10,000 MWCO) (Merck Millipore) to buffer **1D**.
D	Cleavage in solution (350 µL thrombin (10 mg/mL) per 0.5 L culture) at 37˚C for 5 h.
E	SEC on SD Increase 75 10/300 GL (GE Healthcare) in buffer **1D**.
F	NMR sample preparation in buffer **1D**.

Table 4: Final sample

**1**	**Yield**
**SUD-N**	13.92 mg/L ^{15}N or $^{13}C,^{15}N$-M9 medium
**SUD-NM**	17.25 mg/L ^{15}N or $^{13}C,^{15}N$-M9 medium
**SUD-M**	8.47 mg/L ^{15}N or $^{13}C,^{15}N$-M9 medium
**SUD-MC**	12.06 mg/L ^{15}N or $^{13}C,^{15}N$-M9 medium
**SUD-C**	4.70 mg/L ^{15}N or $^{13}C,^{15}N$-M9 medium
**1b**	**A260/280 ratio**
**SUD-N**	0.55
**SUD-NM**	0.50
**SUD-M**	0.81
**SUD-MC**	0.62
**SUD-C**	0.71
**2**	**Stability**
**SUD-N**	Stable throughout NMR spectra acquisition (10 days, 298 K). No significant precipitation or degradation observed after thawing from -80˚C. Very stable construct.
**SUD-NM**	Stable throughout measurement (7 days, 298 K). No significant precipitation or degradation observed after defrosting from -80˚C.
**SUD-M**	Not very stable throughout spectra acquisition, 10 days 298 K. Significant precipitation observed after thawing from storage at -80˚C. Forms dimers without reducing agent observable even by SDS-page.

**SUD-MC**	Stable throughout measurement (7 days, 298 K). No significant precipitation or degradation observed after thawing from -80˚C.
**SUD-C**	Stable throughout measurement (10 days, 298 K). No significant precipitation or degradation observed after thawing from -80˚C. Stable construct.
**3**	**Comment on applicability**
	Suitable for NMR structure determination, fragment screening, interaction studies.

SI3: nsp3d

Table 1: General Information

1	**Protein Name (according to NCBI Reference Sequence NC_045512.2)**
	ORF1a and ORF1ab; nsp3
2	**Region/Name/Further Specification**
	nsp3d / papain-like protease / PL^{pro}
3	**Sequence of “fl” protein (aa 743-1060 of complete nsp3, according to NCBI Reference Sequence NC_045512.2)**
	SLREVRTIKVFTTVDNINLHTQVVDMSMTYGQQFGPTYLDGADVTKIKPHNSHEGKTFYVLPN<br>DDTLRVEAFEYYHTTDPSFLGRYMSALNHTKKWKYPQVNGLTSIKWADNNCYLATALLTLQQ<br>IELKFNPPALQDAYYRARAGEAANFCALILAYCNKTVGELGDVRETMSYLFQHANLDSCKRVL<br>NVVCKTCGQQQTTLKGVEAVMYMGTLSYEQFKKGVQIPCTCGKQATKYLVQQESPFVMMSA<br>PPAQYELKHGTFTCASEYTGNYQCGHYKHITSKETLYCIDGALLTKSSEYKGPITDVFYKENSY<br>TTTIK
4	**Protein boundaries of expressed construct (according to NCBI Reference Sequence NC_045512.2)**
	aa 743-1060 of complete nsp3
5	**Ratio for construct design**
	Based on homologous structure from SCoV (PDB 4M0W)
6	**Sequence homology (to SCoV)**
	Identity: 83%; similarity: 91%
7	**Published structures (SCoV2 or homologue variants)**
	SCoV: PDB 4M0W, 2FE8<br>SCoV2: PDB 6W9C
8	**(Published) assignment (SCoV2 or homologue variants)**
	-

Table 2: Protein Expression

1	**Expression vector**
	pE-SUMO (LifeSensors)
2	**Purification-/Solubility-Tag**
	N-terminal His_6-SUMO
3	**Cleavage Site**
	Ulp1
4	**Molecular weight / Extinction coefficient / pI - of cleaved protein**
	35.99 kDa / 45,270 M^{-1} cm^{-1} / 8.17
5	**Comments on sequence of expressed construct**

	No artificial residues due to Ulp1-cleavage and construct design.
**6**	**Used expression strain**
	E. coli BL21 (DE3)
**7**	**Cultivation medium**
	LB / M9 (uniformly ^{15}N-labelled)
**8**	**Induction system**
	IPTG inducible T7 promoter
**9**	**Induction of protein expression**
	0.2 mM IPTG at OD_{600} 0.6-0.7 (addition of 50 µM $ZnCl_2$)
**10**	**Cultivation temperature and time**
	18-20°C for 16-18 h

Table 3: Protein Purification

**1**	**Buffer List**
A	20 mM Tris-HCl (pH 8.0), 300 mM NaCl, 10 mM imidazole, 50 µM $ZnCl_2$, 10 mM bME (cell disruption / IMAC).
B	10 mM HEPES (pH 7.4), 100 mM NaCl, 50 µM $ZnCl_2$, 10 mM bME (dialysis after IMAC / TEV-cleavage).
C	10 mM HEPES (pH 7.4), 100 mM NaCl, 50 µM $ZnCl_2$, 5 mM DTT (SEC).
**2**	**Purification steps (with corresponding buffer(s) and incubation times)**
A	Cell disruption in buffer **1A** (addition of 50 µM $ZnCl_2$) by microfluidization.
B	IMAC (HisTrap HP (GE Healthcare), ÄKTA start (GE Healthcare)), elution with imidazole gradient up to 500 mM in buffer **1A.**
C	Ulp1-cleavage (1 mg TEV protease per 50 mL protein solution) o.n. in buffer **1B.**
D	Inv. IMAC (HisTrap HP (GE Healthcare), ÄKTA start (GE Healthcare)), elution with 500 mM imidazole in buffer **1A**.
E	SEC (HiLoad 26/600 SD 75 pg (GE Healthcare), ÄKTApurifier (GE Healthcare)) in buffer **1C** (elution volume 180-220 mL).

Table 4: Final sample

**1**	**Yield**
	12 mg/L ^{15}N-M9 medium
**2**	**Stability**
	Tendency to aggregate.
**3**	**Comment on applicability**

	Suitable for fragment screening, interaction studies.

Additional information

Constructs	Conditions	Comments
aa 743-1060 of complete nsp3; His_6 (pET28a(+) (GenScript)), TEV-cleavage site, N-terminal “GHM“ three artificial residues.	Native (as above)	Weak expression, less protein.

SI3: nsp3e

Table 1: General Information

1	**Protein Name (according to NCBI Reference Sequence NC_045512.2)**
	ORF1a and ORF1ab; nsp3
2	**Region/Name/Further Specification**
	nsp3e / NAB globular domain
3	**Sequence of "fl" protein (aa 1080-1203 of complete nsp3, according to NCBI Reference Sequence NC_045512.2)**
	YFTEQPIDLVPNQPYPNASFDNFKFVCDNIKFADDLNQLTGYKKPASRELKVTFFPDLNGDVVA IDYKHYTPSFKKGAKLLHKPIVWHVNNATNKATYKPNTWCIRCLWSTKPVET
4	**Protein boundaries of expressed construct (according to NCBI Reference Sequence NC_045512.2)**
	aa 1088-1203 of complete nsp3
5	**Ratio for construct design**
	Based on boundaries from NMR structure of homologue nsp3e from SARS-CoV (2K87).
6	**Sequence homology (to SCoV)**
	Identity: 82%; similarity: 89%
7	**Published structures (SCoV2 or homologue variants)**
	SCoV: PDB 2K87
8	**(Published) assignment (SCoV2 or homologue variants)**
	SCoV: BMRB 15723; SCoV2: BMRB 50334

Table 2: Protein Expression

1	**Expression vector**
	pKM263 (GenScript)
2	**Purification-/Solubility-Tag**
	N-terminal His_6-GST
3	**Cleavage Site**
	TEV
4	**Molecular weight / Extinction coefficient / pI - of cleaved protein**
	13.75 kDa / 25,565 M^{-1} cm^{-1} / 8.9
5	**Comments on sequence of expressed construct**
	N-terminal „GAMG" four artificial residues due to TEV-cleavage and construct design.
6	**Used expression strain**

	E. coli BL21 (DE3)
**7**	**Cultivation medium**
	LB / M9 (uniformly ^{15}N or ^{13}C,^{15}N-labelled)
**8**	**Induction system**
	IPTG inducible T7 promoter
**9**	**Induction of protein expression**
	1 mM IPTG at OD_{600} 0.7
**10**	**Cultivation temperature and time**
	20-22°C for 18-20 h

Table 3: Protein Purification

**1**	**Buffer List**
A	50 mM NaPi (pH 6.5), 300mM NaCl, 10 mM imidazole, 2 mM TCEP-HCl (cell disruption / IMAC).
B	25 mM NaPi (pH 7.0), 150 mM NaCl, 2 mM DTT, 0.02% (w/v) NaN_3 (dialysis after IMAC / TEV-cleavage).
C	25 mM NaPi (pH 7.0), 150 mM NaCl, 2 mM TCEP-HCl, 0.02% (w/v) NaN_3 (SEC / final NMR buffer).
**2**	**Purification steps (with corresponding buffer(s) and incubation times)**
A	Cell disruption in buffer **1A** (plus 100 µL protease inhibitor (Serva)) by sonication.
B	IMAC (gravity flow Ni^{2+}-NTA) (Carl Roth, Germany), elution with 150-500 mM imidazole in buffer **1A**.
C	Dialysis o.n. in buffer **1B**.
D	TEV-cleavage (0.5 mg TEV protease per 1 L culture) in buffer **1B**.
E	SEC on HiLoad 16/600 SD 75 (GE Healthcare) in buffer **1C**.
F	NMR sample preparation in buffer **1C**.

Table 4: Final sample

**1**	**Yield**
	3.5 mg/L ^{13}C, ^{15}N-M9 medium
**2**	**A260/280 ratio**
	0.74
**3**	**Stability**
	Stable throughout measurement (7 days, 298 K). No significant precipitation or degradation observed after storage at 4°C for 5 weeks.
**4**	**Comment on applicability**

	Suitable for NMR structure determination, fragment screening, interaction studies.

Additional information

**Constructs**	**Conditions**	**Comments**
NAB (aa 1088-1203) of complete nsp3; His_7 (pET-TEV-Nco (GenScript)), TEV-cleavage site, N-terminal “GAMG“ four artificial residues.	As above.	Works as well, but slightly less expression and yield.

SI3: nsp3Y

Table 1: General Information

1	**Protein Name (according to NCBI Reference Sequence NC_045512.2)**
	ORF1a and ORF1ab; nsp3
2	**Region/Name/Further Specification**
	nsp3-Y / Cov-Y
3	**Sequence of "fl" protein (aa 1638-1945 of complete nsp3, according to NCBI Reference Sequence NC_045512.2)**
	DTFCAGSTFISDEVARDLSLQFKRPINPTDQSSYIVDSVTVKNGSIHLYFDKAGQKTYERHSLSHF VNLDNLRANNTKGSLPINVIVFDGKSKCEESSAKSASVYYSQLMCQPILLLDQALVSDVGDSAE VAVKMFDAYVNTFSSTFNVPMEKLKTLVATAEAELAKNVSLDNVLSTFISAARQGFVDSDVET KDVVECLKLSHQSDIEVTGDSCNNYMLTYNKVENMTPRDLGACIDCSARHINAQVAKSHNIAL IWNVKDFMSLSEQLRKQIRSAAKKNNLPFKLTCATTRQVVNVVTTKIALKGG
4	**Protein boundaries of expressed construct (according to NCBI Reference Sequence NC_045512.2)**
	aa 1638-1945 of complete nsp3
5	**Ratio for construct design (detailed and comprehensible)**
	We took the C-terminal part of nsp3 after predicted transmembrane region and Y1 domain that consists of two sequential zinc finger motifs.
6	**Sequence homology (to SCoV)**
	Identity: 89%; similarity: 96%
7	**Published structures (SCoV2 or homologue variants)**
	-
8	**(Published) assignment (SCoV2 or homologue variants)**
	-

Table 2: Protein Expression

1	**Expression vector**
	pET28b(+) (GenScript)
2	**Purification-/Solubility-Tag**
	N-terminal His_6
3	**Cleavage Site**
	TEV
4	**Molecular weight / Extinction coefficient / pI - of cleaved protein**
	34 kDa / 17,420 M^{-1} cm^{-1} / 6.66
5	**Comments on sequence of expressed construct**

	N-terminal „G" one artificial residue due to TEV-cleavage.
**6**	**Used expression strain**
	E. coli BL21 (DE3)
**7**	**Cultivation medium**
	LB / M9 (uniformly ^{15}N or ^{13}C,^{15}N-labeling)
**8**	**Induction system**
	IPTG inducible T7 promoter
**9**	**Induction of protein expression**
	0.5 mM IPTG at OD_{600} 0.7
**10**	**Cultivation temperature and time**
	18°C for 15-16 h

Table 3: Protein Purification

**1**	**Buffer List**
A	20 mM Tris-HCl (pH 8.0), 300 mM NaCl, 10 mM imidazole, 0.1 mM PMSF, 5 mM bME, 0.1 mg/mL lysozyme, cOmplete EDTA-free inhibitor (Cell disruption).
B	20 mM Tris-HCl (pH 8.0), 300 mM NaCl, 20 mM imidazole (IMAC).
C	50 mM Tris-HCl (pH 8.0), 200 mM NaCl, 2 mM DTT (TEV-cleavage).
D	50 mM HEPES (pH 6.9), 200 mM LiBr, 5 mM DTT.
**2**	**Purification steps (with corresponding buffer(s) and incubation times)**
A	Cell disruption in buffer **1A** by sonication.
B	IMAC (gravity flow Ni^{2+}-NTA) (Thermo Scientific), wash with buffer **1B** and elution with 250 mM imidazole in buffer **1B**.
C	TEV-cleavage (5% (w/w) TEV protease per approximate amount of the protein) in buffer **1C** o.n. at rt.
D	Inv. IMAC (gravity flow Ni^{2+}-NTA) in buffer **1C**.
E	SEC on 10/300 GL SD 200 (GE Healthcare) in buffer **1D**.

Table 4: Final sample

**1**	**Yield**
	12 mg/L ^{13}C,^{15}N-M9 medium
**2**	**Stability**
	Stable at 25°C at protein concentration below 0.4 mM for 3 to 5 days or at 30°C o.n.. The protein gradually degrades at rt. After one week, we observe an additional band on SDS gel at ~27 kDa.
**3**	**Comment on applicability**

	The protein is suitable for NMR assignment and protein interaction studies at low temperature (20-25°C) and reasonably low concentration (< 0.2 mM).

SI4: nsp5

Table 1: General Information

1	**Protein Name (according to NCBI Reference Sequence NC_045512.2)**
	ORF1a and ORF1ab; nsp5
2	**Region/Name/Further Specification**
	3C-like protease (3CL^{pro}) / main protease (M^{pro})
3	**Sequence of fl protein (according to NCBI Reference Sequence NC_045512.2)**
	SGFRKMAFPSGKVEGCMVQVTCGTTTLNGLWLDDVVYCPRHVICTSEDMLNPNYEDLLIRKSN<br>HNFLVQAGNVQLRVIGHSMQNCVLKLKVDTANPKTPKYKFVRIQPGQTFSVLACYNGSPSGVY<br>QCAMRPNFTIKGSFLNGSCGSVGFNIDYDCVSFCYMHHMELPTGVHAGTDLEGNFYGPFVDRQ<br>TAQAAGTDTTITVNVLAWLYAAVINGDRWFLNRFTTTLNDFNLVAMKYNYEPLTQDHVDILG<br>PLSAQTGIAVLDMCASLKELLQNGMNGRTILGSALLEDEFTPFDVVRQCSGVTFQ
4	**Protein boundaries of expressed construct (according to NCBI Reference Sequence NC_045512.2)**
	aa 1-306 (fl nsp5)
5	**Ratio for construct design**
	fl protein
6	**Sequence homology (to SCoV)**
	Identity: 96%; similarity: 99.7%
7	**Published structures (SCoV2 or homologue variants)**
	SCoV: PDB 1P9U, 6LU7<br>SCoV2: PDB 6Y2E, 5R7Y, 6Y84, 7K3T
8	**(Published) assignment (SCoV2 or homologue variants)**
	SCoV: BMRB 17251

Table 2: Protein Expression

1	**Expression vector**
	pE-SUMO (LifeSensors)
2	**Purification-/Solubility-Tag**
	N-terminal His_6-SUMO
3	**Cleavage Site**
	Ulp1
4	**Molecular weight / Extinction coefficient / pI - of cleaved protein**
	33.80 kDa / 32,890 M^{-1} cm^{-1} / 5.95
5	**Comments on sequence of expressed construct**
	No artificial residues due to TEV-cleavage and construct design.

**6**	**Used expression strain**
	E. coli BL21 (DE3)
**7**	**Cultivation medium**
	LB / M9 (uniformly ^{15}N-labelled)
**8**	**Induction system**
	IPTG inducible T7 promoter
**9**	**Induction of protein expression**
	0.2 mM IPTG at OD_{600} 0.6-0.7
**10**	**Cultivation temperature and time**
	18-20°C for 16-18 h

Table 3: Protein Purification

**1**	**Buffer List**
A	50 mM NaPi (pH 7.5), 300 mM NaCl, 5 mM imidazole, 5% (v/v) glycerol, 10 mM bME (cell disruption / IMAC).
B	50 mM NaPi (pH 7.0), 300 mM NaCl, 10 mM bME, 5% (v/v) glycerol (dialysis after IMAC / Ulp1-cleavage).
C	25 mM NaPi (pH 7.5), 150 mM NaCl, 2 mM TCEP-HCl (SEC buffer).
D	10 mM NaPi (pH 7.0), 0.5 mM TCEP-HCl (final NMR buffer).
**2**	**Purification steps (with corresponding buffer(s) and incubation times)**
A	Cell disruption in buffer **1A** (plus one tablet of EDTA free protease inhibitor cocktail (Merck)) by microfluidization.
B	IMAC (HisTrap HP (GE Healthcare), ÄKTA start (GE Healthcare)), elution with imidazole gradient up to 500 mM in buffer **1A**.
C	Ulp1-cleavage (1 mg TEV protease per 50 mL protein solution) o.n. in buffer **1B**.
D	Inv. IMAC (HisTrap HP (GE Healthcare), ÄKTA start (GE Healthcare)), elution with 500 mM imidazole in buffer **1A**.
E	SEC (HiLoad 26/600 SD 75 pg (GE Healthcare), ÄKTApurifier (GE Healthcare)) in buffer **1C** (elution volume 170-210 mL).
F	NMR sample preparation in buffer **1D**.

Table 4: Final sample

**1**	**Yield**
	55 mg/L ^{15}N-M9 medium
**2**	**Stability**
	No significant precipitation or degradation observed after storage at -80°C for a month.

3	**Comment on applicability**
	Suitable for NMR structure determination, fragment screening, interaction studies.

Additional information

	**Constructs**	**Conditions**	**Comments**
**A**	aa 1-306 (fl nsp5) C145A mutation; His_6-SUMO (pE-SUMO (LifeSensors)), Ulp1-cleavage site, no N-terminal artificial residues.	Native (as above)	Comparable to fl nsp5 expression and purification, similar yield (80 mg/L ^{15}N-M9 medium).
**B**	aa 1-306 (fl nsp5); His_6-SUMO (pE-SUMO (LifeSensors)), Ulp1-cleavage site, N-terminal "GS" two artificial residues.	Native (as above)	Comparable to fl nsp5 expression and purification, similar yield (55 mg/L ^{15}N-M9 medium, 36 mg/L ^{13}C,^{15}N-M9 medium, 20 mg/L ^{2}H,^{13}C,^{15}N E. coli-OD2 CDN medium (Silantes)).
**C**	aa 1-306 (fl nsp5) C145A mutation; His_6-SUMO (pE-SUMO (LifeSensors)), Ulp1-cleavage site, N-terminal "GS" two artificial residues.	Native (as above)	Comparable to fl nsp5 expression and purification, similar yield (55 mg/L ^{15}N-M9 medium).
**D**	aa 1-306 (fl nsp5); His_6 (pet28a(+) (GenScript)), TEV-cleavage site; N-terminal "GHM" three artificial residues.	Native (as above)<br>**IMAC buffer (1A)**: 25 mM Tris-HCl (pH 8.0), 300 mM NaCl, 5 mM imidazole, 5% (v/v) glycerol, 10 mM bME	Comparable to fl nsp5 purification, however, less expression/yield (35 mg/L ^{15}N-M9 medium, 10 mg/L ^{13}C,^{15}N-M9 medium).
**E**	aa 1-306 (fl nsp5); GST and His_6-tag (pET-28a(+) (GenScript)), TEV and auto cleavage site for M^{pro}, N-terminal „GS" and C-terminal "GPHHHHHH" ten artificial residues.	**IMAC buffer:** 50 mM Tris-HCl (pH 8.0), 200 mM NaCl, 20 mM imidazole.<br>**SEC-buffer:** 50 mM NaPi (pH 7.6), 50 mM NaCl, 0.02% (w/v) NaN_3.<br>**NMR-buffer:** 50 mM NaPi (pH 7.6), 50 mM NaCl, 0.02% (w/v) NaN_3, 5 mM bME.	Yields 20 mg/L ^{15}N-M9 medium. The protein is stable up to 350 µM in NMR buffer at 25°C for at least 7 days.<br>At 50 µM and at 4°C, the protein is stable for ~15 days.<br>The protein is not suitable for freeze/thaw and results in precipitation.
**F**	aa 1-306 (fl nsp5); C-terminal His_6-tag (pET21b(+) (GenScript)), human rhinovirus 3-C protease cleavage site, N-terminal "M" additional aa, however our mass spectrum results suggest that M1 was removed by *E. coli* methionine aminopeptidase.	**IMAC buffer:** 20 mM Tris-HCl (pH 7.33), 150 mM NaCl, 20 mM imidazole.<br>**Storage buffer:** 20 mM Tris-HCl (pH 7.33), 150 mM NaCl.	Yields 5 mg/L ^{15}N-M9 medium. Stable for 2-3 weeks at 4°C at low micromolar concentration.
**G**	aa 1-306 (fl nsp5) C145A mutation; His_6-GB1 (pET24a(+) (GenScript)), TEV-cleavage site, no artificial residues.	**IMAC buffer:** 20 mM Tris-HCl (pH 7.5), 150 mM NaCl, 20 mM imidazole, 0.5 mM TCEP-HCl.<br>**SEC/NMR buffer:** 10 mM NaPi (pH 7.0), 0.5 mM TCEP-HCl.	Yields ≥ 70 mg/L ^{15}N, ^{2}H,^{15}N-M9, and ^{2}H,^{13}C,^{15}N-M9 medium. 1-2 mM sample stable for several weeks at 25°C. Negligible preci-pitation on freeze-thaw. Samples stable for ≥ 3 months at 80°C.<br>Sample precipitation in buffer: 10 mM NaPi (pH 7.0), 0.4 M GdnHCl.

**H**	aa 1-306 (fl nsp5); His$_6$-GB1 (pET24a(+) (GenScript)), TEV-cleavage site, no artificial residues.	As above (**G**).	Negligible expression when induced in ^{15}N-M9 medium at 25˚C, 30˚C, and 37˚C, with 0.5-1 mM IPTG.
**I**	aa 1-306 (fl nsp5); His$_6$-GST (pGEX-6p-1 (Genewiz)), autolytic and HRV 3C cleavage site, no artificial residues.	**IMAC buffer:** 25 mM Tris-HCl (pH 7.8), 150 mM NaCl, 5 mM imidazole, 1 mM bME.<br>**Cleavage buffer:** 25 mM Tris-HCl (pH 7.8), 150 mM NaCl, 1 mM DTT.<br>**SEC buffer:** 25 mM Tris-HCl (pH 7.8), 150 mM NaCl, 1 mM DTT, 1 mM EDTA.	40-60 mg/mL autoinduction Media ZYM-5052. Stored at 1 mg/mL at -20°C with 30% v/v) glycerol in SEC buffer.<br>Also stored at 25 mg/mL at -80˚C in SEC buffer. Flash frozen. Neither show loss of activity compared to non-frozen samples.

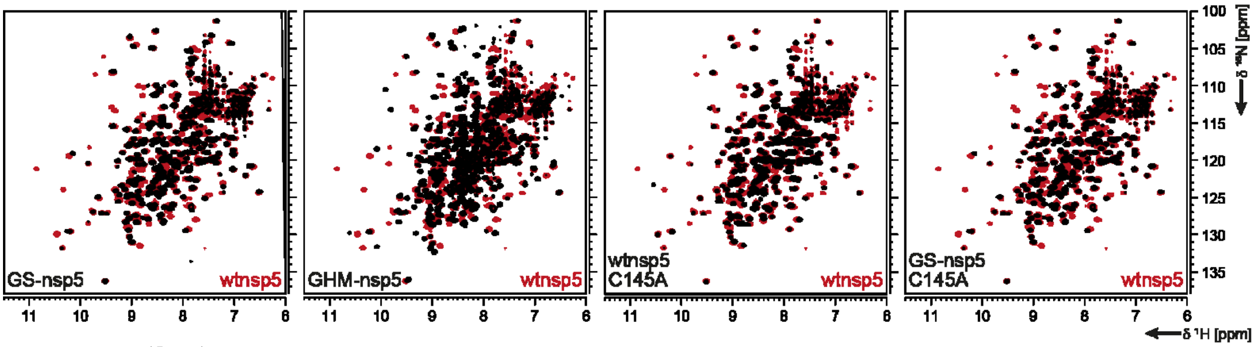

Overlays of [^{15}N, ^{1}H]-BEST TROSY spectra of wtnsp5 (red) with the other constructs (black). From left to right: N-terminally GS added nsp5 (GS-nsp5), GHM added (GHM-nsp5), the active site mutants C145A with native N-terminus (wtnsp5 C145A), and GS added mutant (GS-nsp5 C145A).

SI5: nsp7

Table 1: General Information

1	**Protein Name (according to NCBI Reference Sequence NC_045512.2)**
	ORF1a and ORF1ab; nsp7
2	**Region/Name/Further Specification**
	nsp7
3	**Sequence of fl protein (according to NCBI Reference Sequence NC_045512.2)**
	SKMSDVKCTSVVLLSVLQQLRVESSSKLWAQCVQLHNDILLAKDTTEAFEKMVSLLSVLLSMQ GAVDINKLCEEMLDNRATLQ
4	**Protein boundaries of expressed construct (according to NCBI Reference Sequence NC_045512.2)**
	aa 1-83 (fl nsp7)
5	**Ratio for construct design**
	fl protein
6	**Sequence homology (to SCoV)**
	Identity: 98.8%; similarity: 100%
7	**Published structures (SCoV2 or homologue variants)**
	SCoV: PDB 2KYS, 1YSY, 6NUS, 6NUR, 2AHM,<br>SCoV2: PDB 7BV2, 7BV1, 6YYT, 7BTF, 6WQD, 6WTC, 6WIQ, 6M71, 6YHU, 6XEZ, 6M5I, 7CTT, 7C2K, 7BW4, 7BZF, 7JLT, 7AAP, 6XIP, 6XQB
8	**(Published) assignment (SCoV2 or homologue variants)**
	SCoV: PDB 1YSY, BMRB 6513, PDB 2KYS, BMRB 16981<br>SCoV2: BMRB 50337

Table 2: Protein Expression

1	**Expression vector**
	pET46
2	**Purification-/Solubility-Tag**
	N-terminal His_6, enterokinase
3	**Cleavage Site**
	TEV
4	**Molecular weight / Extinction coefficient / pI - of cleaved protein**
	9.24 kDa / 5500 $cm^{-1} M^{-1}$ / 5.2
5	**Comments on sequence of expressed construct**
	N-terminal “G” an artificial residue due to TEV-cleavage.

**6**	**Used expression strain**
	E. coli Rosetta2 pLysS
**7**	**Cultivation medium**
	M9 (uniformly ^{15}N, ^{13}C-labelled)
**8**	**Induction system**
	IPTG inducible T7 promoter
**9**	**Induction of protein expression**
	0.5 mM IPTG at OD_{600} 0.8
**10**	**Cultivation temperature and time**
	16˚C for 14-16 h

Table 3: Protein Purification

**1**	**Buffer List**
A	10 mM HEPES (pH 7.4), 300 mM NaCl, 30 mM imidazole, 2 mM DTT.
B	10 mM HEPES (pH 7.4), 300 mM NaCl, 300 mM imidazole, 2 mM DTT.
C	10 mM MOPS (pH 7.0), 150 mM NaCl, 2 mM DTT.
**2**	**Purification steps (with corresponding buffer(s) and incubation times)**
A	Cell lysis in buffer **1A** by microfluidizer operating at 20,000 psi. Lysates were cleared by centrifugation at 25,000 g for 30 min and then filtered through a 0.45 µm filter.<br>Ni-NTA Agarose beads (Qiagen) were added to cleared lysates and incubated for 30 min. Beads were collected by centrifugation and then loaded onto a gravity column. Beads were washed twice with 10 column volumes of buffer **1A**. Protein was eluted with 5 column volumes of buffer **1B.**
B	Eluted protein was cleaved with 1% (w/w) TEV protease o.n. at rt while dialyzing the protein into 1 L buffer **1C**. Uncleaved protein was removed by inv. Ni-NTA binding.
C	Protein was concentrated using a 10 kDa MWCO (Amicon) concentrator and purified on an SD 200 Increase 10/300 (GE Life Sciences) size exclusion column, AKTApure (GE Life Sciences) using buffer **1C**.

Table 4: Final sample

**1**	**Yield**
	17 mg/L ^{13}C,^{15}N-M9 medium
**1b**	**A260/280 ratio**
	0.5
**2**	**Stability**
	NMR sample stable at 4°C for a month, at 35°C for several days before degradation occurs.
**3**	**Comment on applicability**

	Suitable for NMR-based screening applications.

SI6: nsp8

Table 1: General Information

1	**Protein Name (according to NCBI Reference Sequence NC_045512.2)**
	ORF1a and ORF1ab; nsp8
2	**Region/Name/Further Specification**
	nsp8
3	**Sequence of fl protein (according to NCBI Reference Sequence NC_045512.2)**
	AIASEFSSLPSYAAFATAQEAYEQAVANGDSEVVLKKLKKSLNVAKSEFDRDAAMQRKLEKM ADQAMTQMYKQARSEDKRAKVTSAMQTMLFTMLRKLDNDALNNIINNARDGCVPLNIIPLTT AAKLMVVIPDYNTYKNTCDGTTFTYASALWEIQQVVDADSKIVQLSEISMDNSPNLAWPLIVT ALRANSAVKLQ
4	**Protein boundaries of expressed construct (according to NCBI Reference Sequence NC_045512.2)**
	aa 1-198 (fl nsp8)
5	**Ratio for construct design**
	fl protein
6	**Sequence homology (to SCoV)**
	Identity: 97%; similarity: 98%
7	**Published structures (SCoV2 or homologue variants)**
	SCoV: PDB 6NUS, 6NUR, 2AHM,<br>SCoV2: PDB 7C2K, 7BV2, 7BV1, 7CTT, 6M5I, 7BW4, 6XEZ, 7BZF, 6XQB, 6M71, 6YYT, 7BTF, 7JLT, 7AAP, 6WIQ, 6XIP, 6WQD, 6WTC, 6YHU
8	**(Published) assignment (SCoV2 or homologue variants)**
	-

Table 2: Protein Expression

1	**Expression vector**
	pET46
2	**Purification-/Solubility-Tag**
	N-terminal His_6, enterokinase
3	**Cleavage Site**
	TEV
4	**Molecular weight / Extinction coefficient / pI - of cleaved protein**
	21.94 kDa / 19,940 $cm^{-1}M^{-1}$ / 6.5
5	**Comments on sequence of expressed construct**
	N-terminal "G" an artificial residue due to TEV-cleavage.

**6**	**Used expression strain**
	E. coli Rosetta2 pLysS
**7**	**Cultivation medium**
	M9 (uniformly ^{15}N-, ^{13}C-labelled)
**8**	**Induction system**
	IPTG inducible T7 promoter
**9**	**Induction of protein expression**
	0.5 mM IPTG at OD_{600} 0.8
**10**	**Cultivation temperature and time**
	16˚C for 16-18 h

Table 3: Protein Purification

**1**	**Buffer List**
A	10 mM HEPES (pH 7.4), 300 mM NaCl, 30 mM imidazole, 2 mM DTT.
B	10 mM HEPES (pH 7.4), 300 mM NaCl, 300 mM imidazole, 2 mM DTT.
C	10 mM MOPS (pH 7.0), 300 mM NaCl, 2 mM DTT.
D	10 mM MOPS (pH 7.0), 150 mM NaCl, 2 mM DTT.
**2**	**Purification steps (with corresponding buffer(s) and incubation times)**
A	Cell lysis in buffer **1A** by microfluidizer operating at 20,000 psi. Lysates were cleared by centrifugation at 25,000 g for 30 min and then filtered through a 0.45 µm filter.<br>Ni-NTA Agarose beads (Qiagen) were added to cleared lysates and incubated for 30 min. Beads were collected by centrifugation and then loaded onto a gravity column. Beads were washed twice with 10 column volumes of buffer **1A**. Protein was eluted with 5 column volumes of buffer **1B.**
B	Eluted protein was cleaved with 1% (w/w) TEV protease o.n. at rt while dialyzing the protein into 1 L buffer **1C**. Uncleaved protein was removed by inverse Ni-NTA binding.
C	Protein was concentrated using a 10 kDa MWCO (Amicon) concentrator and purified on an SD 200 Increase 10/300 (GE Life Sciences) size exclusion column, AKTApure (GE Life Sciences) using buffer **1D**.

Table 4: Final sample

**1**	**Yield**
	17 mg/L ^{13}C,^{15}N-M9 medium
**1b**	**A260/280 ratio**
	0.5
**2**	**Stability**
	Concentration dependent aggregation of nsp8 observed in the range of 0.1-1.1 mM by NMR.

3	**Comment on applicability**
	Suitable for NMR-based screening approach.

SI7: nsp9

Table 1: General Information

1	**Protein Name (according to NCBI Reference Sequence NC_045512.2)**
	ORF1a and ORF1ab; nsp9
2	**Region/Name/Further Specification**
	nsp9
3	**Sequence of fl protein (according to NCBI Reference Sequence NC_045512.2)**
	NNELSPVALRQMSCAAGTTQTACTDDNALAYYNTTKGGRFVLALLSDLQDLKWARFPKSDGT GTIYTELEPPCRFVTDTPKGPKVKYLYFIKGLNNLNRGMVLGSLAATVRLQ
4	**Protein boundaries of expressed construct (according to NCBI Reference Sequence NC_045512.2)**
	aa 1-113 (fl nsp9)
5	**Ratio for construct design (detailed and comprehensible)**
	In analogy to the available crystal structure (PDB 1QZ8) of nsp9 SCoV, fl sequence.
6	**Sequence homology (to SCoV)**
	Identity: 97%; similarity: 97%
7	**Published structures (SCoV2 or homologue variants)**
	SCoV: PDB 3EE7 (G104E), 1UW7, 1QZ8<br>SCoV2: PDB 6WXD, 6W4B, 6W9Q
8	**(Published) assignment (SCoV2 or homologue variants)**
	SCoV: BMRB 6501<br>SCoV2: BMRB 50621, 50622

Table 2: Protein Expression

1	**Expression vector**
	pKM263 (GenScript)
2	**Purification-/Solubility-Tag**
	N-terminal His_6-GST
3	**Cleavage Site**
	TEV
4	**Molecular weight / Extinction coefficient / pI - of cleaved protein**
	12,7 kDa / 13,075 M^{-1} cm^{-1} / 9.1
5	**Comments on sequence of expressed construct**
	N-terminal „GAMG" four artificial residues due to TEV-cleavage and construct design
6	**Used expression strain**

	E. coli BL21 (DE3)
**7**	**Cultivation medium**
	LB / M9 (uniformly ^{15}N or $^{13}C,^{15}N$-labelled)
**8**	**Induction system**
	IPTG inducible T7 promoter
**9**	**Induction of protein expression**
	1 mM IPTG at OD_{600} 0.7
**10**	**Cultivation temperature and time**
	20-22°C for 18-20 h

Table 3: Protein Purification

**1**	**Buffer List**
A	50 mM Tris-HCl (pH 8.0), 300 mM NaCl, 10 mM imidazole, 4 mM DTT (cell disruption / IMAC/ dialysis after IMAC / TEV-cleavage).
B	25 mM NaPi (pH 7.0), 150 mM NaCl, 2 mM TCEP-HCl, 0.02% (w/v) NaN_3 (SEC / final NMR buffer).
**2**	**Purification steps (with corresponding buffer(s) and incubation times)**
A	Cell disruption in buffer **1A** (plus 100 µL protease inhibitor (Serva)) by sonication.
B	IMAC (gravity flow Ni^{2+}-NTA (Carl Roth)), Elution with 150-500 mM imidazole in buffer **1A**.
C	Dialysis o.n. in in buffer **1A**.
D	TEV-cleavage (0.5 mg TEV protease per 1 L culture) in buffer **1A**.
E	SEC on HiLoad 16/600 SD 75 (GE Healthcare) in buffer **1B**. See relevant peak in attached SEC profile.
F	NMR sample preparation in buffer **1B**.

Table 4: Final sample

**1**	**Yield**
	4.5 mg/L ^{13}C, ^{15}N-M9 medium
**1b**	**A260/280 ratio**
	0.7
**2**	**Stability**
	Stable dimer. Storage at 4°C possible.
**3**	**Comment on applicability**
	Conditions for NMR structure determination may need to be optimized (concerning line width due to dimeric state). Backbone assignment and screening successful.

Additional information

	Constructs	Conditions	Comments
**A**	aa 1-113 (fl nsp9); His_7 (pET-TEV-Nco (GenScript)), TEV-cleavage site, N-terminal "GAMG" four artificial residues.	As above.	Expression and purification as for GST-tagged fl nsp9, but lower expression and yield.
**B**		**IMAC buffer:** 25 mM NaPi (pH 7.4), 300 mM NaCl, 20 mM imidazole, 1 mM DTT.<br>**Cleavage buffer:** 25 mM NaPi (pH 7.4), 150 mM NaCl, 1 mM DTT.<br>**SEC/NMR buffer A:** 25 mM NaPi (pH 7.0), 150 mM NaCl, 1 mM DTT, 150 mM NaCl, 2 mM TCEP-HCl.<br>**SEC/NMR buffer B:** 25 mM NaAc (pH 5.0), 150 mM NaCl, 2 mM TCEP-HCl.	3 mg/L $^{13}C,^{15}N$-M9 medium. Sample in Buffer **A** looked degraded (from the ^{15}N HSQC) after 5 days of ^{13}C 3D NMR experiments at 298 K. Less degradation was observed for sample in Buffer **B** after same period. Suitable for NMR studies, fragment-based screening, interaction studies.

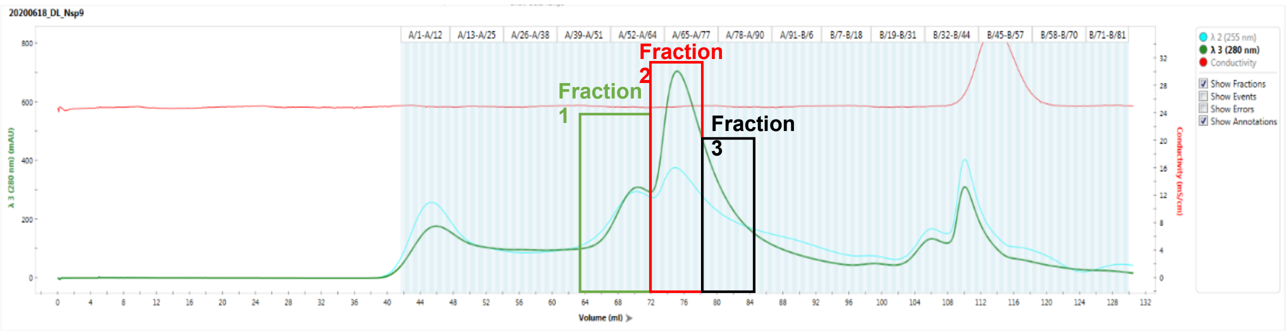

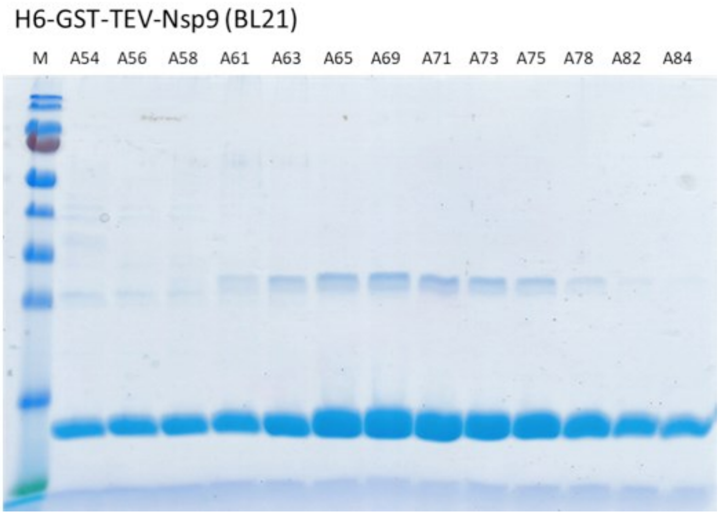

SEC profile of TEV-cleaved His_6-GST-fl_nsp9 (HiLoad 16/600 SD 75, GE Healthcare) **and SDS gel of corresponding fractions.** (Ladder: PageRuler™ prestained, Thermo Fischer)
Main peak (fraction 2 - corresponding to SEC fractions A 61 to A73) was subsequently used for NMR.

SI8: nsp10

Table 1: General Information

**1**	**Protein Name (according to NCBI Reference Sequence NC_045512.2)**
	ORF1a and ORF1ab; nsp10
**2**	**Region/Name/Further Specification**
	nsp10
**3**	**Sequence of fl protein (according to NCBI Reference Sequence NC_045512.2)**
	AGNATEVPANSTVLSFCAFAVDAAKAYKDYLASGGQPITNCVKMLCTHTGTGQAITVTPEAN MDQESFGGASCCLYCRCHIDHPNPKGFCDLKGKYVQIPTTCANDPVGFTLKNTVCTVCGMWK GYGCSCDQLREPMLQ
**4**	**Protein boundaries of expressed construct (according to NCBI Reference Sequence NC_045512.2)**
	aa 1-139 (fl nsp10)
**5**	**Ratio for construct design**
	fl protein
**6**	**Sequence homology (to SCoV)**
	Identity: 97%; similarity: 99%
**7**	**Published structures (SCoV2 or homologue variants)**
	SCoV: PDB 5C8S, 5NFY, 2FYG, 2XYQ, 2XYV, 2XYV<br>SCoV2: PDB 6W4H, 6W61, 7JYY, 7C2I, 7BQ7, 2G9T
**8**	**(Published) assignment (SCoV2 or homologue variants)**
	SCoV2: BMRB 50392

Table 2: Protein Expression

**1**	**Expression vector**
	pET21b(+) (GenScript)
**2**	**Purification-/Solubility-Tag**
	N-terminal His_6
**3**	**Cleavage Site**
	-
**4**	**Molecular weight / Extinction coefficient / pI - of protein**
	16.24 kDa / 12,950 M^{-1} cm^{-1} / 6.72
**5**	**Comments on sequence of expressed construct**
	N-terminal “MGSDKIHHHHHH” twelve artificial residues due to construct design
**6**	**Used expression strain**

	E. coli T7 Express
**7**	**Cultivation medium**
	LB / M9 (uniformly ^{15}N or $^{13}C,^{15}N$-labelled)
**8**	**Induction system**
	IPTG inducible T7 promoter
**9**	**Induction of protein expression**
	0.5 mM IPTG at OD_{600} 0.6-0.7 (addition of 50 µM $ZnCl_2$)
**10**	**Cultivation temperature and time**
	18-20°C for 16-18 h

Table 3: Protein Purification

**1**	**Buffer List**
A	25 mM Tris-HCl (pH 8.0), 300 mM NaCl, 5 mM imidazole, 10 mM bME (cell disruption / IMAC)
B	50 mM NaPi (pH 7.5), 50 mM NaCl, 5 mM DTT (SEC / final NMR buffer)
**2**	**Purification steps (with corresponding buffer(s) and incubation times)**
A	Cell disruption in buffer **1A** (plus one tablet of EDTA free protease inhibitor cocktail (Merck) and addition of 50 µM $ZnCl_2$) by microfluidization.
B	IMAC (HisTrap HP (GE Healthcare), ÄKTA start (GE Healthcare)), elution with imidazole gradient up to 500 mM in buffer **1A.**
C	SEC (HiLoad 26/600 SD 75 pg (GE Healthcare), ÄKTApurifier (GE Healthcare)) in buffer **1B** (elution volume 175-225 mL).
D	NMR sample preparation in buffer **1B.**

Table 4: Final sample

**1**	**Yield**
	25 mg/L ^{15}N-M9 medium, 15 mg/L $^{13}C,^{15}N$-M9 medium
**2**	**Stability**
	Stable throughout measurement (6 days, 298 K). No significant precipitation or degradation observed after storage at -80°C for 2 months.
**3**	**Comment on applicability**
	Suitable for NMR structure determination, fragment screening, interaction studies.

Additional information

**Constructs**	**Conditions**	**Comments**
aa 1-139 (fl nsp10); His_6 (pMCSG53 (BEI Resources, cat.	**IMAC-buffer:** 50 mM Tris-HCl (pH 9.0), 0.5 M NaCl, 10 mM bME,	Yields 30-40 mg/L 2xTY medium. Can be flash-frozen in liquid

NR-52425)), TEV cleavage site, N-terminal "SNM" three artificial residues.	2 mM $MgCl_2$, 0.1% (v/v) Triton X-100, 5-10% (v/v) glycerol, 50 mM imidazole.<br>**SEC-buffer:** 20 mM HEPES (pH 8.5), 0.5 M NaCl, 10 mM bME, 2 mM $MgCl_2$, 5% (v/v) glycerol, 20 mM imidazole.	nitrogen and stored at 20°C, used for nsp14 and nsp16 stabilization at 1:1 molar ratios.

SI9: nsp13

Table 1: General Information

**1**	**Protein Name (according to NCBI Reference Sequence NC_045512.2)**
	ORF1ab; nsp13
**2**	**Region/Name/Further Specification**
	NTPase / helicase domain / RNA 5'-triphosphatase
**3**	**Sequence of fl protein (according to NCBI Reference Sequence NC_045512.2)**
	AVGACVLCNSQTSLRCGACIRRPFLCCKCCYDHVISTSHKLVLSVNPYVCNAPGCDVTDVTQL YLGGMSYYCKSHKPPISFPLCANGQVFGLYKNTCVGSDNVTDFNAIATCDWTNAGDYILANTC TERLKLFAAETLKATEETFKLSYGIATVREVLSDRELHLSWEVGKPRPPLNRNYVFTGYRVTKN SKVQIGEYTFEKGDYGDAVVYRGTTTYKLNVGDYFVLTSHTVMPLSAPTLVPQEHYVRITGLY PTLNISDEFSSNVANYQKVGMQKYSTLQGPPGTGKSHFAIGLALYYPSARIVYTACSHAAVDAL CEKALKYLPIDKCSRIIPARARVECFDKFKVNSTLEQYVFCTVNALPETTADIVVFDEISMATNY DLSVVNARLRAKHYVYIGDPAQLPAPRTLLTKGTLEPEYFNSVCRLMKTIGPDMFLGTCRRCPA EIVDTVSALVYDNKLKAHKDKSAQCFKMFYKGVITHDVSSAINRPQIGVVREFLTRNPAWRKA VFISPYNSQNAVASKILGLPTQTVDSSQGSEYDYVIFTQTTETAHSCNVNRFNVAITRAKVGILCI MSDRDLYDKLQFTSLEIPRRNVATLQ
**4**	**Protein boundaries of expressed construct (according to NCBI Reference Sequence NC_045512.2)**
	1-601 aa (fl nsp13)
**5**	**Ratio for construct design**
	fl protein
**6**	**Sequence homology (to SCoV)**
	Identity: 99.8%; similarity: 100%
**7**	**Published structures (SCoV2 or homologue variants)**
	SCoV2: PDB 6ZSL, 6JYT, 6XEZ
**8**	**(Published) assignment (SCoV2 or homologue variants)**
	-

Table 2: Protein Expression

**1**	**Expression vector**
	pE-SUMO (LifeSensors)
**2**	**Purification-/Solubility-Tag**
	N-terminal His_6-SUMO
**3**	**Cleavage Site**
	Ulp1
**4**	**Molecular weight / Extinction coefficient / pI - of cleaved protein**

	66.85 kDa / 67,160 M^{-1} cm^{-1} / 8.66
**5**	**Comments on sequence of expressed construct**
	No artificial residues due to Ulp1-cleavage and construct design.
**6**	**Used expression strain**
	E. coli BL21 (DE3)
**7**	**Cultivation medium**
	LB / M9 (uniformly ^{15}N-labelled)
**8**	**Induction system**
	IPTG inducible T7 promoter
**9**	**Induction of protein expression**
	0.2 mM IPTG at OD_{600} 0.6-0.7 (addition of 50 µM $ZnCl_2$)
**10**	**Cultivation temperature and time**
	18-20°C for 16-18 h

Table 3: Protein Purification

**1**	**Buffer List**
A	25 mM Tris (pH 8.0), 300 mM NaCl, 5 mM imidazole, 5% (v/v) glycerol, 10 mM bME (cell disruption / IMAC).
B	20 mM BisTris (pH 7.0), 150 mM NaCl, 2 mM TCEP-HCl (SEC/ final NMR buffer).
**2**	**Purification steps (with corresponding buffer(s) and incubation times)**
A	Cell disruption in buffer **1A** (plus one tablet of EDTA free protease inhibitor cocktail (Merck) and addition of 50 µM $ZnCl_2$) by microfluidization.
B	IMAC (HisTrap HP (GE Healthcare), ÄKTA start (GE Healthcare)), elution with imidazole gradient up to 500 mM in buffer **1A**.
C	SEC (HiLoad 26/600 SD 200 pg (GE Healthcare), ÄKTApurifier (GE Healthcare)) in buffer **1B** (elution volume 210-240 mL).
D	NMR sample preparation in buffer **1B.**

Table 4: Final sample

**1**	**Yield**
	0.5 mg/L ^{15}N-M9 medium
**2**	**Stability**
	Aggregation at > 20 µM under these conditions.
**3**	**Comment on applicability**
	Not suitable for NMR experiments.

Additional information

**Constructs**	**Conditions**	**Comments**
aa 1-601 (fl nsp13); His_6 (pET28a(+) (GenScript)), TEV-cleavage site, N-terminal "GHM" three artificial residues.	Native (as above)	Weak expression, instable protein.

SI10: nsp14

Table 1: General Information

1	**Protein Name (according to NCBI Reference Sequence NC_045512.2)**
	ORF1ab; nsp14
2	**Region/Name/Further Specification**
	nsp14 / 3'-to-5' exonuclease / guanine N7-methyltransferae (MTase)
3	**Sequence of fl protein (according to NCBI Reference Sequence NC_045512.2)**
	AENVTGLFKDCSKVITGLHPTQAPTHLSVDTKFKTEGLCVDIPGIPKDMTYRRLISMMGFKMNY QVNGYPNMFITREEAIRHVRAWIGFDVEGCHATREAVGTNLPLQLGFSTGVNLVAVPTGYVDT PNNTDFSRVSAKPPPG
4	**Protein boundaries of expressed construct (according to NCBI Reference Sequence NC_045512.2)**
**fl**	aa 1-527 (fl nsp14)
**MTase**	aa 288-527 (MTase domain)
5	**Ratio for construct design**
**fl**	fl protein
**MTase**	In analogy to SCoV structure (PDB 5C8U)
6	**Sequence homology (to SCoV)**
**fl**	Identity: 95%; similarity: 99%
**MTase**	Identity: 95%, similarity: 97%
7	**Published structures (SCoV2 or homologue variants)**
	SCoV: PDB 5C8U, 5C8S, 5C8T, 5NFY
8	**(Published) assignment (SCoV2 or homologue variants)**
	-

Table 2: Protein Expression

1	**Expression vector**
**fl**	pRSF-Duet1 (Novagen)
**MTase**	pET28a (Novagen)
2	**Purification-/Solubility-Tag**
**fl**	N-terminal His_6
**MTase**	N-terminal His_6
3	**Cleavage Site**
**fl**	TEV

**MTase**	Thrombin
**4**	**Molecular weight / Extinction coefficient / pI - of cleaved protein**
**fl**	60.01 kDa / 91,660 M^{-1} cm^{-1} / 7.79
**MTase**	27.82 kDa / 48,970 M^{-1} cm^{-1} / 7.19
**5**	**Comments on sequence of expressed construct**
**fl**	N-terminal "GSM" three artificial residues due to construct design.
**MTase**	N-terminal "GSHM" four artificial residues due to construct design.
**6**	**Used expression strain**
	E. coli BL21 (DE3)
**7**	**Cultivation medium**
	2xTY for protein production, LB for transformation and maintenance
**8**	**Induction system**
	IPTG inducible T7 promoter
**9**	**Induction of protein expression**
	1 mM IPTG at OD_{540} 0.5-0.6
**10**	**Cultivation temperature and time**
	20°C for 18-20 h

Table 3: Protein Purification (fl nsp14 and nsp14 MTase)

**1**	**Buffer List**
A	50 mM Tris-HCl (pH 9.0), 0.5 M NaCl, 10 mM bME, 2 mM $MgCl_2$, 0.1% (v/v) Triton X-100, 10% (v/v) glycerol, 50 mM imidazole (cell disruption).
B	50 mM Tris-HCl (pH 9.0), 0.5 M NaCl, 10 mM bME, 2 mM $MgCl_2$, 5% (v/v) glycerol, 50 mM imidazole (IMAC).
C	50 mM Tris-HCl (pH 9.0), 0.5 M NaCl, 10 mM bME, 2 mM $MgCl_2$, 5% (v/v) glycerol, 1 M imidazole (IMAC).
D	20 mM HEPES (pH 8.5), 0.5 M NaCl, 10 mM bME, 2 mM $MgCl_2$, 5% (v/v) glycerol, 20 mM imidazole (SEC).
E	20 mM potassium phosphate (pH 8.0), 0.25 M KCl (Screening).
**2**	**Purification steps (with corresponding buffer(s) and incubation times)**
A	Cell disruption in buffer **1A** by sonication in pulse mode (0.5 s on /0.5 s off) for 10 min.
B	IMAC (gravity flow or batch Ni^{2+}-NTA) (GE Healthcare), washing with buffer **1B**, elution with **1C**.
C-**fl**	[Optional] Overnight incubation with TEV protease at 4°C. The ratio was 1 mg of TEV protease per 20-40 mg of nsp14 protein.

C-**MTase**	[Optional] Overnight incubation with thrombin protease at 4°C. The ratio was 1-2 U of thrombin protease per 3-4 mg of MTase nsp14 protein.
D	SEC on SD 200 16/600 column (GE Healthcare) in buffer **1D** (elution volume 75-95 mL).
E-**fl**	[Optional] Separation of TEV protease and uncleaved nsp14 material with IMAC, collection of flow through in buffer **1D**.
E-**MTase**	[Optional] Separation of thrombin protease and uncleaved MTase nsp14 material with IMAC, collection of flow through in buffer **1D**.
F	For fragment screening the buffer is exchanged to **1E**.
G	[Optional] If higher concentrations or increased stability of fl nsp14 is desired, nsp10 should be added at 1:1 molar ratio.

Table 4: Final sample

**1**	**Yield**
**fl**	6 mg/L 2xTY medium
**MTase**	~ 10 mg/L 2xTY medium
**1b**	**A260/280 ratio**
fl	0.6
**MTase**	0.6
**2**	**Stability**
**fl**	The fl nsp14 construct tends to be unstable at concentrations above 3 mg/mL without reducing agent (TCEP-HCl or bME). Unstable at 4°C longer than one week. Freezing is not advisable; storage in 50% (v/v) glycerol at -20°C is preferable.
**MTase**	The MTase construct is even more unstable, and requires the presence of reducing agent (TCEP-HCl or bME) and NaCl at least in 400 mM concentration.
**3**	**Comment on applicability**
	Suitable for fragment screening and enzymatic activity assays.

Additional information

**Constructs**	**Conditions**	**Comments**
Fl nsp14; His_6 (pETDuet (GenScript)), no cleavage site, N-terminal "MGSSHHHHHHSQDP" 14 artificial residues.	**IMAC-buffer:** 25 mM Tris/HCl (pH 8.5), 300 mM NaCl, 5 mM imidazole, 10 mM bME, 5% (v/v) glycerol.<br>**SEC-buffer:** 25 mM Tris/HCl (pH 8.5), 300 mM NaCl, 5 mM DTT, 5% (v/v) glycerol	Yields 14 mg/L ^{15}N-M9 medium. Tendency to aggregate.

SI11: nsp15

Table 1: General Information

**1**	**Protein Name (according to NCBI Reference Sequence NC_045512.2)**
	ORF1ab; nsp15
**2**	**Region/Name/Further Specification**
	nsp15 / NendoU / Endonucleasee
**3**	**Sequence of fl protein (according to NCBI Reference Sequence NC_045512.2)**
	SLENVAFNVVNKGHFDGQQGEVPVSIINNTVYTKVDGVDVELFENKTTLPVNVAFELWAKRNI<br>KPVPEVKILNNLGVDIAANTVIWDYKRDAPAHISTIGVCSMTDIAKKPTETICAPLTVFFDGRVD<br>GQVDLFRNARNGVLITEGSVKGLQPSVGPKQASLNGVTLIGEAVKTQFNYYKKVDGVVQQLPE<br>TYFTQSRNLQEFKPRSQMEIDFLELAMDEFIERYKLEGYAFEHIVYGDFSHSQLGGLHLLIGLAK<br>RFKESPFELEDFIPMDSTVKNYFITDAQTGSSKCVCSVIDLLLDDFVEIIKSQDLSVVSKVVKVTI<br>DYTEISFMLWCKDGHVETFYPKLQ
**4**	**Protein boundaries of expressed construct (according to NCBI Reference Sequence NC_045512.2)**
	aa 1-346 (fl nsp15)
**5**	**Ratio for construct design**
	fl protein
**6**	**Sequence homology (to SCoV)**
	Identity: 89%; similarity: 98%
**7**	**Published structures (SCoV2 or homologue variants)**
	SCoV: PDB 2H85<br>SCoV2: PDB 6W01
**8**	**(Published) assignment (SCoV2 or homologue variants)**
	-

Table 2: Protein Expression

**1**	**Expression vector**
	pET28a(+) (GenScript)
**2**	**Purification-/Solubility-Tag**
	N-terminal His_6
**3**	**Cleavage Site**
	TEV
**4**	**Molecular weight / Extinction coefficient / pI - of cleaved protein**
	39.14 kDa / 32,890 M^{-1} cm^{-1} / 5.12
**5**	**Comments on sequence of expressed construct**

	N-terminal "GHM" three artificial residues due to TEV-cleavage and construct design
**6**	**Used expression strain**
	E. coli BL21 (DE3)
**7**	**Cultivation medium**
	LB / M9 (uniformly ^{15}N-labelled)
**8**	**Induction system**
	IPTG inducible T7 promoter
**9**	**Induction of protein expression**
	0.2 mM IPTG at OD_{600} 0.6-0.7
**10**	**Cultivation temperature and time**
	18-20°C for 16-18 h

Table 3: Protein Purification

**1**	**Buffer List**
A	25 mM Tris-HCl (pH 8.0), 300 mM NaCl, 5 mM imidazole, 5% (v/v) glycerol, 10 mM bME (cell disruption / IMAC).
B	25 mM NaPi (pH 7.5), 300 mM NaCl, 2 mM TCEP-HCl (SEC/ final NMR buffer).
**2**	**Purification steps (with corresponding buffer(s) and incubation times)**
A	Cell disruption in buffer **1A** (plus one tablet of EDTA free protease inhibitor cocktail (Merck)) by microfluidization.
B	IMAC (HisTrap HP (GE Healthcare), ÄKTA start (GE Healthcare)), elution with imidazole gradient up to 500 mM in buffer **1A**.
C	SEC (HiLoad 26/600 SD 200 pg (GE Healthcare), ÄKTApurifier (GE Healthcare)) in buffer **1B** (elution volume 200-260 mL).
D	NMR sample preparation in buffer **1B**.

Table 4: Final sample

**1**	**Yield**
	5 mg/L ^{15}N-M9 medium
**2**	**Stability**
	Tendency to aggregate at rt.
**3**	**Comment on applicability**
	Suitable for fragment screening and interaction studies.

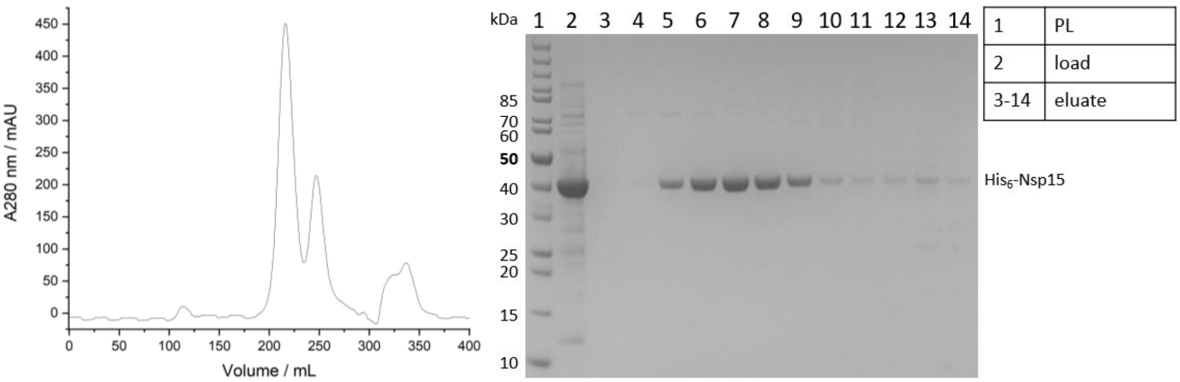

Analytical SEC of nsp15. Protein was eluted from 200-260 mL (left panel) with corresponding SDS-PAGE of SEC with fractions analyzed from 190-260 mL (right panel).

SI12: nsp16

Table 1: General Information

1	**Protein Name (according to NCBI Reference Sequence NC_045512.2)**
	ORF1ab; nsp16
2	**Region/Name/Further Specification**
	nsp16 / 2'-O-ribose methyltransferase (2’-O-MTase)
3	**Sequence of fl protein (according to NCBI Reference Sequence NC_045512.2)**
	SSQAWQPGVAMPNLYKMQRMLLEKCDLQNYGDSATLPKGIMMNVAKYTQLCQYLNTLTLAV<br>PYNMRVIHFGAGSDKGVAPGTAVLRQWLPTGTLLVDSDLNDFVSDADSTLIGDCATVHTANK<br>WDLIISDMYDPKTKNVTKENDSKEGFFTYICGFIQQKLALGGSVAIKITEHSWNADLYKLMGHF<br>AWWTAFVTNVNASSSEAFLIGCNYLGKPREQIDGYVMHANYIFWRNTNPIQLSSYSLFDMSKFP<br>LKLRGTAVMSLKEGQINDMILSLLSKGRLIIRENNRVVISSDVLVNN
4	**Protein boundaries of expressed construct (according to NCBI Reference Sequence NC_045512.2)**
	aa 1-298 (fl nsp16)
5	**Ratio for construct design**
	Based on fl annotation boundaries of YP_009725311.1 protein entry in NC_045512.2.
6	**Sequence homology (to SCoV)**
	Identity: 93%; similarity: 99%
7	**Published structures (SCoV2 or homologue variants)**
	SCoV: PDB 3R24, 2XYR, 2XYQ<br>SCoV2: PDB 7JYY, 6W4H, 6YZ1, 7BQ7, 7C2I, 6W61
8	**(Published) assignment (SCoV2 or homologue variants)**
	-

Table 2: Protein Expression

1	**Expression vector**
	pRSF-Duet1 (Novagen)
2	**Purification-/Solubility-Tag**
	N-terminal His_6
3	**Cleavage Site**
	TEV
4	**Molecular weight / Extinction coefficient / pI - of cleaved protein**
	33.67 kDa / 55,790 M^{-1} cm^{-1}/ 7.76
5	**Comments on sequence of expressed construct**
	N-terminal „GSMA" - four artificial residues due to TEV-cleavage and construct design.

**6**	**Used expression strain**
	E. coli BL21(DE3)
**7**	**Cultivation medium**
	2xTY
**8**	**Induction system**
	IPTG inducible T7 promoter
**9**	**Induction of protein expression**
	1 mM IPTG at OD_{540} 0.5-0.6
**10**	**Cultivation temperature and time**
	20°C for 18-20 h

Table 3: Protein Purification

**1**	**Buffer List**
A	50 mM Tris-HCl (pH 9.0), 500 mM NaCl, 10 mM bME, 2 mM $MgCl_2$, 0.1% (v/v) Triton X-100, 10% (v/v) glycerol, 50 mM imidazole (cell disruption).
B	50 mM Tris-HCl (pH 9.0), 500 mM NaCl, 10 mM bME, 2 mM $MgCl_2$, 5% (v/v) glycerol, 50 mM imidazole (IMAC).
C	50 mM Tris-HCl (pH 9.0), 500 mM NaCl, 10 mM bME, 2 mM $MgCl_2$, 5% (v/v) glycerol, 1 M imidazole (IMAC).
D	20 mM HEPES (pH 8.5), 500 mM NaCl, 10 mM bME, 2 mM $MgCl_2$, 5% (v/v) glycerol, 20 mM imidazole (SEC).
E	20 mM KPi (pH 8.0), 200 mM KCl, 1 mM $MgCl_2$, 2 mM DTT (Screening).
**2**	**Purification steps (with corresponding buffer(s) and incubation times)**
A	Cell disruption in buffer **1A** by sonication in pulse mode (0.5 s on /0.5 s off) for 10 min.
B	IMAC (gravity flow or batch Ni^{2+}-NTA) (GE Healthcare), washing with buffer **1B**, elution with **1C**.
C	[Optional] Overnight incubation with TEV protease at 4°C. The ratio was 1 mg of TEV protease per 20-40 mg of nsp16 protein.
D	SEC on SD 200 16/600 column (GE Healthcare) in buffer **1D** (elution volume 90-100 mL).
E	[Optional] Separation of TEV protease and uncleaved nsp16 material with IMAC, collection of flow through in buffer **1D**.
F	nsp10 is added at 1:1 molar ratio – necessary for stability and activity.
G	For fragment screening the buffer is exchanged to **1E**.

Table 4: Final sample

**1**	**Yield**
	~ 10-15 mg/L 2xTY medium.

1b	**A260/280 ratio**
	0.55
**2**	**Stability**
	Extremely unstable in non-reducing conditions, presence of reducing agents is essential. Presence of 5% (v/v) glycerol is also desirable for increased stability. Can be flash-frozen in liquid nitrogen and stored at -20°C.
**3**	**Comment on applicability**
	Suitable for fragment screening.

Additional information

**Constructs**	**Conditions**	**Comments**
Fl nsp16; His_6 (pMCSG53 (BEI Resources, cat. NR-52427)), TEV-cleavage site, N-terminal „SNM" three artificial residues.	As above	~ 5 mg/L 2xTY medium). Purity and stability is comparable to the “GSMA” construct above.

SI13: ORF3a

Table 1: General Information

1	**Protein Name (according to NCBI Reference Sequence NC_045512.2)**
	ORF3a
2	**Region/Name/Further Specification**
3	**Sequence of fl protein (according to NCBI Reference Sequence NC_045512.2)**
	MDLFMRIFTIGTVTLKQGEIKDATPSDFVRATATIPIQASLPFGWLIVGVALLAVFQSASKIITLK KRWQLALSKGVHFVCNLLLLFVTVYSHLLLVAAGLEAPFLYLYALVYFLQSINFVRIIMRLWLC WKCRSKNPLLYDANYFLCWHTNCYDYCIPYNSVTSSIVITSGDGTTSPISEHDYQIGGYTEKWE SGVKDCVVLHSYFTSDYYQLYSTQLSTDTGVEHVTFFIYNKIVDEPEEHVQIHTIDGSSGVVNPV MEPIYDEPTTTTSVPL
4	**Protein boundaries of expressed construct (according to NCBI Reference Sequence NC_045512.2)**
	aa 1-275 (fl ORF3a)
5	**Ratio for construct design**
	fl protein
6	**Sequence homology (to SCoV)**
	Identity: 72.4%; similarity: 90.2%
7	**Published structures (SCoV2 or homologue variants)**
	SCoV2: PDB 6XDC
8	**(Published) assignment (SCoV2 or homologue variants)**
	**-**

Table 2: Cell-free Protein Synthesis

1	**Expression vector**
	pEU-E01-MCS (Cell-Free Sciences)
2	**Purification-/Solubility-Tag**
	C-terminal Strep tag II (WSHPQFEK)
3	**Cleavage Site**
	-
4	**Molecular weight / Extinction coefficient / pI - of protein**
	32.32 kDa / 64,205 M^{-1} cm^{-1} / 5.67
5	**Comments on sequence of expressed construct**
	C-terminal “SAWSHPQFEK” ten artificial residues due to construct design.

**6**	**Feeding buffer**
	30 mM HEPES-KOH (pH 7.6), 100 mM potassium acetate, 2.7 mM magnesium acetate, 16 mM creatine phosphate, 0.4 mM spermidine, 1.2 mM ATP, 0.25 mM GTP, 4 mM DTT and 6 mM (average concentration) amino acid mix and 0.05% (w/v) Brij-58.
**7**	**Translation mix**
	50% (v/v) mRNA, 50% (v/v) home-made WGE, 40 µg/mL creatine kinase, and 6 mM (average concentration) amino acid mix 0.05% (w/v) Brij-58.
**8**	**Protein synthesis temperature and time**
	22°C for 16 h without agitation (bilayer method).

Table 3: Protein Purification

**1**	**Buffer List**
A	100 mM Tris-HCl (pH 8.0), 150 mM NaCl, 1 mM EDTA, 0.1% (w/v) DDM (wash buffer).
B	100 mM Tris-HCl (pH 8.0), 150 mM NaCl, 1 mM EDTA, 2.5 mM desthiobiotin, and 0.1% (w/v) DDM (elution buffer).
**2**	**Purification steps (with corresponding buffer(s) and incubation times)**
A	Harvest total CFS.
B	Incubate with benzonase for 30 min on a wheel, at rt.
C	Centrifuge for 30 min at 20,000 g, 4°C.
D	Harvest the soluble fraction (SN).
E	Equilibrate the Strep-Tactin column (IBA Lifesciences) with 2 CV of **1A** (all steps performed on the bench by gravity).
F	Load SN onto the column.
G	Wash the column with 5 CV of **1A**.
H	Elute the protein of interest with **1B**.

Table 4: Final sample

**1**	**Yield**
	0.6 mg/mL WGE
**1b**	**A260/280 ratio**
	1.08
**2**	**Stability**
	Stable at 4°C for at least 2 weeks.
**3**	**Comment on applicability**
	ORF3a-ST is eluted with small heat shock protein (SHSP, 18 kDa) from wheat.

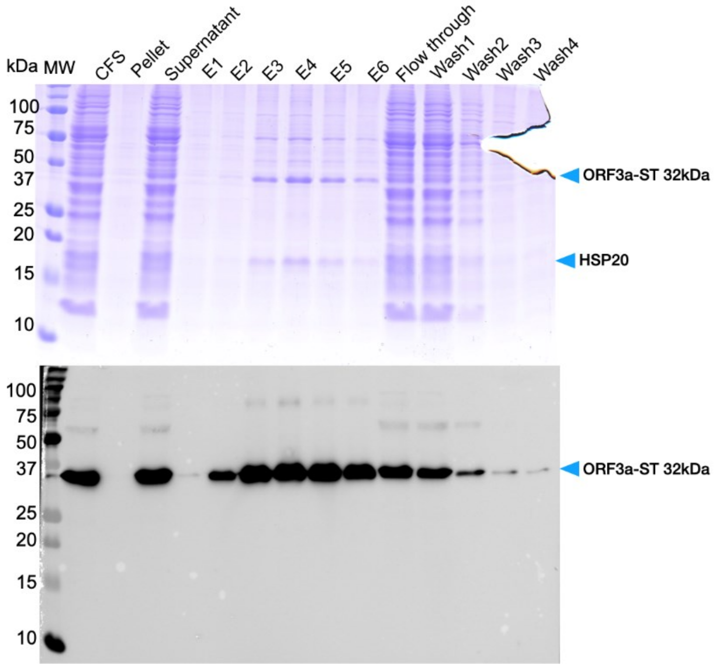

WG-CFPS in the presence of detergent, and Strep-tag purification of ORF3a. SDS-PAGE (upper panel) and WB (lower panel).

SI14: ORF4 (Envelope (E) protein)

Table 1: General Information

1	**Protein Name (according to NCBI Reference Sequence NC_045512.2)**
	ORF 4; Envelope (E) protein
2	**Region/Name/Further Specification**
	E protein
3	**Sequence of fl protein (according to NCBI Reference Sequence NC_045512.2)**
	MYSFVSEETGTLIVNSVLLFLAFVVFLLVTLAILTALRLCAYCCNIVNVSLVKPSFYVYSRVKNL NSSRVPDLLV
4	**Protein boundaries of expressed construct (according to NCBI Reference Sequence NC_045512.2)**
	aa 1-75 (fl ORF4)
5	**Ratio for construct design**
	fl protein
6	**Sequence homology (to SCoV)**
	Identity: 94.7%; similarity: 97.4%
7	**Published structures (SCoV2 or homologue variants)**
	SCoV: PDB 5X29
8	**(Published) assignment (SCoV2 or homologue variants)**
	SCoV: BMRB 36049

Table 2: Cell-free Protein Synthesis

1	**Expression vector**
	pEU-E01-MCS (Cell-free Sciences)
2	**Purification-/Solubility-Tag**
	C-terminal Strep tag II (WSHPQFEK)
3	**Cleavage Site**
	-
4	**Molecular weight / Extinction coefficient / pI - of protein**
	9.56 kDa / 11,460 M^{-1} cm^{-1} / 8.55
5	**Comments on sequence of expressed construct**
	C-terminal “SAWSHPQFEK” ten artificial residues due to construct design.
6	**Feeding buffer**

	30 mM HEPES-KOH (pH 7.6), 100 mM potassium acetate, 2.7 mM magnesium acetate, 16 mM creatine phosphate, 0.4 mM spermidine, 1.2 mM ATP, 0.25 mM GTP, 4 mM DTT and 6 mM (average concentration) amino acid mix and 0.05% (w/v) Brij-58.
**7**	**Translation mix**
	50% (v/v) mRNA, 50% (v/v) home-made WGE, 40 µg/mL creatine kinase, and 6 mM (average concentration) amino acid mix 0.05% (w/v) Brij-58.
**8**	**Protein synthesis temperature and time**
	22°C for 16 h without agitation (bilayer method).

Table 3: Protein Purification

**1**	**Buffer List**
A	100 mM Tris-HCl (pH 8.0), 150 mM NaCl, 1 mM EDTA, 0.1% (w/v) DDM (wash buffer).
B	100 mM Tris-HCl (pH 8.0), 150 mM NaCl, 1 mM EDTA, 2.5 mM desthiobiotin, and 0.1% (w/v) DDM (elution buffer).
**2**	**Purification steps (with corresponding buffer(s) and incubation times)**
A	Harvest total CFS.
B	Incubate with benzonase for 30 min on a wheel, at rt.
C	Centrifuge for 30 min at 20,000 g, 4°C.
D	Harvest the soluble fraction (SN).
E	Equilibrate the Strep-Tactin column (IBA Lifesciences) with 2 CV of **1A** (all steps performed on the bench by gravity).
F	Load SN onto the column.
G	Wash the column with 5 CV of **1A**.
H	Elute the protein of interest with **1B**.

Table 4: Final sample

**1**	**Yield**
	0.45 mg/mL WGE
**1b**	**A260/280 ratio**
	1.52
**2**	**Stability**
	Stable at least a few days at rt.
**3**	**Comment on applicability**
	E protein cannot be sedimented and is thus not directly available for solid-state NMR. Lipid reconstitution will be needed.

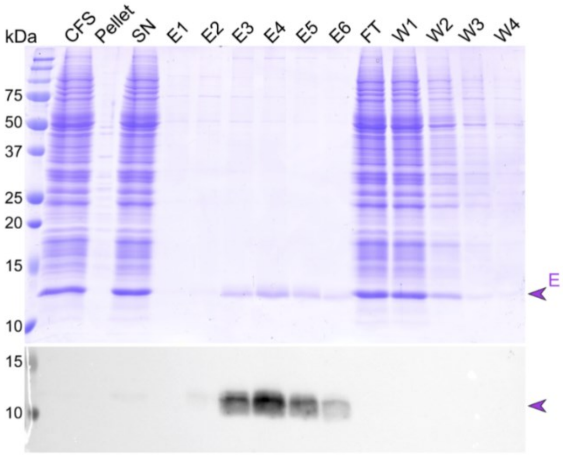

WG-CFPS in the presence of detergent, and Strep-tag purification of E (ORF4). SDS-PAGE (upper panel) and WB (lower panel).

SI15: ORF5 (M protein)

Table 1: General Information

1	**Protein Name (according to NCBI Reference Sequence NC_045512.2)**
	ORF5; Membrane glycoprotein (M)
2	**Region/Name/Further Specification**
	M protein
3	**Sequence of fl protein (according to NCBI Reference Sequence NC_045512.2)**
	MADSNGTITVEELKKLLEQWNLVIGFLFLTWICLLQFAYANRNRFLYIIKLIFLWLLWPVTLACF VLAAVYRINWITGGIAIAMACLVGLMWLSYFIASFRLFARTRSMWSFNPETNILLNVPLHGTILT RPLLESELVIGAVILRGHLRIAGHHLGRCDIKDLPKEITVATSRTLSYYKLGASQRVAGDSGFAA YSRYRIGNYKLNTDHSSSSDNIALLVQ
4	**Protein boundaries of expressed construct (according to NCBI Reference Sequence NC_045512.2)**
	aa 1-222 (fl ORF5)
5	**Ratio for construct design**
	fl protein
6	**Sequence homology (to SCoV)**
	Identity: 90.5%; similarity: 98.2%
7	**Published structures (SCoV2 or homologue variants)**
	-
8	**(Published) assignment (SCoV2 or homologue variants)**
	-

Table 2: Cell-free Protein Synthesis

1	**Expression vector**
	pEU-E01-MCS (Cell-Free Sciences)
2	**Purification-/Solubility-Tag**
	C-terminal Strep tag II (WSHPQFEK)
3	**Cleavage Site**
	-
4	**Molecular weight / Extinction coefficient / pI - of protein**
	26.35 kDa / 57,660 M^{-1} cm^{-1} / 9.48
5	**Comments on sequence of expressed construct**
	C-terminal “SAWSHPQFEK” ten artificial residues due to construct design.

**6**	**Feeding buffer**
	30 mM HEPES-KOH (pH 7.6), 100 mM potassium acetate, 2.7 mM magnesium acetate, 16 mM creatine phosphate, 0.4 mM spermidine, 1.2 mM ATP, 0.25 mM GTP, 4 mM DTT, 6 mM (average concentration) amino acid mix, and 0.05% (w/v) Brij-58.
**7**	**Translation mix**
	50% (v/v) mRNA, 50% (v/v) home-made WGE, 40 µg/mL creatine kinase, 6 mM (average concentration), and amino acid mix 0.05% (w/v) Brij-58.
**8**	**Protein synthesis temperature and time**
	22°C for 16 h without agitation (bilayer method).

Table 3: Protein Purification

**1**	**Buffer List**
A	100 mM Tris-HCl (pH 8.0), 150 mM NaCl, 1 mM EDTA, 0.1% (w/v) DDM (wash buffer).
B	100 mM Tris-HCl (pH 8.0), 150 mM NaCl, 1 mM EDTA, 2.5 mM desthiobiotin, and 0.1% (w/v) DDM (elution buffer).
**2**	**Purification steps (with corresponding buffer(s) and incubation times)**
A	Harvest total CFS.
B	Incubate with benzonase for 30 min on a wheel, at rt.
C	Centrifuge for 30 min at 20,000 g, 4°C.
D	Harvest the soluble fraction (SN).
E	Equilibrate the Strep-Tactin column (IBA Lifesciences) with 2 CV of **1A** (all steps performed on the bench by gravity).
F	Load SN onto the column.
G	Wash the column with 5 CV of **1A**.
H	Elute the protein of interest with **1B**.

Table 4: Final sample

**1**	**Yield**
	0.33 mg/mL WGE
**1b**	**A260/280 ratio**
	1.16
**2**	**Stability**
	Stable at 4°C for at least 2 weeks.
**3**	**Comment on applicability**
	Mprotein-ST (and ST-Mprot) is eluted with small heat shock protein (SHSP 18 kDa) from wheat.

Additional information

**Constructs**	**Conditions**	**Comments**
Fl ORF5; Strep tag II (pEU-E01-MCS (Cell-Free Sciences)); no cleavage site; N-terminal “WSHPQFEK“ eight artificial residues.	As above, but:<br>- Purification: 1 mM DTT was added in purification buffers 1A and 1B.<br>- Tab. 3.2B: 0.25% (w/v) DDM is added and incubated on the wheel for 1 h.<br>- Tab. 3.2C: 40,000 g for 40 min.<br>- Tab. 3.2E: added Strep beads for batch purification (200 µL 50% (w/v) suspension per well) and incubated on the wheel for 1.5 h.	Works as well with similar yield (0.39 mg/mL) and purity.

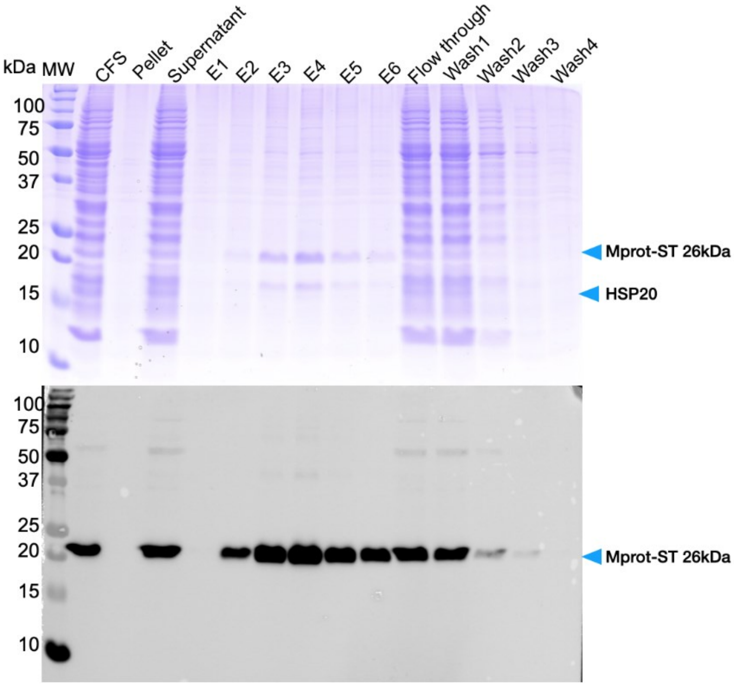

WG-CFPS in the presence of detergent, and Strep-tag purification of M (ORF5). SDS-PAGE (upper panel) and WB (lower panel).

SI16: ORF6

Table 1: General Information

1	**Protein Name (according to NCBI Reference Sequence NC_045512.2)**
	ORF6
2	**Region/Name/Further Specification**
3	**Sequence of fl protein (according to NCBI Reference Sequence NC_045512.2)**
	MFHLVDFQVTIAEILLIIMRTFKVSIWNLDYIINLIIKNLSKSLTENKYSQLDEEQPMEID
4	**Protein boundaries - amino acid numbering (according to NCBI Reference Sequence NC_045512.2):**
	aa 1-61 (fl ORF6)
5	**Ratio for construct design (detailed and comprehensible)**
	fl protein
6	**Sequence homology (to SCoV)**
	Identity: 68.9%; similarity: 93.4%
7	**Published structures (SCoV2 or homologue variants)**
	-
8	**(Published) assignment (SCoV2 or homologue variants)**
	-

Table 2: Cell-free Protein Synthesis

1	**Expression vector**
	pEU-E01-MCS (Cell-Free Sciences)
2	**Purification-/Solubility-Tag**
	C-terminal Strep tag II (WSHPQFEK)
3	**Cleavage Site**
	-
4	**Molecular weight / Extinction coefficient / pI - of cleaved protein**
	8470.85 kDa / 13,980 M^{-1} cm^{-1} / 4.89
5	**Comments on sequence of expressed construct**
	C-terminal "SAWSHPQFEK" ten artificial residues due to construct design.
6	**Feeding buffer**

	30 mM HEPES-KOH (pH 7.6), 100 mM potassium acetate, 2.7 mM magnesium acetate, 16 mM creatine phosphate, 0.4 mM spermidine, 1.2 mM ATP, 0.25 mM GTP, 4 mM DTT and 6 mM (average concentration) amino acid mix
**7**	**Translation mix**
	50% (v/v) mRNA, 50% (v/v) home-made WGE, 40 µg/mL creatine kinase, and 6 mM (average concentration) amino acid mix
**8**	**Protein synthesis temperature and time**
	22°C for 16 h without agitation (bilayer method).

Table 3: Protein Purification

**1**	**Buffer List**
A	20 mM NaPi (pH 6.5), 50 mM NaCl (wash buffer).
B	20 mM NaPi (pH 6.5), 50 mM NaCl, 2.5 mM desthiobiotin (elution buffer).
**2**	**Purification steps (with corresponding buffer(s) and incubation times)**
A	Harvest total CFS.
B	Incubate with benzonase for 30 min on a wheel, at rt.
C	Centrifuge for 30 min at 20,000 g, 4°C.
D	Harvest the soluble fraction (SN).
E	Equilibrate the Strep-Tactin column (IBA Lifesciences) with 2 CV of **1A** (all steps performed on the bench by gravity).
F	Load SN onto the column.
G	Wash the column with 5 CV of **1A**.
H	Elute the protein of interest with **1B**.

Table 4: Final sample

**1**	**Yield**
	0.27 mg/mL of WGE and total production of 875 µg for NMR samples
**1b**	**A260/280 ratio**
	1.36
**2**	**Stability**
	stable
**3**	**Comment on applicability**
	Positioning the Strep tag at the N-terminus abolished synthesis.

Additional information

Constructs	Conditions	Comments
Fl ORF6; Strep tag II (pEU-E01-MCS (Cell-Free Sciences)), no cleavage site, N-terminal "WSHPQFEK " eight artificial residues.		No expression observed.

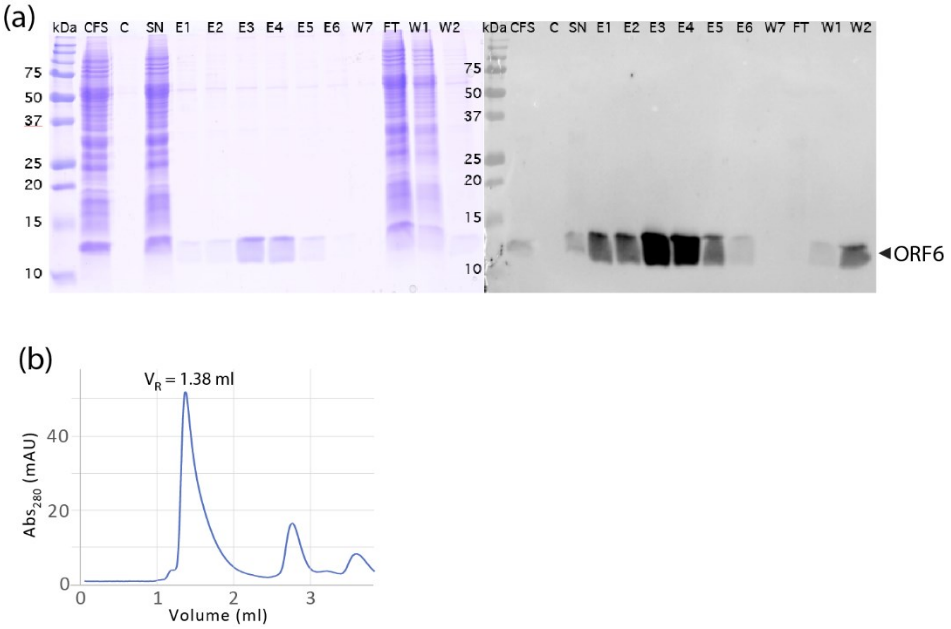

(a) WG-CFPS and Strep-tag purification of ORF6. SDS-PAGE (left panel) and WB (right panel). **(b) SEC profile of ORF6.**

SI17: ORF7a

Table 1: General Information

**1**	**Protein Name (according to NCBI Reference Sequence NC_045512.2)**
	ORF7a
**2**	**Region/Name/Further Specification**
	Ectodomain (ED)
**3**	**Sequence of fl protein (according to NCBI Reference Sequence NC_045512.2)**
	MKIILFLALITLATCELYHYQECVRGTTVLLKEPCSSGTYEGNSPFHPLADNKFALTCFSTQFAFA CPDGVKHVYQLRARSVSPKLFIRQEEVQELYSPIFLIVAAIVFITLCFTLKRKTE
**4**	**Protein boundaries of expressed construct (according to NCBI Reference Sequence NC_045512.2)**
	aa 16-81 (ectodomain of ORF7a)
**5**	**Ratio for construct design (detailed and comprehensible)**
	Only the ectodomain without signaling peptide. Transmembrane helix is also not included in the construct.
**6**	**Sequence homology (to SCoV)**
	Identity: 85.3%; similarity: 95.9%
**7**	**Published structures (SCoV2 or homologue variants)**
	SCoV: PDB 1XAK, 1YO4
**8**	**(Published) assignment (SCoV2 or homologue variants)**
	SCoV: BMRB 6824

Table 2: Protein Expression

**1**	**Expression vector**
	pET24d-GB1 (Novagen, modified by G. Stier (Bogomolovas et al., 2009))
**2**	**Purification-/Solubility-Tag**
	N-terminal His_6-GB1
**3**	**Cleavage Site**
	TEV
**4**	**Molecular weight / Extinction coefficient / pI - of cleaved protein**
	7.49 kDa / 6,210 M^{-1} cm^{-1} / 6.99
**5**	**Comments on sequence of expressed construct**
	N-terminal „G" one artificial residue due to TEV-cleavage and construct design.
**6**	**Used expression strain**

	E.coli (DE3) BL21
**7**	**Cultivation medium**
	M9 (uniformly ^{15}N-labelled)
**8**	**Induction system**
	IPTG inducible T7 promoter
**9**	**Induction of protein expression**
	0.2 mM IPTG at OD_{600} 0.7
**10**	**Cultivation temperature and time**
	25°C for 18-20 h

Table 3: Protein Purification

**1**	**Buffer List**
A	20 mM Tris-HCl (pH 8.0), 6 M GdnHCl, 500 mM NaCl, 5 mM imidazole, 2 mM bME (Cell disruption / solubilization of pellet).
B	20 mM Tris (pH 8.0), 6 M GdnHCl, 500 mM NaCl, 10 mM imidazole, 2 mM bME (IMAC1).
C	50 mM NaPi (pH 8.0), 300 mM NaCl, 10 mM imidazole, 2 mM bME (IMAC2).
D	1 mM acetate-D4 (pH 5.0) (final NMR-buffer).
**2**	**Purification steps (with corresponding buffer(s) and incubation times)**
A	Cell disruption and solubilization of pellet in buffer **1A**.
B	IMAC, gravity flow Ni^{2+}-NTA (Qiagen), elution with 200 mM imidazole in buffer **1B**.
C	Dialysis against buffer **1C**.
D	TEV-cleavage (1 mg TEV protease per 10 mL protein solution) o.n. in buffer **1C**.
E	Inv. IMAC, elution with 200 mM imidazole in buffer **1C**.
F	Dialysis of flow-through of inv. IMAC against **1D** and concentrate (NMR-sample).

Table 4: Final sample

**1**	**Yield**
	0.4 mg/L ^{15}N-M9 medium
**1b**	**A260/280 ratio**
	0.7
**2**	**Stability**
	Stable throughout measurement (1 day, 298/315 K). No precipitation or degradation observed after four days at rt.

3	**Comment on applicability**
	Suitable for NMR structure determination, fragment screening, interaction studies.

SI18: ORF7b

Tabel 1: General Information

1	**Protein Name (according to NCBI Reference Sequence NC_045512.2)**
	ORF7b
2	**Region/Name/Further Specification**
3	**Sequence of fl protein (according to NCBI Reference Sequence NC_045512.2)**
	MIELSLIDFY LCFLAFLLFL VLIMLIIFWF SLELQDHNET CHA
4	**Protein boundaries of expressed construct (according to NCBI Reference Sequence NC_045512.2)**
	aa 1-43 (fl ORF7b)
5	**Ratio for construct design**
	fl protein
6	**Sequence homology (to SCoV)**
	Identity: 85.4%; similarity: 97.2%
7	**Published structures (SCoV2 or homologue variants)**
	-
8	**(Published) assignment (SCoV2 or homologue variants)**
	**-**

Bacterial

Table 2: Protein Expression

1	**Expression vector**
	pThiored (GenScript)
2	**Purification-/Solubility-Tag**
	N-terminal His_6-Trx
3	**Cleavage Site**
	TEV
4	**Molecular weight / Extinction coefficient / pI - of cleaved protein**
	5.37 kDa / 6,990 M^{-1} cm^{-1} / 4.17
5	**Comments on sequence of expressed construct**
	N-terminal “GA(M)G” three artificial residues due to TEV-cleavage and construct design.
6	**Used expression strain**

	E. coli BL21 (DE3)
**7**	**Cultivation medium**
	LB / M9 (uniformly ^{15}N-labelled)
**8**	**Induction system**
	IPTG inducible T7 promoter
**9**	**Induction of protein expression**
	0.2 mM IPTG at OD_{600} 0.7
**10**	**Cultivation temperature and time**
	18-20°C for 16-18 h

Table 3: Protein Purification with detergent

**1**	**Buffer List**
A	25 mM Tris-HCl (pH 8.0), 300 mM NaCl, 5 mM imidazole, 10 mM bME (cell disruption).
B	25 mM Tris-HCl (pH 8.0), 300 mM NaCl, 5 mM imidazole, 10 mM bME, 1.5% (w/v) DDM (Solubilization of pellet).
C	25 mM Tris-HCl (pH 8.0), 300 mM NaCl, 10 mM imidazole, 10 mM bME, 0.02% (w/v) DDM (IMAC).
D	25 mM NaPi (pH 7.0), 150 mM NaCl, 2 mM TCEP-HCl, 0.02% (w/v) DDM (SEC/final NMR buffer).
**2**	**Purification steps (with corresponding buffer(s) and incubation times)**
A	Cell disruption in buffer **1A** (plus one tablet of EDTA free protease inhibitor cocktail (Merck)) by microfluidization.
B	Solubilization of pellet after lysis **1B** (plus one tablet of EDTA free protease inhibitor cocktail (Merck)).
C	IMAC (HisTrap HP (GE Healthcare), ÄKTA start (GE Healthcare)), elution with imidazole gradient up to 500 mM in buffer **1C.**
D	TEV-cleavage (1 mg TEV protease per 50 mL protein solution) o.n. in buffer **1C**
E	Inv. IMAC (HisTrap HP (GE Healthcare), ÄKTA start (GE Healthcare)), elution with 500 mM imidazole in buffer **1C**.
F	Rebuffer flow-through of inv. IMAC in buffer **1D** (NMR sample).
G	Analytical SEC (SD 75 Increase 10/300 GL (GE Healthcare), ÄKTA start (GE Healthcare)) in buffer **1D**.

Table 4: Final sample

**1**	**Yield**
	0.6 mg/L ^{15}N-M9 medium
**2**	**Stability**
	Stable throughout measurement (2 days, 283/298 K). No significant precipitation or degradation observed after storage at 4°C for 3 months.

3	**Comment on applicability**
	Due to necessity of solubilizing agent and tendency to oligomerize structure determination, fragment screening, and interaction studies are hindered.

Additional information

	**Constructs**	**Conditions**	**Comments**
**A**	As above	Native<br>**IMAC buffer:** 25 mM Tris-HCl (pH 8.0), 300 mM NaCl, 5 mM imidazole, 10 mM bME.<br>**SEC buffer:** 25 mM NaPi (pH 7.0), 150 mM NaCl, 2 mM TCEP-HCl	Nearly no protein was extracted in soluble fraction.
**B**		Denaturing<br>**Solubilizing buffer:** 25 mM Tris-HCl (pH 8.0), 6 M GdnHCl, 300 mM NaCl, 5 mM imidazole.<br>**IMAC wash buffer:** 25 mM Tris-HCl (pH 8.0), 8 M urea, 300 mM NaCl, 5 mM imidazole.<br>**Renaturing buffer:** 25 mM Tris-HCl (pH 8.0), 300 mM NaCl, 5 mM imidazole, 10 mM bME.<br>**IMAC elution buffer:** 25 mM Tris-HCl (pH 8.0), 300 mM NaCl, 500 mM imidazole, 10 mM bME.	After refolding and cleavage degradation of protein.
**C**	Fl ORF7b; His_6-SUMO (pE-SUMO (GenScript)), Ulp1-cleavage site, no artificial residues.	Native<br>**IMAC buffer:** as above<br>**SEC buffer:** 25 mM NaPi (pH 7.0), 150 mM NaCl, 2 mM TCEP-HCl.	Protein is soluble with fusion, runs in exclusion volume of SD 200 columns, degrades after cleavage. NMR shows SUMO is mostly unfolded.
**D**		Detergent<br>**IMAC buffer:** 50 mM NaPi (pH 7.0), 200 mM NaCl, 0.1% (v/v) Triton X-100, 5 mM imidazole, 10 mM bME.<br>**SEC buffer:** 25 mM NaPi (pH 6.0), 50 mM NaCl, 0.01% (v/v) Triton X-100, 2 mM TCEP-HCl.	Copurification of impurities, runs in exclusion volume of SD 200 columns.<br>NMR shows severely broadened and poorly dispersed resonances hinting to oligomerization.
**E**		Semi-denaturing<br>**IMAC buffer:** 50 mM Tris-HCl **(pH 8.0)**, 2 M urea, 300 mM NaCl, 10 mM imidazole, 10 mM bME.<br>**SEC buffer:** 25 mM NaPi (pH 6.5), 50 mM NaCl, 2 M urea, 5 mM DTT.	Degrades after cleavage.

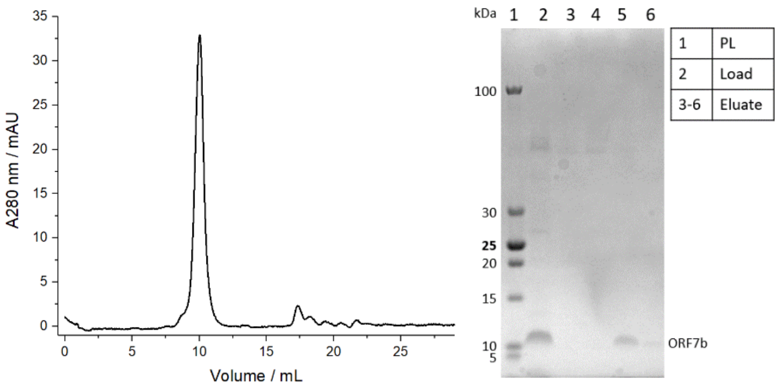

Analytical SEC of ORF7b. Protein was in exclusion volume (9-11 mL, left panel) with corresponding SDS-PAGE of SEC with fractions analyzed from 7-11 mL elution volume (right panel).

Cell-free

Table 2: Cell-free Protein Synthesis

**1**	**Expression vector**
	pEU-E01-MCS (Cell-Free Sciences)
**2**	**Purification-/Solubility-Tag**
	C-terminal Strep tag II (WSHPQFEK)
**3**	**Cleavage Site**
	-
**4**	**Molecular weight / Extinction coefficient / pI - of protein**
	5.37 kDa / 6,990 M^{-1} cm^{-1} / 4.17
**5**	**Comments on sequence of expressed construct**
	C-terminal "SAWSHPQFEK" ten artificial residues due to construct design.
**6**	**Feeding buffer**
	30 mM HEPES-KOH (pH 7.6), 100 mM potassium acetate, 2.7 mM magnesium acetate, 16 mM creatine phosphate, 0.4 mM spermidine, 1.2 mM ATP, 0.25 mM GTP, 4 mM DTT and 6 mM (average concentration) amino acid mix and 0.1% (w/v) MNG-3.
**7**	**Translation mix**
	50% (v/v) mRNA, 50% (v/v) home-made WGE, 40 µg/mL creatine kinase, and 6 mM (average concentration) amino acid mix, 0.1% (w/v) MNG-3.
**8**	**Protein synthesis temperature and time**
	22°C for 16 h without agitation (bilayer method).

Table 3: Protein Purification

**1**	**Buffer List**
A	100 mM Tris-HCl (pH 8.0), 150 mM NaCl, 1 mM EDTA, 0.1% (w/v) DDM (wash buffer).

B	100 mM Tris-HCl (pH 8.0), 150 mM NaCl, 1 mM EDTA, 2.5 mM desthiobiotin, and 0.1% (w/v) DDM (elution buffer).
**2**	**Purification steps (with corresponding buffer(s) and incubation times)**
A	Harvest total CFS.
B	Incubate with benzonase for 30 min on a wheel, at rt.
C	Centrifuge for 30 min at 20,000 g, 4°C.
D	Harvest the soluble fraction (SN).
E	Equilibrate the Strep-Tactin column (IBA Lifesciences) with 2 CV of **1A** (all steps performed on the bench by gravity).
F	Load SN onto the column.
G	Wash the column with 5 CV of **1A**.
H	Elute the protein of interest with **1B**.

Table 4: Final sample

**1**	**Yield**
	0.27 mg/mL of WGE and total production of 880 µg for NMR samples
**1b**	**A260/280 ratio**
	1.36
**2**	**Stability**
	Stable in detergent over several days.
**3**	**Comment on applicability**
	Needs reconstitution into membranes for further structural analysis.

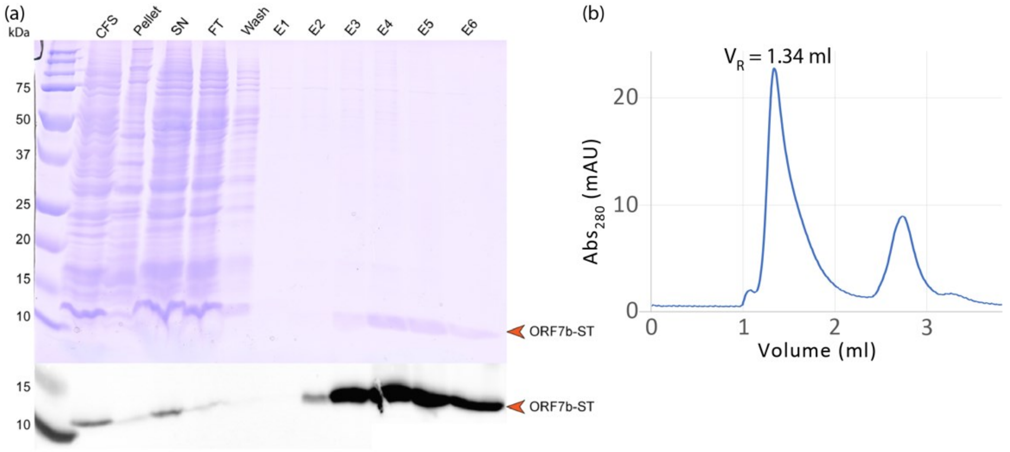

(a) WG-CFPS in presence of detergent and Strep-tag purification of ORF7b. SDS-PAGE (upper panel) and WB (lower panel). **(b) SEC profile of ORF7b.**

SI19: ORF8

Table 1: General Information

**1**	**Protein Name (according to NCBI Reference Sequence NC_045512.2)**
	ORF8
**2**	**Region/Name/Further Specification**
**3**	**Sequence of fl protein (according to NCBI Reference Sequence NC_045512.2)**
	MKFLVFLGIITTVAAFHQECSLQSCTQHQPYVVDDPCPIHFYSKWYIRVGARKSAPLIELCVDEA GSKSPIQYIDIGNYTVSCSPFTINCQEPKLGSLVVRCSFYEDFLEYHDVRVVLDFI
**4**	**Protein boundaries of expressed construct (according to NCBI Reference Sequence NC_045512.2)**
**ORF8**	aa 1-121 (fl ORF8 = ORF8)
**ORF8 m**	aa 1-121 (fl ORF8) with L84S mutation (~ isolate 2019-nCoV_HKU-SZ-002a_2020).
**ΔORF 8**	aa 16-121 (without signal peptide = ΔORF8)
**5**	**Ratio for construct design (detailed and comprehensible)**
**ORF8**	fl protein
**ΔORF 8**	Protein after the hypothetical cleavage of the N-terminal Signal Peptide
**6**	**Sequence homology (to SCoV)**
**ORF8**	Identity: 31.7%; similarity: 70.7%
**ΔORF 8**	Identity: 40.5%; similarity: 66.7%
**7**	**Published structures (SCoV2 or homologue variants)**
	SCoV2: 7JTL, 7JX6
**8**	**(Published) assignment (SCoV2 or homologue variants)**
	-

Bacterial

Table 2: Protein Expression

**1**	**Expression vector**
**ORF8 m**	pPK1154 (GenScript)
**ΔORF 8**	pET22b (+) (Merck/Novagen)
**2**	**Purification-/Solubility-Tag**

**ORF8m**	N-terminal His$_6$-SUMO
**ΔORF8**	N-terminal His$_6$-GST
**3**	**Cleavage Site**
**ORF8m**	Ulp1
**ΔORF8**	TEV
**4**	**Molecular weight / Extinction coefficient / pI - of cleaved protein**
**ORF8m**	13.80 kDa / 15,930 M^{-1} cm^{-1} / 5.42
**ΔORF8**	12.54 kDa / 15,930 M^{-1} cm^{-1} / 5.15
**5**	**Comments on sequence of expressed construct**
**ORF8m**	No artificial residues due to Ulp1-cleavage and construct design.
**ΔORF8**	N-terminal "GAMG" three artificial residues due to TEV-cleavage and construct design.
**6**	**Used expression strain**
**ORF8m**	*E. coli* BL21 (DE3)
**ΔORF8**	*E. coli* BL21 (DE3) pLysS
**7**	**Cultivation medium**
**ORF8m**	LB / M9 (uniformly ^{15}N-labelled)
**ΔORF8**	LB
**8**	**Induction system**
	IPTG inducible T7 promoter
**9**	**Induction of protein expression**
**ORF8m**	0.5 mM IPTG at OD_{600} 0.6
**ΔORF8**	0.5 mM IPTG at OD_{600} 0.6-0.7
**10**	**Cultivation temperature and time**
**ORF8m**	16-20°C for 16-18 h
**ΔORF8**	18°C for 16-18 h

Table 3a: Protein Purification (ORF8)

**1**	**Buffer List**
A	10 mM NaPi (pH 8.0), 300 mM NaCl, 10 mM imidazole, 0.5 mM DTT (Cell disruption).
B	10 mM NaPi (pH 8.0), 300 mM NaCl, 10 mM imidazole, 0.5 mM DTT (Solubilization of pellet).
C	10 mM NaPi (pH 8.0), 300 mM NaCl, 0.5 mM DTT (IMAC).
D	50 mM Tris-HCl (pH 8.0), 150 mM NaCl, 1 mM DTT, 0.2% (w/v) NP40.
E	50 mM Tris-HCl (pH 8.0), 150 mM NaCl, 1 mM DTT.
**2**	**Purification steps (with corresponding buffer(s) and incubation times)**
A	Cell disruption in buffer **1A** (plus one tablet of EDTA free protease inhibitor cocktail (Merck)) by French-press.
B	Solubilization of pellet after lysis **1B** (plus one tablet of EDTA free protease inhibibitor cocktail (Merck)).
C	IMAC (Nickel-NTA-Agarose, QIAGEN) by hand, elution with 250 mM imidazole in buffer **1C**.
D	Ulp1-cleavage (Protein/Ulp1 ratio 10:1) o.n. at 21°C in buffer **1D**.
E	Rebuffer in buffer **1E**.

Table 3b: Protein Purification (ΔORF8)

**1**	**Buffer List**
A	50 mM Tris-HCl (pH 8.0), 500 mM NaCl, 5% (v/v) glycerol, 50 mM imidazole (cell disruption/IMAC).
B	50 mM Tris-HCl (pH 8.0), 150 mM NaCl (TEV-cleavage).
C	20 mM NaPi (pH 7.4), 150 mM NaCl, 1 mM EDTA (SEC final buffer).
**2**	**Purification steps (with corresponding buffer(s) and incubation times)**
A	Cell disruption in buffer **1A** (supplemented with 0.5 mg/mL lysozyme, 10 µg/mL DNaseI, 5 mM $MgCl_2$, cOmplete™ EDTA-free protease inhibitors) by incubation for 30 min at RT followed by sonication at 43% amplitude for 2 minutes (1 s on, 1 s off). Extraction of the periplasmatic fraction: added 0.1% (v/v) Triton to the total sample after sonication, and incubated 15 min at 4°C. Centrifugation at 24.700 g for 40 min at 4°C. Recovering of the soluble fraction and filtration using 0.45 µm syringe filters.
B	IMAC (HisTrap FF Crude (GE Healthcare), ÄKTA Pure 25 M1 (GE Healthcare)), binding with buffer **1A** supplemented with 50 mM imidazole, elution with imidazole gradient up to 500 mM in buffer **1A**.
C	TEV-cleavage (Protein/TEV ratio 1:10) at 4°C, o.n. in buffer **1B**.
D	Inv. IMAC (HisTrap FF Crude (GE Healthcare), ÄKTA Pure 25 M1 (GE Healthcare)), binding with buffer **1A** supplemented with 50 mM imidazole, elution with imidazole gradient up to 500 mM in buffer **1A**.
E	SEC on Increase 10/300 S75 (GE Healthcare) at 4°C in buffer **1C**.

Table 4: Final sample

**1**	**Yield**
**ORF8 m**	<0.5 mg/L LB mg/mL ^{15}N-M9 medium

**ΔORF 8**	0.5 mg/L LB medium
**2**	**Stability**
**ORF8 m**	Not determined.
**ΔORF 8**	No significant precipitation or degradation observed after storage at 4°C for 1 week.
**3**	**Comment on applicability**
**ORF8 m**	Weak expression into solube fraction, 30%/70% soluble/inclusion bodies. After purification extremely low yield for NMR studies.
**ΔORF 8**	Very low yield. It would be very expensive to prepare a labelled sample for NMR studies.

Additional information (bacterial expression)

**Constructs**	**Conditions**	**Comments**
ORF8 with L84S mutation; His_6 (pPK1151 (Genscript)), TEV-cleavage site, N-terminal "GS" two artificial residues.	As above for ORF8m, only LB medium.	No expression.

Cell-free

Table 2: Cell-free Protein Synthesis

**1**	**Expression vector**
	pEU-E01-MCS (Cell-Free Sciences)
**2**	**Purification-/Solubility-Tag**
**ORF8**	C-terminal Strep tag II (WSHPQFEK)
**ΔORF 8**	N-terminal Strep tag II (WSHPQFEK)
**3**	**Cleavage Site**
	-
**4**	**Molecular weight / Extinction coefficient / pI - of cleaved protein**
**ORF8**	15.00 kDa / 21,805 M^{-1} cm^{-1} / 5.64
**ΔORF 8**	13.53 Da / 21,805 M^{-1} cm^{-1} / 5.39
**5**	**Comments on sequence of expressed construct**
**ORF8**	C-terminal "SAWSHPQFEK" ten artificial residues due to construct design.
**ΔORF 8**	N-terminal "M" and C-terminal "SAWSHPQFEK" eleven artificial residues due to construct design.

**6**	**Feeding buffer**
	30 mM HEPES-KOH (pH 7.6), 100 mM potassium acetate, 2.7 mM magnesium acetate, 16 mM creatine phosphate, 0.4 mM spermidine, 1.2 mM ATP, 0.25 mM GTP, 4 mM DTT and 6 mM (average concentration) amino acid mix and 0.05% (w/v) Brij-58.
**7**	**Translation mix**
	50% (v/v) mRNA, 50% (v/v) home-made WGE, 40 µg/mL creatine kinase, and 6 mM (average concentration) amino acid mix 0.05% (w/v) Brij-58.
**8**	**Protein synthesis temperature and time**
	22°C for 16 h without agitation (bilayer method).

Table 3: Protein Purification (ORF8a and ORF8b)

**1**	**Buffer List**
A	100 mM Tris-HCl (pH 8.0), 150 mM NaCl, 1 mM EDTA, 0.1% (w/v) DDM (wash buffer).
B	100 mM Tris-HCl (pH 8.0), 150 mM NaCl, 1 mM EDTA, 2.5 mM desthiobiotin, and 0.1% (w/v) DDM (elution buffer).
**2**	**Purification steps (with corresponding buffer(s) and incubation times)**
A	Harvest total CFS.
B	Incubate with benzonase for 30 min on a wheel, at rt.
C	Centrifuge for 30 min at 20,000 g, 4°C.
D	Harvest the soluble fraction (SN).
E	Equilibrate the Strep-Tactin column (IBA Lifesciences) with 2 CV of **1A** (all steps performed on the bench by gravity).
F	Load SN onto the column.
G	Wash the column with 5 CV of **1A**.
H	Elute the protein of interest with **1B**.

Table 4: Final sample

**1**	**Yield**
	0.62 mg/mL WGE after purification. Total of 683 µg for the NMR samples
**1b**	**A260/280 ratio**
	0.7
**2**	**Stability**
	Stable at 4ºC for weeks.
**3**	**Comment on applicability**
	Protein very sensitive to dilution-concentration steps. Purity is sufficient for NMR as other cell-free proteins are not labelled.

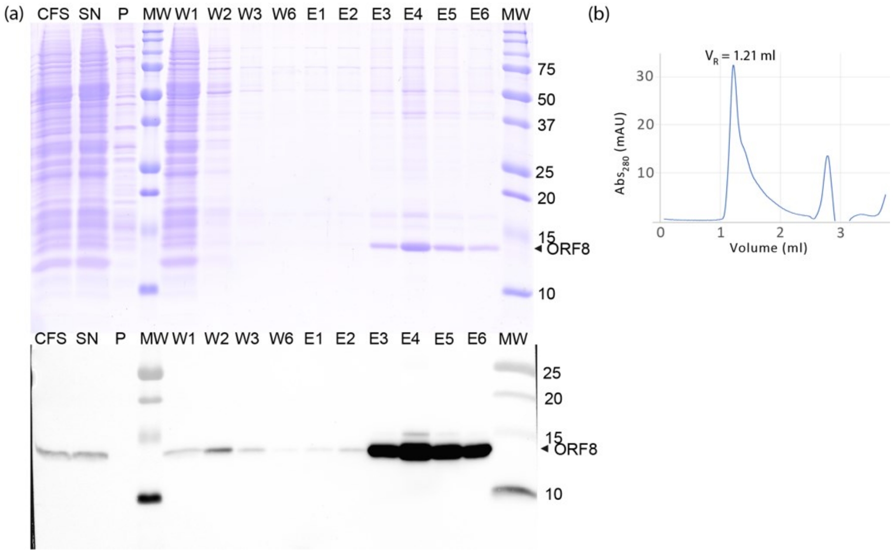

(a) WG-CFPS in presence of detergent and Strep-tag purification of ORF8. SDS-PAGE (upper panel) and WB (lower panel). **(b) SEC profile of ORF8.**

SI20: ORF9a (Nucleocapsid (N) protein)

Table 1: General Information

1	**Protein Name (according to NCBI Reference Sequence NC_045512.2)**
	ORF9a; Nucleocapsid (N) phosphoprotein
2	**Region/Name/Further Specification**
	N-terminal disordered region (aa 1-43, IDR1) / N-terminal RNA binding domain (aa 44-180, NTD) / serine-arginine (SR) rich motif (aa 181-212, SR) / central disordered linker (aa 181-248, IDR2) / C-terminal dimerization domain (247-364) / C-terminal disordered region (aa 365-419, IDR3)
3	**Sequence of fl protein (according to NCBI Reference Sequence NC_045512.2)**
	MSDNGPQNQRNAPRITFGGPSDSTGSNQNGERSGARSKQRRPQGLPNNTASWFTALTQHGKED<br>LKFPRGQGVPINTNSSPDDQIGYYRRATRRIRGGDGKMKDLSPRWYFYYLGTGPEAGLPYGAN<br>KDGIIWVATEGALNTPKDHIGTRNPANNAAIVLQLPQGTTLPKGFYAEGSRGGSQASSRSSSRSR<br>NSSRNSTPGSSRGTSPARMAGNGGDAALALLLLDRLNQLESKMSGKGQQQQGQTVTKKSAAE<br>ASKKPRQKRTATKAYNVTQAFGRRGPEQTQGNFGDQELIRQGTDYKHWPQIAQFAPSASAFFG<br>MSRIGMEVTPSGTWLTYTGAIKLDDKDPNFKDQVILLNKHIDAYKTFPPTEPKKDKKKKADET<br>QALPQRQKKQQTVTLLPAADLDDFSKQLQQSMSSADSTQA
4	**Protein boundaries of expressed construct (according to NCBI Reference Sequence NC_045512.2)**
**IDR1-NTD-IDR2**	aa 1-248 (of fl ORF9a)
**NTD-SR**	aa 44-212 (of fl ORF9a)
**NTD**	aa 44-180 (of fl ORF9a)
**CTD**	aa 247-364 (of fl ORF9a)
5	**Ratio for construct design (detailed and comprehensible)**
**IDR1-NTD-IDR2**	Based on boundaries from SCoV homolog.
**NTD-SR**	In analogy to the available NMR (PDB 6YI3) and crystal (6M3M) structures of N-NTD SCoV2.
**NTD**	In analogy to the available NMR (PDB 6YI3) and crystal (6M3M) structures of N-NTD SCoV2.
**CTD**	In analogy to the available NMR structure (PDB 2JW8) of N-CTD from SCoV.
6	**Sequence homology (to SCoV)**
**IDR1-NTD-IDR2**	Identity: 90%; similarity: 94%
**NTD-SR**	Identity: 92%; similarity: 96%
**NTD**	Identity: 93%; similarity: 97%
**CTD**	Identity: 96%; similarity: 98%
7	**Published structures (SCoV2 or homologue variants)**

	SCoV: PDB 2JW8, 2CJR<br>SCoV2: PDB 6YI3, 6M3M, 6VYO, 6WKP, 6WZO, 6WJI, 6YUN, 6ZCO, 7CE0, 7C22
**8**	**(Published) assignment (SCoV2 or homologue variants)**
	SCoV: BMRB 15511 (CoV)<br>SCoV2: PDB 6YI3, BMRB 34511 (NTD), BMRB 50518 (CTD), BRMB 50619 (IDR1), BMRB 50618 (IDR2), BMRB 50557 (IDR1), BMRB 50558 (IDR2).

Table 2: Protein Expression

**1**	**Expression vector**
**IDR1-NTD-IDR2**	pET29a(+) (Twistbioscience)
**NTD-SR**	pET-28a(+) (GenScript)
**NTD**	pET-28a(+) (GenScript)
**CTD**	pKM263 (GenScript)
**2**	**Purification-/Solubility-Tag**
**IDR1-NTD-IDR2**	-
**NTD-SR**	N-terminal His_6
**NTD**	N-terminal His_6
**CTD**	N-terminal His_6-GST
**3**	**Cleavage Site**
**IDR1-NTD-IDR2**	-
**NTD-SR**	TEV
**NTD**	TEV
**CTD**	TEV
**4**	**Molecular weight / Extinction coefficient / pI - of cleaved protein**
**IDR1-NTD-IDR2**	26.52 kDa / 26,930 M^{-1} cm^{-1} / 10.57
**NTD-SR**	18.10 kDa / 26,930 M^{-1} cm^{-1} / 10.35
**NTD**	14.85 kDa / 26,930 M^{-1} cm^{-1} / 9.60
**CTD**	13.56 kDa / 16,960 M^{-1} cm^{-1} / 9.77

**5a**	**Comments on sequence of expressed construct**
**IDR1-NTD-IDR2**	No artificial residues due to construct design.
**NTD-SR**	No artificial residues due to TEV-cleavage and construct design.
**NTD**	No artificial residues due to TEV-cleavage and construct design.
**CTD**	N-terminal „GAMG" four artificial residues due to TEV-cleavage and construct design.
**6**	**Used expression strain**
	E. coli BL21 (DE3)
**7**	**Cultivation medium**
**IDR1-NTD-IDR2**	LB / M9 (uniformly ^{15}N or ^{13}C,^{15}N-labelled)
**NTD-SR**	LB / M9 (uniformly ^{15}N-labelled)
**NTD**	LB / M9 (uniformly ^{15}N-labelled)
**CTD**	LB / M9 (uniformly ^{15}N or ^{13}C,^{15}N-labelled)
**8**	**Induction system**
	IPTG inducible T7 promoter
**9**	**Induction of protein expression**
**IDR1-NTD-IDR2**	0.2 mM IPTG at OD_{600} 0.8
**NTD-SR**	0.2 mM IPTG at OD_{600} 0.7
**NTD**	0.2 mM IPTG at OD_{600} 0.7
**CTD**	1 mM IPTG at OD_{600} 0.7
**10**	**Cultivation temperature and time**
**IDR1-NTD-IDR2**	Cells are grown at 37°C in 1 L LB until OD_{600} 0.8, then transferred in 250 mL labelled minimal medium (4x). After 1 h of metabolite clearance, the culture is induced at 18°C for 16-18 h. For unlabelled protein, culture is induced at OD_{600} 0.9.
**NTD-SR**	16-18°C for 16-18 h
**NTD**	16-18°C for 16-18 h
**CTD**	20-22°C for 18-20 h

Table 3a: Protein Purification (IDR1-NTD-IDR2)

**1**	**Buffer List**
A	25 mM Tris-HCl (pH 8.0), 1 M NaCl, 5% (v/v) glycerol, RNAse, DNAse, proteases inhibitor cocktail (SIGMA*FAST*™ tablet, 500 µL of 100x stock) (lysis buffer).
B	25 mM Tris-HCl (pH 7.2) (dialysis after lysis and binding buffer).
C	25 mM Tris-HCl (pH 7.2), 1 M NaCl (elution buffer).
D	25 mM Tris-HCl (pH 7.2), 450 mM NaCl, 0.02% (w/v) NaN_3 (NMR buffer).
**2**	**Purification steps (with corresponding buffer(s) and incubation times)**
A	Cell lysis in Buffer **1A** by sonication (30 min with pulse 1 s on, 10 s off). It is crucial to add a cocktail of proteases inhibitors in lysis buffer; this step is crucial to preserve construct integrity.
B	Dialysis O/N at 4°C in Buffer **1B** for buffer exchange.
C	Ion Exchange chromatography with HiTrap SP FF 5 mL column (GE Healthcare), gradient elution with buffer **1C**. The protein eluted at 45-50% gradient.

Table 3b: Protein Purification (NTD and NTD-SR)

**1**	**Buffer List**
A	50 mM Tris-HCl (pH 8.0), 500 mM NaCl, 20 mM imidazole, 10% (v/v) glycerol, 0.01 mg/mL DNase, 5 mM $MgCl_2$ and protease inhibitor cocktail (Sigma) (cell disruption).
B	50 mM Tris-HCl (pH 8.0), 500 mM NaCl, 20 mM imidazole, 10% (v/v) glycerol (IMAC).
C	50 mM Tris-HCl (pH 8.0), 500 mM NaCl, 500 mM imidazole, 10% (v/v) glycerol (IMAC).
D	50 mM Tris-HCl (pH 8.0), 500 mM NaCl, 1 mM DTT (dialysis after IMAC / TEV-cleavage).
E	20 mM Na_2HPO_4 (pH 6.5), 50 mM NaCl, 500 µM PMSF, 3 mM NaN_3, 3 mM EDTA (final NMR buffer).
**2**	**Purification steps (with corresponding buffer(s) and incubation times)**
A	Cell disruption in buffer **1A** by sonication.
B	IMAC (HisTrap HP (GE Healthcare), ÄKTA start (GE Healthcare)), elution with imidazole gradient up to 500 mM in buffer **1B** and **1C**.
C	TEV-cleavage (1:10 (v/v) TEV:protein solution) during dialysis o.n. in buffer **1D**.
D	Inv. IMAC (HisTrap HP (GE Healthcare), ÄKTA start (GE Healthcare)), elution with imidazole gradient up to 500 mM in buffer **1B** and **1C**.
E	NMR sample preparation in buffer **1E**.

Table 3c: Protein Purification (CTD)

**1**	**Buffer List**
A	50 mM NaPi (pH 7.4), 150 mM NaCl, 10 mM imidazole (cell disruption / IMAC/ dialysis after IMAC / TEV-cleavage).
B	25 mM NaPi (pH 6.0), 50 mM NaCl, 0.5 mM EDTA, 0.02% (w/v) NaN_3 (SEC / final NMR buffer).
**2**	**Purification steps (with corresponding buffer(s) and incubation times)**

A	Cell disruption in buffer **1A** (plus 100 µL protease inhibitor (Serva)) by sonication.
B	IMAC (gravity flow Ni^{2+}-NTA), Elution with 150-500 mM imidazole in buffer **1A**.
C	Dialysis o.n. in in buffer **1A**.
D	TEV-cleavage (0.5 mg TEV protease per 1 L culture) in buffer **1A**.
E	SEC on HiLoad 16/600 SD 75 (GE Healthcare) in buffer **1B**.
F	NMR sample preparation in buffer **1B**.

Table 4: Final sample

**1**	**Yield**
**IDR1-NTD-IDR2**	12 mg/L ^{13}C,^{15}N M9 medium
**NTD-SR**	3 mg/L ^{15}N M9 medium
**NTD**	3 mg/L ^{15}N M9 medium
**CTD**	2 mg/L ^{13}C,^{15}N-M9 medium
**1b**	**A260/280 ratio**
**IDR1-NTD-IDR2**	0.63
**NTD-SR**	0.7
**NTD**	0.7
**CTD**	0.55
**2**	**Stability**
**IDR1-NTD-IDR2**	Protein is stable for at least one1 week at working conditions (298 K).
**NTD-SR**	Stable throughout measurement (15 days, 298 K). No significant precipitation or degradation observed after storage at 4°C for 5 weeks.
**NTD**	Stable throughout measurement (15 days, 298 K). No significant precipitation or degradation observed after storage at 4°C for 5 weeks.
**CTD**	Stable throughout measurement (7 days, 303 K). No significant precipitation or degradation observed after storage at 4°C for 8 weeks. Tolerates temperature up to 315 K.
**3**	**Comment on applicability**
	All suitable for NMR structure determination, fragment screening, interaction studies.

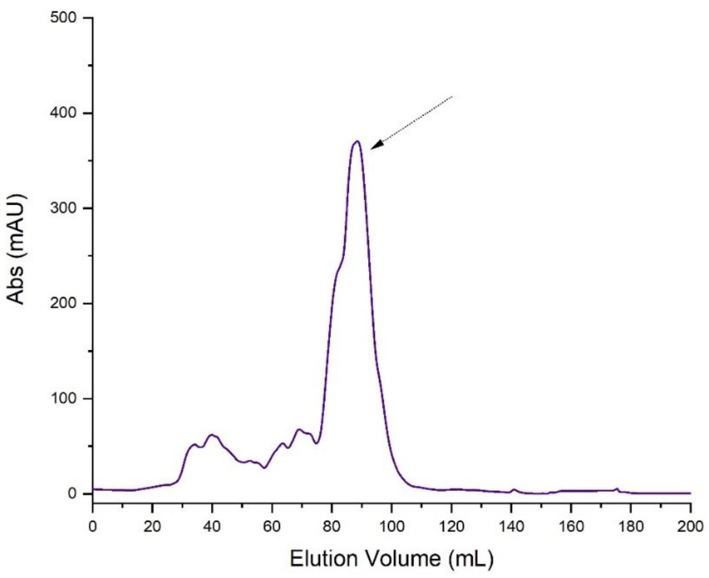

Chromatogram of IEC of aa 1-248 construct. Protein is eluted at 45% gradient of Buffer 1B, fractions from 85-100 mL were collected.

SI21: ORF9b

Table 1: General Information

1	**Protein Name**
	ORF9b
2	**Region/Name/Further Specification**
3	**Sequence of fl protein**
	MDPKISEMHP ALRLVDPQIQ LAVTRMENAV GRDQNNVGPK VYPIILRLGS PLSLNMARKT LNSLEDKAFQ LTPIAVQMTK LATTEELPDE FVVVTVK
4	**Protein boundaries of expressed construct**
	aa 1-97 (fl ORF9b)
5	**Ratio for construct design**
	fl protein
6	**Sequence homology (to SCoV)**
	Identity : 72.4%; similarity: 95.0%
7	**Published structures (SCoV2 or homologue variants)**
	SCoV2: PDB 6Z4U
8	**(Published) assignment (SCoV2 or homologue variants)**
	**-**

Table 2: Cell-free Protein Synthesis

1	**Expression vector**
	pEU-E01-MCS (Cell-Free Sciences)
2	**Purification-/Solubility-Tag**
	C-terminal Strep tag II (WSHPQFEK)
3	**Cleavage Site**
	-
4	**Molecular weight / Extinction coefficient / pI - of protein**
	11.99 kDa / 6,990 M^{-1} cm^{-1} / 6.73
5	**Comments on sequence of expressed construct**
	C-terminal “SAWSHPQFEK” ten artificial residues due to construct design.
6	**Feeding buffer**

	30 mM HEPES-KOH (pH 7.6), 100 mM potassium acetate, 2.7 mM magnesium acetate, 16 mM creatine phosphate, 0.4 mM spermidine, 1.2 mM ATP, 0.25 mM GTP, 4 mM DTT and 6 mM (average concentration) amino acid mix
**7**	**Translation mix**
	50% (v/v) mRNA, 50% (v/v) home-made WGE, 40 µg/mL creatine kinase, and 6 mM (average concentration) amino acid mix
**8**	**Protein synthesis temperature and time**
	22°C for 16 h without agitation (bilayer method).

Table 3: Protein Purification

**1**	**Buffer List**
A	100 mM Tris-HCl (pH 8.0), 150 mM NaCl, 1 mM EDTA.
B	100 mM Tris-HCl (pH 8.0), 150 mM NaCl, 1 mM EDTA, 2.5 mM desthiobiotin.
**2**	**Purification steps (with corresponding buffer(s) and incubation times)**
A	Harvest total CFS.
B	Incubate with benzonase for 30 min on a wheel, at rt.
C	Centrifuge for 30 min at 20,000 g, 4°C.
D	Harvest the soluble fraction (SN).
E	Equilibrate the Strep-Tactin column (IBA Lifesciences) with 2 CV of **1A** (all steps performed on the bench by gravity).
F	Load SN onto the column.
G	Wash the column with 5 CV of **1A**.
H	Elute the protein of interest with **1B**.

Table 4: Final sample

**1**	**Yield**
	0.64 mg/mL WGE and total production of 1338 µg for NMR samples.
**1b**	**A260/280 ratio**
	0.76
**2**	**Stability**
	Stable at 4ºC for a week.
**3**	**Comment on applicability**
	Protein studied at pH 6, 7.5 and pH 8. Methionine gets oxidized without DTT in the buffer.

Additional information

	**Constructs**	**Conditions**	**Comments**
**A**	Fl ORF9b; Strep tag II (pEU-E01-MCS (Cell-Free Sciences)); no cleavage site; C-terminal "WSHPQFEK" eight artificial residues.	As above with 0.1% (w/v) DDM	NMR shows severely broadened resonances due to oligomerization or protein micelles.
**B**		As above without DTT	Methionines get oxidated.

SI22: ORF14

Table 1: General Information

1	**Protein Name**
	ORF14
2	**Region/Name/Further Specification**
3	**Sequence of fl protein**
	MLQSCYNFLKEQHCQKASTQKGAEAAVKPLLVPHHVVATVQEIQLQAAVGELLLLEWLAMA VMLLLLCCCLTD
4	**Protein boundaries of expressed construct**
	aa 1-73 (fl ORF14)
5	**Ratio for construct design**
	fl protein
6	**Sequence homology (to SCoV)**
	Identity: NA; similarity: NA
7	**Published structures (SCoV2 or homologue variants)**
	-
8	**(Published) assignment (SCoV2 or homologue variants)**
	-

Table 2: Cell-free Protein Synthesis

1	**Expression vector**
	pEU-E01-MCS (Cell-Free Sciences)
2	**Purification-/Solubility-Tag**
	N-terminal Strep tag II (WSHPQFEK)
3	**Cleavage Site**
	-
4	**Molecular weight / Extinction coefficient / pI - of protein**
	9.26 kDa / 12,490 M^{-1} cm^{-1} / 6.01
5	**Comments on sequence of expressed construct**
	N-terminal “WSHPQFEKGGG” eleven artificial residues due to construct design.
6	**Feeding buffer**

	30 mM HEPES-KOH (pH 7.6), 100 mM potassium acetate, 2.7 mM magnesium acetate, 16 mM creatine phosphate, 0.4 mM spermidine, 1.2 mM ATP, 0.25 mM GTP, 4 mM DTT and 6 mM (average concentration) amino acid mix and 0.05% (w/v) Brij-58.
**7**	**Translation mix**
	50% (v/v) mRNA, 50% (v/v) home-made WGE, 40 µg/mL creatine kinase, and 6 mM (average concentration) amino acid mix 0.05% (w/v) Brij-58.
**8**	**Protein synthesis temperature and time**
	22°C for 16 h without agitation (bilayer method).

Table 3: Protein Purification

**1**	**Buffer List**
A	100 mM Tris-HCl (pH 8.0), 150 mM NaCl, 1 mM EDTA, 0.1% (w/v) DDM (wash buffer).
B	100 mM Tris-HCl (pH 8.0), 150 mM NaCl, 1 mM EDTA, 2.5 mM desthiobiotin, and 0.1% (w/v) DDM (elution buffer).
**2**	**Purification steps (with corresponding buffer(s) and incubation times)**
A	Harvest total CFS.
B	Incubate with benzonase for 30 min on a wheel, at rt.
C	Centrifuge for 30 min at 20,000 g, 4°C.
D	Harvest the soluble fraction (SN).
E	Equilibrate the Strep-Tactin column (IBA Lifesciences) with 2 CV of **1A** (all steps performed on the bench by gravity).
F	Load SN onto the column.
G	Wash the column with 5 CV of **1A**.
H	Elute the protein of interest with **1B**.

Table 4: Final sample

**1**	**Yield**
	0.43 mg/mL WGE
**1b**	**A260/280 ratio**
	1.06
**2**	**Stability**
	protein has proved unstable during lipid insertion using cyclodextrin for detergent removal
**3**	**Comment on applicability**
	Solution NMR shows severely broadened resonances hinting to oligomerization or too big protein micelles. Lipid reconstitution is ongoing.

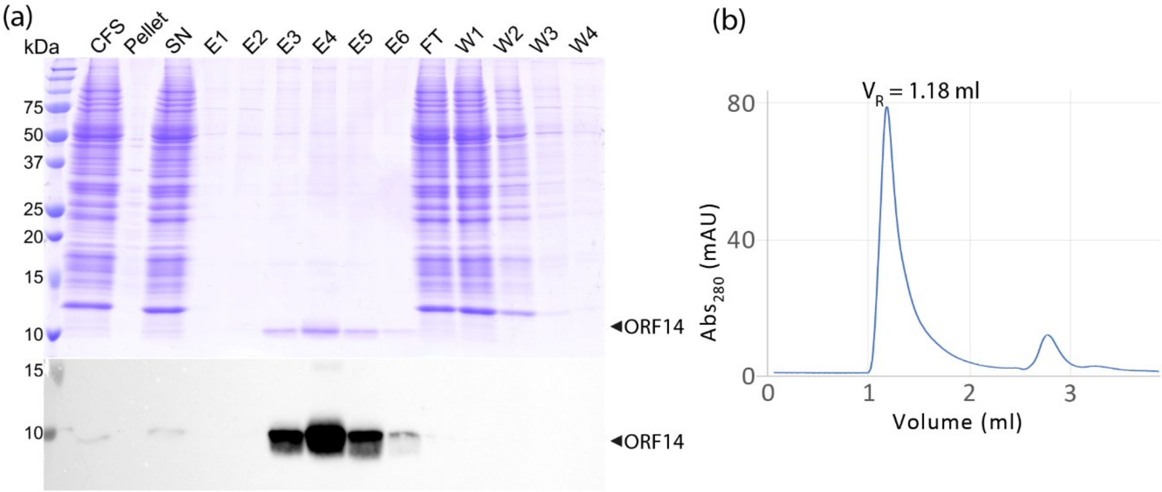

(a) WG-CFPS in presence of detergent and Strep-tag purification of ORF14. SDS-PAGE (upper panel) and WB (lower panel). **(b) SEC profile of ORF14.**

SI23: ORF10

Table 1: General Information

**1**	**Protein Name (according to NCBI Reference Sequence NC_045512.2)**
	ORF10
**2**	**Region/Name/Further Specification**
**3**	**Sequence of fl protein (according to NCBI Reference Sequence NC_045512.2)**
	MGYINVFAFPFTIYSLLLCRMNSRNYIAQVDVVNFNLT
**4**	**Protein boundaries of expressed construct (according to NCBI Reference Sequence NC_045512.2)**
	aa 1-38 (fl ORF10)
**5**	**Ratio for construct design**
	Hypothetical fl protein.
**6**	**Sequence homology (to SCoV)**
	Identity: 29%; similarity: 52% with ORF9b
**7**	**Published structures (SCoV2 or homologue variants)**
	-
**8**	**(Published) assignment (SCoV2 or homologue variants)**
	-

Table 2: Protein Expression

**1**	**Expression vector**
	pThiored (GenScript)
**2**	**Purification-/Solubility-Tag**
	N-terminal His_6-Trx
**3**	**Cleavage Site**
	TEV
**4**	**Molecular weight / Extinction coefficient / pI - of cleaved protein**
	4.45 kDa / 4,470 M^{-1} cm^{-1} / 7.93
**5**	**Comments on sequence of expressed construct**
	N-terminal “GA” two artificial residues due to TEV-cleavage and construct design
**6**	**Used expression strain**
	E. coli BL21 (DE3)

**7**	**Cultivation medium**
	LB / M9 (uniformly ^{15}N-labelled)
**8**	**Induction system**
	IPTG inducible T7 promoter
**9**	**Induction of protein expression**
	0.2 mM IPTG at OD_{600} 0.6-0.7
**10**	**Cultivation temperature and time**
	18-20°C for 16-18 h

Table 3: Protein Purification

**1**	**Buffer List**
A	25 mM Tris (pH 8.0), 6 M GdnHCl, 300 mM NaCl, 5 mM imidazole (Solubilization)
B	25 mM Tris (pH 8.0), 8 M urea, 300 mM NaCl, 5 mM imidazole (IMAC - wash)
C	25 mM Tris (pH 8.0), 300 mM NaCl, 5 mM imidazole, 10 mM bME (IMAC - elution)
D	25 mM NaPi (pH 7.0), 150 mM NaCl, 2 mM TCEP-HCl.
**2**	**Purification steps (with corresponding buffer(s) and incubation times)**
A	Solubilization of cell pellet and inclusion bodies in **1A** (plus one tablet of EDTA free protease inhibitor cocktail (Merck)).
B	IMAC (HisTrap HP (GE Healthcare), ÄKTA start (GE Healthcare)), washed with buffer **1B**, refolded on column in buffer **1C**, elution with imidazole gradient up to 500 mM in buffer **1C**.
C	Analytic TEV-cleavage (1 mg TEV protease per 50 mL protein solution) o.n. in buffer **1C**.
D	Analytical SEC (SD 75 Increase 10/300 GL (GE Healthcare), ÄKTA start (GE Healthcare)) in buffer **1D**.

Table 4: Final sample

**1**	**Yield**
	2 mg/L (^{15}N-M9) His_6-SUMO-fused
**2**	**Stability**
	Degrades after cleavage
**3**	**Comment on applicability**
	Tendency to oligomerize (exclusion volume of SD 75 column).

Additional information

	Constructs	Conditions	Comments
**A**	As above	Native<br>**IMAC buffer:** 25 mM Tris-HCl (pH 8.0), 300 mM NaCl, 5 mM imidazole, 10 mM bME.<br>**SEC buffer:** 25 mM NaPi (pH 7.0), 150 mM NaCl, 2 mM TCEP-HCl.	Nearly no protein was extracted in soluble fraction (in inclusion bodies)
**B**	Fl ORF10; His$_6$-SUMO (pE-SUMO (GenScript)), Ulp1-cleavage site, no artificial residues.	Native<br>**IMAC buffer:** as above<br>**SEC buffer:** 25 mM NaPi (pH 7.0), 150 mM NaCl, 2 mM TCEP-HCl.	Protein is mostly soluble with fusion, partial degradation (copurification of His$_6$-SUMO), runs in exclusion volume of SD 200 columns, degrades after cleavage. NMR shows SUMO is mostly unfolded.
**C**		Detergent<br>**IMAC buffer:** 50 mM NaPi (pH 7.0), 200 mM NaCl, 0.1% (v/v) Triton X-100, 5 mM imidazole, 10 mM bME.<br>**SEC buffer:** 25 mM NaPi (pH 6.0), 50 mM NaCl, 0.01% (v/v) Triton X-100, 2 mM TCEP-HCl.	Copurification of impurities, runs in exclusion volume of SD 75 columns hinting to oligomerization. Degrades after cleavage.
**D**		Semi-denaturing<br>**IMAC buffer:** 50 mM Tris-HCl **(pH 8.0)**, 2 M urea, 300 mM NaCl, 10 mM imidazole, 10 mM bME.<br>**SEC buffer:** 25 mM NaPi (pH 6.5), 50 mM NaCl, 2 M urea, 5 mM DTT.	Degrades after cleavage.

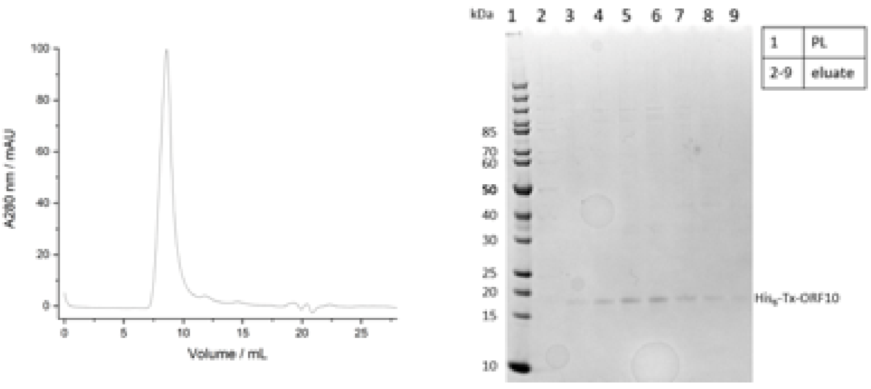

Analytical SEC of His$_6$-Trx-ORF10. Protein was in exclusion volume (8.5-12 mL, left panel) with corresponding SDS-PAGE of SEC with fractions analyzed from 8-12 mL elution volume (right panel).
